# Generalized Analysis of Electrophilic Small Molecules

**DOI:** 10.1002/anie.8895173

**Published:** 2026-05-25

**Authors:** Uma Neelakantan, Maowei Hu, Jiya Bhatia, Huyen N. Nguyen, John (Bobby) Yeboah, Eirinaios I. Vrettos, Dionysius Copoulos, Khue N. M. Nguyen, Madan M. Babu, Daniel J. Blair

**Affiliations:** ^1^ Department of Chemical Biology and Therapeutics St. Jude Children's Research Hospital Memphis Tennessee USA; ^2^ Center of Excellence for Data Driven Discovery, Department of Structural Biology St. Jude Children's Research Hospital Memphis Tennessee USA; ^3^ Department of Pharmaceutical Sciences College of Pharmacy University of Tennessee Health Science Center Memphis Tennessee USA

**Keywords:** acoustic ejection, covalent inhibitor, electrophilic warhead, high‐throughput experimentation, mass‐spectrometry, tandem mass‐spectrometry

## Abstract

Chemical construction almost universally employs highly customized product‐specific analytics. A transition toward generality will remove this critical bottleneck. Here we present how the fundamental chemical property of electrophilicity can be leveraged to generalize reaction analytics. By using a simple thiol probe we transform customized analytical schemes for electrophilic small molecules into a single generalized readout by tandem mass spectrometry. We apply this strategy to acrylamides commonly found in covalent inhibitors, and subsequently show how it can be extended to other classes of electrophilic small molecules. Application of this approach to high‐throughput chemical synthesis streamlines analysis, enabling rapid readouts of chemical analogs featuring acrylamides.

## Graphical abstract

Generalized fragmentation patterns stand to unlock the utility of tandem‐mass spectrometry for chemical reaction analysis but are bottlenecked by time consuming method development. Here were show how we can analyze most electrophilic small molecules the same way by their conjugation with a simple MS/MS encoded chemical probe.

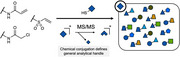

The rise of automation [1] and microtiter plate‐based workflows [2, 3, 4, 5, 6, 7] have erased much of the opportunity cost associated with interrogating vast reaction condition spaces. High‐throughput experimentation (HTE) [8, 9, 10] now enables chemists to evaluate hundreds to thousands [11, 12] of reactions in parallel at microliter scales, systematically varying catalysts, ligands, bases, solvents, concentrations, and temperatures to uncover reactivity trends. The critical advantage of these modern approaches are that they compress design‐make–test cycles while conserving precious substrates and catalysts. Despite numerous impactful advances [13, 14, 15, 16, 17, 18] reaction analysis remains a primary rate‐limiter and the least generalized component of HTE. Most campaigns rely on bespoke, reaction‐specific assays or chromatographic methods that demand significant development time and revalidation for new substrate classes (Figure 1a). There is a need for analysis frameworks that are (i) minimally tuned yet broadly applicable, (ii) robust to plate and matrix effects, and (iii) compatible with the pace of modern high throughput synthesis (hundreds–thousands of reactions/day). Developing such generalized analytical methods would unlock more reliable structure reactivity maps [19], accelerate discovery [20, 21], empower AI/ML model building [22, 23, 24, 25, 26] and improve the fidelity of translating microliter reactions to preparative scales.

**FIGURE 1:**
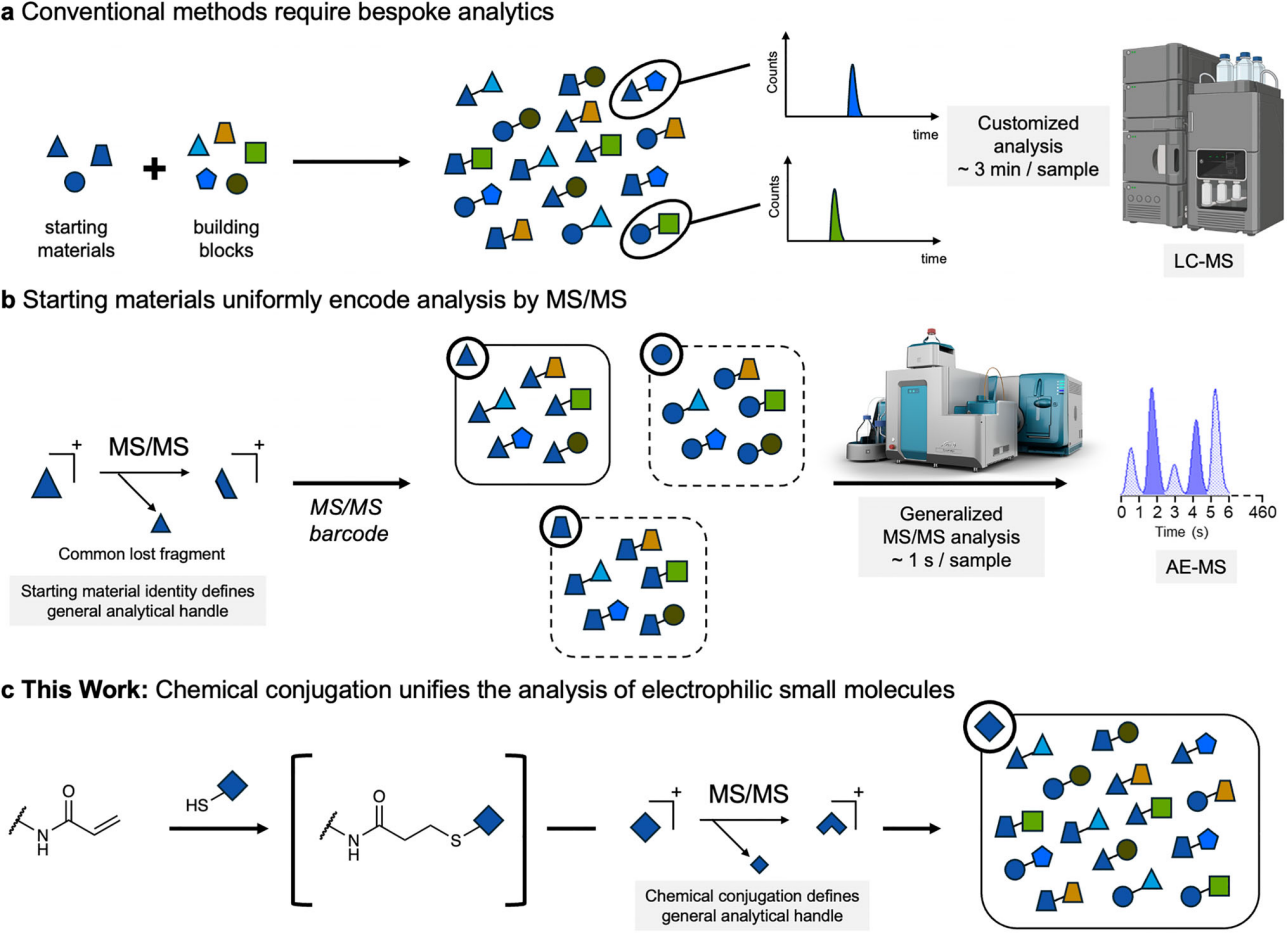
Fragmentation‐first experimentation streamlines high‐throughput synthesis. (a) Typical methods for chemical reaction analysis hinge on liquid chromatography mass spectrometry (LC‐MS) data which operate at a pace of 3 min per sample and require curation of retention times to yield accurate data. (b) Starting materials are the common components which feature in every synthetic route. Simple MS/MS analysis of starting materials transforms the fundamental feature of every chemical reaction into a generalized method for analyzing the outputs of chemical reactions. When combined with acoustic ejection mass spectrometry (AE‐MS) this allows reaction outcomes to be determined at a pace of ~1 s per sample. (c) Electrophilic sites might serve another type of general feature to streamline analysis. Here, chemical conjugation to a thiol‐probe provides a uniform analytical readout by MS/MS, and instead of starting material identity defining analytics, a simple chemical property unifies analysis across different types of starting materials.

Tandem mass spectrometry (MS/MS) offers untapped potential for accurate reaction scouting because it can read out reaction components directly from complex mixtures [27]. Yet conventional MS/MS deployment for chemical synthesis has been limited by labor‐intensive, analyte‐specific method development: choosing transitions and collision energies that must be re‐optimized as substrates change, undermining scalability and generality. To overcome this, we recently developed a fragmentation‐first experimentation workflow [28, 29, 30] that inverts the usual target‐first paradigm (Figure 1b). Rather than tailoring assays to each expected product, we exploited neutral loss MS/MS centered on common lost fragments obtained directly from starting materials to generate diagnostic signals that unify analysis across product sets. By anchoring detection to shared fragmentation events, our approach eliminates per‐reaction customization, increases tolerance to co‐elution and ion‐suppression, and enables rapid, plate‐wide analytics without exhaustive chromatographic development. When combined with acoustic ejection mass spectrometry (AE‐MS) samples can be introduced at up‐to 1 s per sample into high levels of dilution limiting the influence of matrix effects which can interfere with many other types of rapid MS/MS analysis. The result is a workflow which enables starting materials to uniformly encode the analysis of their products to streamline synthesis and outputs data of equivalent accuracy as liquid chromatography mass spectrometry (LC‐MS) to rank relative reaction outputs. Development of AE‐MS methods leveraging TOF‐MS has also shown how high‐resolution mass spectrometry can provide rapid assessments of chemical reaction outcomes [31]. MS/MS methods often provide a greater accuracy of product assignment and sensitivity, particularly when complex matrix components are present, however, in cases where competing fragmentation patterns dominate, TOF‐MS methods are more suitable, because new adducts or electrophilic groups can alter fragmentation behavior in unexpected ways [32]. As it stands, neither technique is currently able to achieve standard‐free quantification due to differences in ionization efficiency between analogs. Yet, when the goal is to analyze most molecules the same way, a fragmentation‐first approach captures large fractions of chemical space within a simple and tractable method development regime.

Our prior work showed how fragmentation patterns determined from starting materials could serve as common features for MS/MS method development (Figure 1b). Along these lines, we reasoned that a similar method should be applicable to chemical properties of small molecules; we suspected that electrophilicity could be one such property that might be exploited to generalize chemical product analysis. We envisaged that this could be achieved for electrophilic small molecules by their conjugation with a suitable nucleophile (Figure 1c). In this way, we could overwrite the inherent fragmentation features of a small molecule through its conjugation with a nucleophilic probe, much like how tandem‐mass tags [33] encode proteomics workflows. As complex electrophilic small molecules are most commonly employed as covalent inhibitors of proteins, we initially focused on PD168393, sotorasib, and ibrutinib (Figure 2) to test the capacity of chemical conjugation to streamline electrophilic small molecule analysis by MS/MS. This set of small molecules was highly suitable for the selected approach because they are deliberately designed to be sterically accessible for cysteine targeting. Subjecting these three molecules to product ion scanning MS/MS revealed that each molecule presented a unique dominant fragmentation pattern precluding common fragment‐driven analysis, 56, 152, and 137 Da respectively (Figure 2). Next, we sought to interrogate the conjugation of these molecules with nucleophiles in a miniaturized format. As suitable conjugation agents, we considered common sulfur‐centered nucleophiles Boc‐L‐cysteine (Boc‐L‐cys), L‐cysteine (L‐cys), and glutathione (GSH), each of which would immediately react with these acrylamides. A panel of reaction conditions was explored using super stoichiometric amounts of these thiol probes, and we identified that each probe provided essentially complete conversion (Figure S1a). Product ion scanning mass spectrometry experiments on crude adducts **1–3** revealed that union with any of the three nucleophiles overwrote the parental MS/MS signatures to yield a single profile which was dependent on the thiol selected for conjugation (Table S1). For Boc‐L‐cysteine adducts **1a–c**, the carbamate carbonyl C‐N bond was broken to yield a neutral fragment of 100 Da, for L‐cysteine adducts **2a–c**, the thioether linkage was broken (89 Da), and for GSH adducts **3a–c**, the glutamate amide C‐N bond broke to give a 129 Da fragment. Among these, the loss of the Boc group required the lowest average collision energy (CE) to achieve fragmentation and would therefore yield the most mild and general behavior. Indeed, by using the Boc‐L‐cystine adduct fragmentation signature to develop a neutral loss MS/MS profile allowed all three adducts **1a–c** to be analyzed using the same single method and fragmentation behavior (Figure S1b–d). Moving forward, we utilized Boc‐L‐cysteine as the CE of cleavage of the Boc group upon adduct formation was lowest (Table S2). To avoid erroneous assignment of neutral loss signals within more complex chemical mixtures, target specific masses were directly extracted from the neutral loss scan data. In this way, we are afforded the sensitivity of MS/MS‐based detection as well as the accuracy of the parental molecule identity.

**FIGURE 2:**
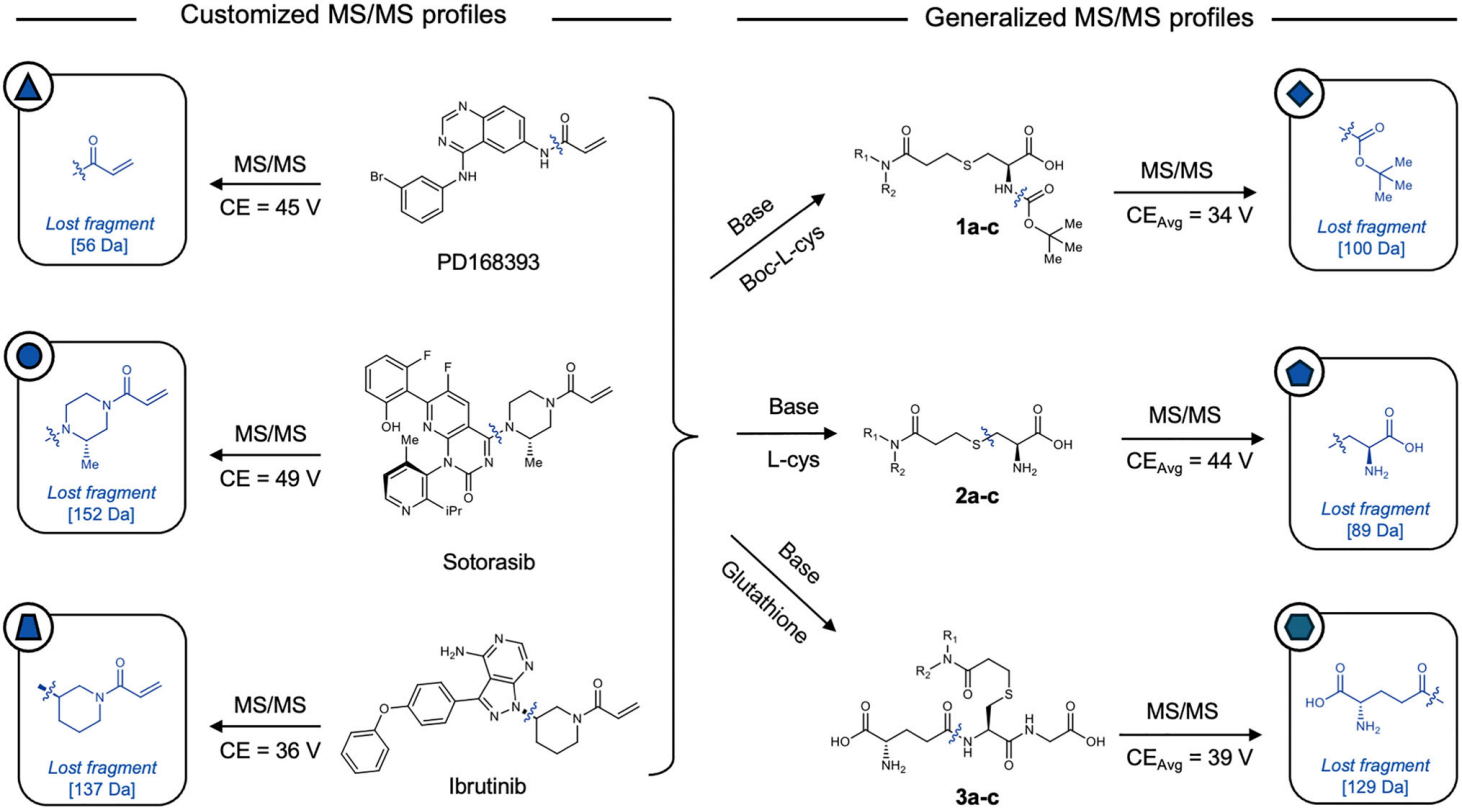
Thiol‐adducts generalize the analysis of electrophilic small molecules. Product ion scanning tandem mass spectrometry analysis of PD168393, sotorasib, and ibrutinib reveals that each has prominent unique fragments. Upon addition of thiols these fragmentation behaviors are rewired to center on the behavior of the thiol adduct, independent of the parent molecules behavior.

To test the broader reach of our approach, we assembled a library of covalent chemical fragments from our in‐house screening library (Figure 3). This consisted of a range of structurally diverse acrylamides featuring secondary and tertiary amides; such broad coverage would allow interrogation of the nature of the amide nitrogen on our analytical robustness. This is because variations at the amide nitrogen are well understood to influence the electrophilic reactivity of acrylamides toward cysteines and other nucleophilic thiols [34]. To establish the range of fragmentation patterns within our set of covalent fragments, we collected product ion scanning MS/MS data. Using the top two most abundant fragmentation channels we found that the minimum number of fragmentation patterns to cover our full set of electrophilic fragments was 83. The most frequent fragmentation patterns were losses of 71 Da (27%) and 54 Da (24%) (Figure 3). Considering their high occurrence, we investigated the potential of this behavior to generalize the analysis of our electrophile library. We performed a neutral loss mass spectrometry experiment searching for lost fragments of either 71 Da or 54 Da across the whole library of electrophilic small molecules. Here, we found that this property alone fell short of being able to analyze most of the library with a neutral loss scanning method able to detect at most 60% of our library with a signal strength of > 20 000 counts by AE‐MS (i.e., sufficient MS response to be able to adequately categorize relative reaction outcomes from high‐throughput chemical synthesis experiments). These data were consistent with the electrophilic handle (the acrylamide) alone not being sufficient to generalize analysis, and that chemical conjugation with a fragmentation‐enabled handle was required.

**FIGURE 3:**
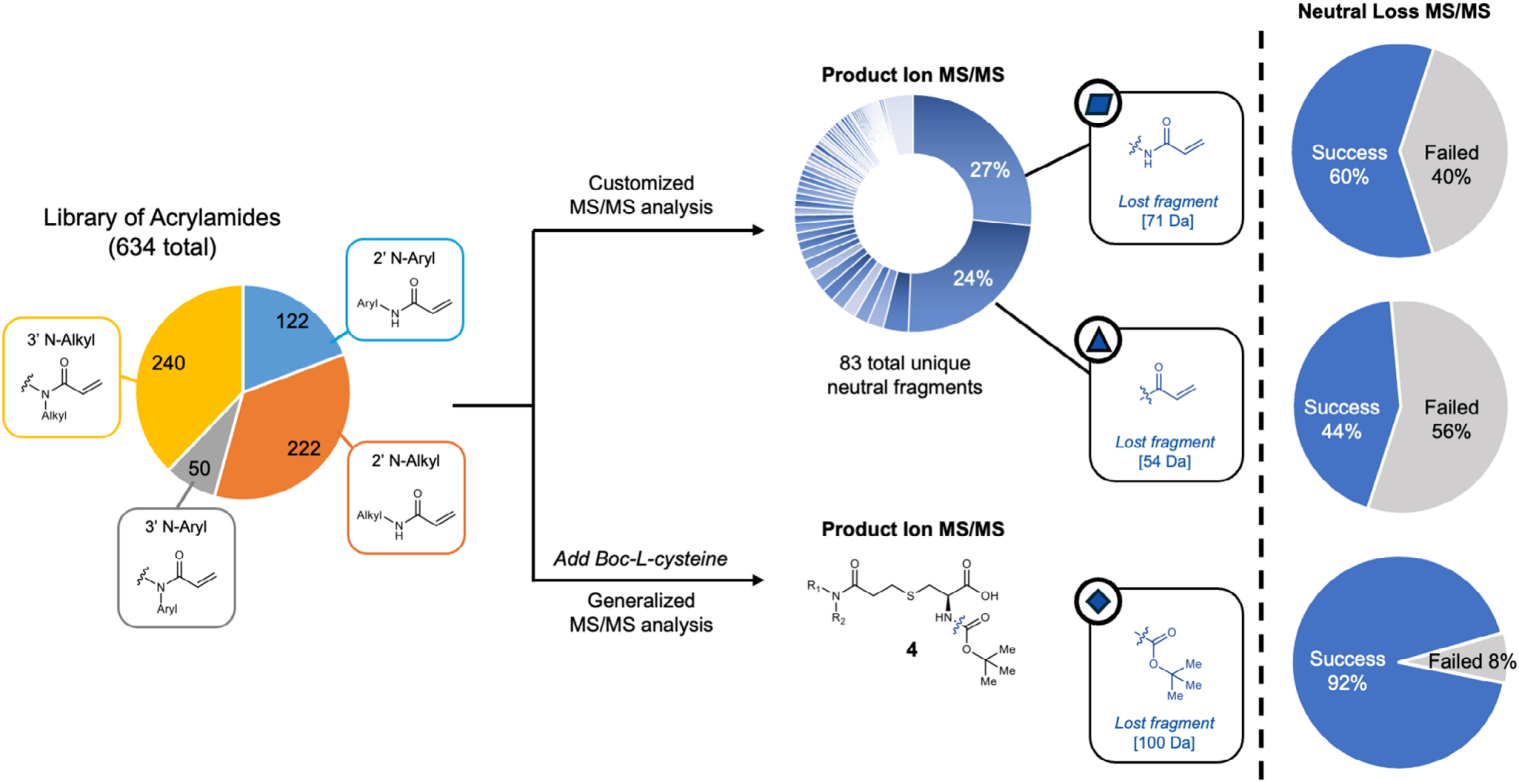
Thiol conjugation generalizes the analysis of diverse acrylamide types. Using product ion scanning MS/MS analysis 83 unique fragmentation signatures were identified for a library of 634 acrylamides. The top two fragmentation patterns were leveraged to action neutral loss MS/MS which revealed that the presence of an acrylamide alone was not sufficient to generalize small molecule analysis. Conjugation of this library to Boc‐L‐cysteine allowed > 90% of the library to be analyzed using a single method demonstrating analytical generality. Cut‐off conditions for a valid neutral loss signals were > 20 000 counts at an expected maximum sample concentration of 25 µM.

Accordingly, we treated the entire library of acrylamides with Boc‐L‐cysteine. Analysis of the products by neutral loss MS/MS showed that chemical conjugation overwrote the inherent fragmentation behavior and allowed > 90% of this library to be analyzed using the single fragmentation pattern derived from conjugation to Boc‐L‐cysteine (Figures 3 and S2). Secondary analysis of the MS/MS data for our acrylamide library revealed a wide range of collision energies and survival yields for the fragmentation of the core common arylamide portion (Figures S3–S6). These data further supported the utility of chemical conjugation for reducing the operational complexity of analyzing electrophilic small molecules. Analysis of failure modes inherent to our method revealed that although thiol adducts could be formed, as detected by LC‐MS, that in‐source fragmentation was a primary contributor to the loss of signal for 38 of the 48 failed analyses due to reduced amounts of parent ion successfully entering the MS/MS system (Figure S2, Table S2).

We next sought to apply our method to the analysis of the products of high‐throughput chemical synthesis and thereby leverage the electrophilic properties of small molecules to generalize their analysis. To create a suitable set of reactions, we assembled microliter‐scale reaction mixtures in 384‐well plates using multichannel pipettes or automated liquid handling [2, 3, 4, 5, 7, 8, 28, 29]. A Buchwald–Hartwig reaction of pyridinyl acrylamide **5** with six aryl amines (**7**–**12**) was performed using four solvents (DMSO, NMP, DMAc, and DMF), four bases (DBU, BTMG, MTBD, and P2Et), and four palladium catalysts (cataCXium Pd G4, tBuXPhos Pd G4, tBuBrettPhos Pd G4, and XantPhos Pd G4) (Figures 4b and S7). Collectively, these conditions spanned 384 unique reactions. Following incubation for 20 h at room temperature, an aliquot of each reaction mixture was treated with Boc‐L‐cysteine and DBU. The Boc‐L‐cysteine adducts of all products were analyzed using a single method by neutral loss MS/MS in just 19.2 min. A second aliquot was taken from the reaction plate and analyzed via LC‐MS over 19 h. Despite measuring different analytes between LC‐MS and AE‐MS, point‐to‐point responses were in excellent agreement for ranking relative reaction outputs (Figure 4b, *R* ^2^ = 0.91). Taking these conditions in aggregate allows the assessment of performance of the 64 unique reaction conditions across the entire set of six target products. Accordingly, the best performing conditions (tBuXPhos Pd G4, P2Et) were accurately selected by our MS/MS driven method (Figure 4b).

**FIGURE 4:**
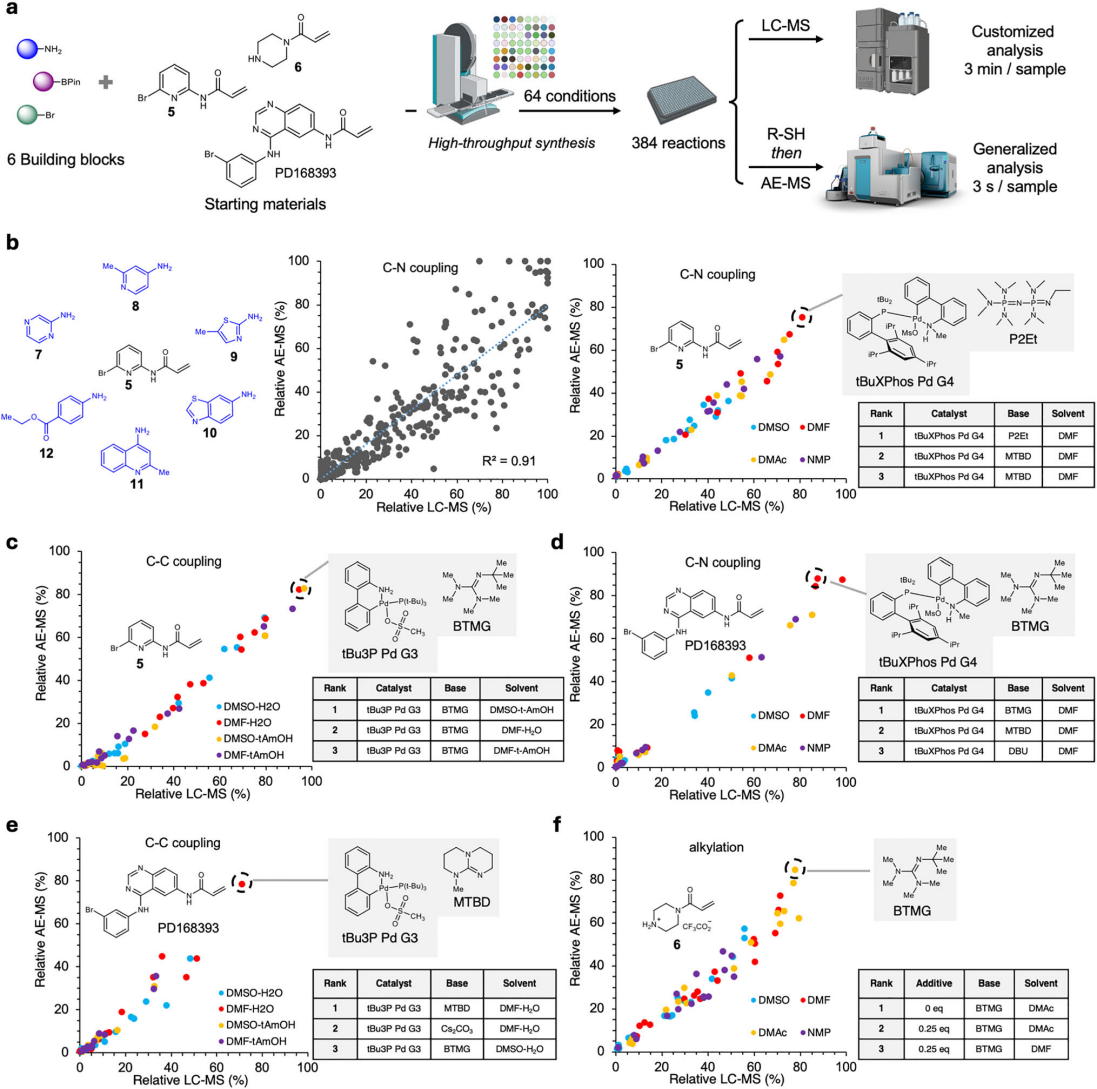
Generalized analysis of small molecules from high‐throughput experimentation. (a) Experiments were performed in 384‐well plate formats. An aliquot was analyzed by conventional LC‐MS, and a second aliquot was treated with Boc‐L‐cysteine/DBU then analyzed by AE‐MS. (b) Point to point data were compared for the Buchwald–Hartwig C─N coupling reaction of pyridinyl acrylamide **5** which showed excellent agreement between LC‐MS and AE‐MS data. Averaging outputs allowed individual conditions to be assessed. Additional types of reactions were investigated by this approach: (c) Suzuki–Miyaura C─C coupling reaction of pyridinyl acrylamide **5**; (d) Buchwald–Hartwig C─N coupling reaction of PD168393; (e) Suzuki–Miyaura C─C coupling reaction of PD168393; and (f) N‐alkylation of piperazinyl acrylamide **6**.

A series of additional reactions were explored to establish the generality of our approach. A Suzuki–Miyaura cross coupling reaction was performed using five and six pinacol boronic ester coupling partners (**13**–**18**, Figures 4c and S8). Here the best performing catalysts were PtBu3 Pd G3 and bartons base (BTMG) with DMSO/t‐AmOH as the solvent. Switching to a more complex aryl halide coupling partner PD168393, we performed the same panel of Buchwald–Hartwig and Suzuki–Miyaura cross coupling reactions (Figures 4d,e, S8 and S9). In this instance, PD168393 was much more sensitive to the choice of reaction conditions, with a large number of reactions failing to produce product (Figure S9). Nevertheless, tBuXPhos Pd G4 and bartons base (BTMG) in DMF proved to be the optimal conditions for performing C─N coupling. Several other conditions were comparable but featured only exchange of the base, with Pd tBuXPhos G4 being the standout best catalyst (Figure 4d). Similarly, the Suzuki–Miayura cross coupling reaction with PD168393 displayed marked sensitivity to reaction condition choice with PtBu3 Pd G3 being the standout catalyst system explicitly in DMF/H_2_O and using MTBD as the base—these conditions provided close to double the product output than all others tested (Figure S10). Finally, we performed an N‐alkylation reaction of piperazinyl acrylamide **6** with six alkyl halides (**19**–**24**, Figures 4f and S11), four solvents (DMSO, NMP, DMAc, DMF), six alkyl halides, eight bases (DBU, BTMG, MTBD, P2Et, 2,6‐Lut, DIPEA, KOtBu, and KOTMS), as well as a binary additive condition state (25 mol% tetrabutylammonium iodide or none). Here, the best performing conditions (BTMG in DMAc, with no additive) were accurately selected by our MS/MS‐driven method. Across all tested sets of compounds, the performance of our thiol‐quenched reactions by AE‐MS was in excellent agreement with the corresponding LC‐MS data, demonstrating that the reaction matrix does not impact our capacity to rank order reaction outcomes. When applied to synthesis at large, it is important to consider that ionization efficiency will vary across different products, which in some cases, might lead to difficulties in accurate reaction outcome prediction.

Functional electrophiles for engaging cysteines come in many different forms, so we considered that our approach for rewiring MS/MS behavior should apply to other classes of electrophile apart from just acrylamides. To demonstrate this concept, we employed a series of small molecules featuring cysteine reactive electrophiles (**25**–**35**), each featuring distinct fragmentation behaviors (Figure 5). Initial attempts using Boc‐L‐cys were unsuccessful; however, by simply switching to thiophenol‐based substrate **36**, a wide range of electrophilic small molecules could be reprogrammed, through conjugation (**25a**–**35a**), to a single MS/MS signature (Figures 5 and S12).

**FIGURE 5:**
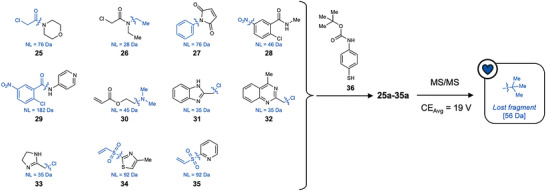
Rewiring electrophile analysis. Addition of thiol **36** to a diverse clutch of electrophilic small molecules allows direct analysis via a single MS/MS fragmentation channel. NL = Neutral Loss fragment upon MS/MS analysis.

In conclusion, ranking chemical reaction outputs stands as one of the major rate‐limiters for leveraging data‐driven tools at the frontiers of synthetic thinking. Efficient development of MS/MS methods will unlock this functional potential. While previous methods have leveraged starting material identity to generalize the analysis of small molecules, here we have taken a fundamental property of a small molecule, its electrophilicity, and transformed that into a vector for generalizing analysis through chemical conjugation. This type of fragmentation‐first thinking will reshape overarching strategies employed to collect high‐throughput reaction data. Moreover, given the growing prevalence of covalent inhibition as a drug design modality the approach we detail will be of broad utility in the design of electrophilic small molecules for direct‐to‐biology applications [35] and ranking their relative reaction outcomes.

## Author Contributions

**Uma Neelakantan**: methodology, data curation, investigation, validation, formal analysis, visualization, writing – review and editing, writing – original draft. **Maowei Hu**: methodology, data curation, formal analysis, visualization, investigation, supervision, writing – review and editing, writing – original draft. **Jiya Bhatia**: methodology, formal analysis, investigation, data curation. **Huyen N. Nguyen**: methodology, data curation, formal analysis. **John (Bobby) Yeboah**: methodology, data curation. **Eirinaios I. Vrettos**: methodology, data curation. **Dionysius Copoulos**: investigation, methodology, data curation. **Khue N. M. Nguyen**: methodology, investigation, data curation. **Madan M. Babu**: supervision. **Daniel J. Blair**: supervision, conceptualization, funding acquisition, writing – original draft, writing – review and editing, visualization, project administration, resources, formal analysis, data curation.

## Conflicts of Interest

The authors declare no conflicts of interest.

## Supporting information



The authors have cited additional references within the Supporting Information [36].
**Supporting File**: anie72797‐sup‐0001‐SuppMat.docx.

## Data Availability

The data that support the findings of this study are available in the Supporting Information of this article.
