# Supplementary material for: Generalized Analysis of Electrophilic Small Molecules
Source: Angew Chem Int Ed Engl. 2026 May 25;65(28):e8895173. doi: 10.1002/anie.8895173 (PMC13340528; doi:10.1002/anie.8895173)

Supporting Information for

Generalized Analysis of Electrophilic Small Molecules

Uma Neelakantan,^[a][b]^ Maowei Hu,^[a]^ Jiya Bhatia,^[a]^ Huyen (Scarlett) Nguyen,^[a][c]^ John (Bobby) Yeboah,^[a][c]^ Eirinaios I. Vrettos,^[a]^ Dionysius Copoulos^[a]^, Khue N. H. Nguyen^[a]^, M. Madan Babu,^[b]^ and Daniel J. Blair*^[a][c]^

[a] Uma Neelakantan, Maowei Hu, Jiya Bhatia, Huyen (Scarlett) Nguyen, John (Bobby) Yeboah, Eirinaios I. Vrettos, Dionysius Copoulos, Khue Nguyen, Daniel J. Blair
Department of Chemical Biology and Therapeutics
St Jude Children’s Research Hospital
262 Danny Thomas Place, Memphis, TN, 38105
E-mail: [Daniel.Blair@stjude.org](mailto:Daniel.Blair@stjude.org)

[b] Uma Neelakantan, M. Madan Babu

Center of Excellence for Data Driven Discovery

Department of Structural Biology

St Jude Children’s Research Hospital

262 Danny Thomas Place, Memphis, TN, 38105

[c] Huyen (Scarlett) Nguyen, John (Bobby) Yeboah, Daniel J. Blair
Department of Pharmaceutical Sciences,
College of Pharmacy, University of Tennessee Health Science Center
Memphis TN 38163, USA

* Email: daniel.blair@stjude.org

Contents

[1. Supplementary Figures 3](#_Toc227963650)

[Figure S1. Optimization of thiol adduct formation with model substrates PD16893, sotorasib, and ibrutinib. 3](#_Toc227963651)

[Figure S2. Distribution of compounds that have neutral loss signal below 20000 from the library. 3](#_Toc227963652)

[Figure S3. Distribution of collision energy (CE) for 634 covalent library compounds. 4](#_Toc227963653)

[Figure S4. Distribution of parent signal intensity (Q1) for 634 covalent library compounds 4](#_Toc227963654)

[Figure S5. Distribution of daughter signal intensity (Q3) for 634 covalent library compounds 5](#_Toc227963655)

[Figure S6. Calculated % signal (Q3/Q1) for 634 covalent library compounds 5](#_Toc227963656)

[Figure S7. Pyridinyl acrylamide 5 Buchwald-Hartwig C-N coupling reaction. 6](#_Toc227963657)

[Figure S8. Pyridinyl acrylamide 5 Suzuki–Miyaura C-C coupling reaction. 7](#_Toc227963658)

[Figure S9. PD168393 Buchwald-Hartwig C-N coupling reaction. 8](#_Toc227963659)

[Figure S10. PD168393 Suzuki–Miyaura C-C coupling reaction 9](#_Toc227963660)

[Figure S11. Piperazinyl acrylamide 6 *N*-alkylation reaction 10](#_Toc227963661)

[Figure S12. AE-MS to analyze 28a-35a by the common loss of a 56 Da fragment 11](#_Toc227963662)

[2. Materials and General Methods 12](#_Toc227963663)

[2.1 Reagents and solvents 12](#_Toc227963664)

[2.2 Product ion scan 12](#_Toc227963665)

[2.3 Acoustic droplet ejection-mass spectrometry (AE-MS) 12](#_Toc227963666)

[Table S1 Neutral loss data from product ion scanning 13](#_Toc227963667)

[Table S2 LC-MS and MS/MS analysis of failed Boc-L-cysteine adducts 15](#_Toc227963668)

[Table S3 AE-MS parameters 16](#_Toc227963669)

[2.4 Liquid chromatography-mass spectrometry (LC-MS) 16](#_Toc227963670)

[2.5 Probe quench condition optimization 17](#_Toc227963671)

[3. Synthesis and Plate Reactions 17](#_Toc227963672)

[3.1 Synthesis of pyridinyl acrylamide 5 17](#_Toc227963673)

[3.2 Pyridinyl acrylamide 5 Buchwald-Hartwig C-N coupling reaction 19](#_Toc227963674)

[3.3 Pyridinyl acrylamide 5 Suzuki–Miyaura C-C coupling reaction 21](#_Toc227963675)

[3.4 PD168393 Buchwald-Hartwig C-N coupling reaction 23](#_Toc227963676)

[3.5 PD168393 Suzuki–Miyaura C-C coupling reaction 25](#_Toc227963677)

[3.6 Piperazinyl acrylamide 6 *N*-alkylation reaction 27](#_Toc227963678)

[4. CID Spectra 29](#_Toc227963679)

[5. References 68](#_Toc227963680)

[6. NMR Spectra 69](#_Toc227963681)

# Supplementary Figures


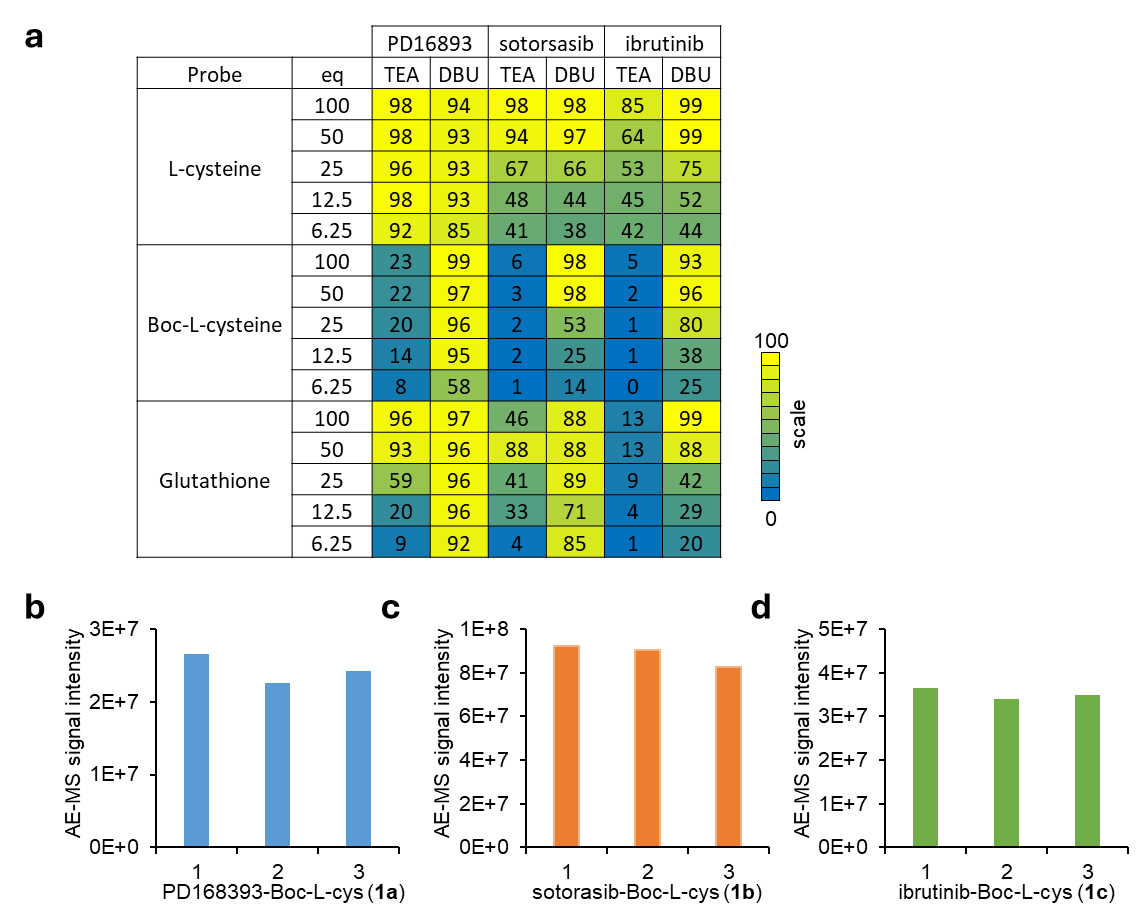


Figure S1. Optimization of thiol adduct formation with model substrates PD16893, sotorasib, and ibrutinib. a) A range of probe and base equivalents were investigated to achieve complete conversion of acrylamides to their thiol adducts. Conversion shown as % was derived from the following equation conv% = thiol adduct MS signal/(thiol adduct MS signal + starting material MS signal) as measured by LC-MS (125 µM). Using the MS/MS profiles of Boc-L-cysteine (Boc-L-cys) adducts 1a-c, a single neutral loss scanning method was created. This method was used in combination with AE-MS to analyze b) PD168393-Boc-L-cys (1a), c) sotorasib-Boc-L-cys (1b), and d) ibrutinib-Boc-L-cys (1c) by the common loss of a 100 Da fragment (12.5 µM).


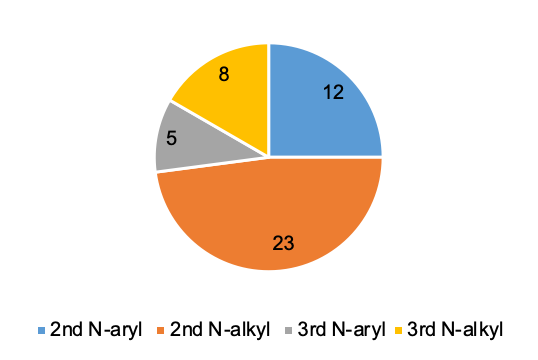


### Figure S2. Distribution of compounds that have neutral loss signal below 20000 from the library.


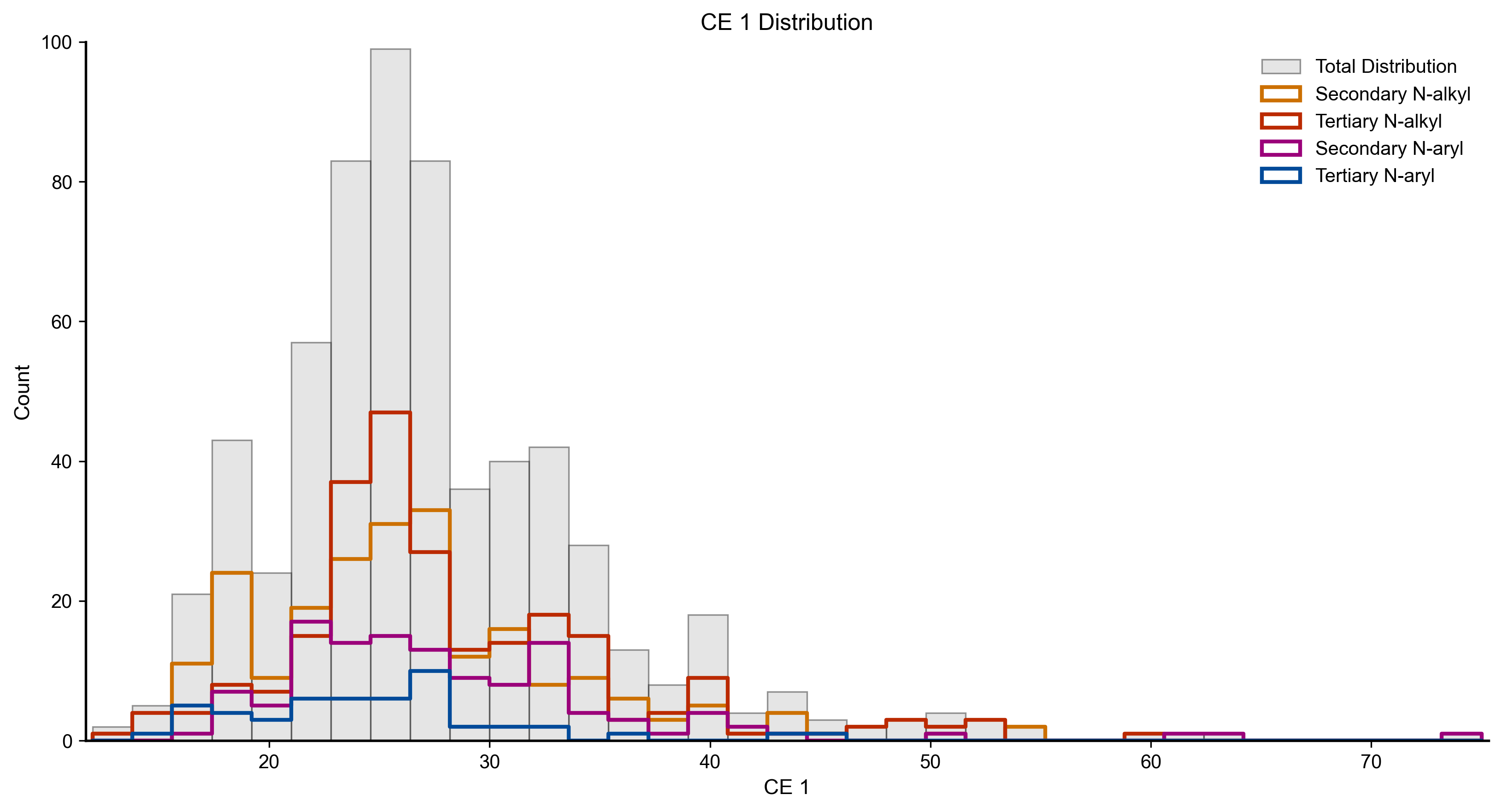


### **Figure S3**. Distribution of collision energy (CE) for 634 covalent library compounds.


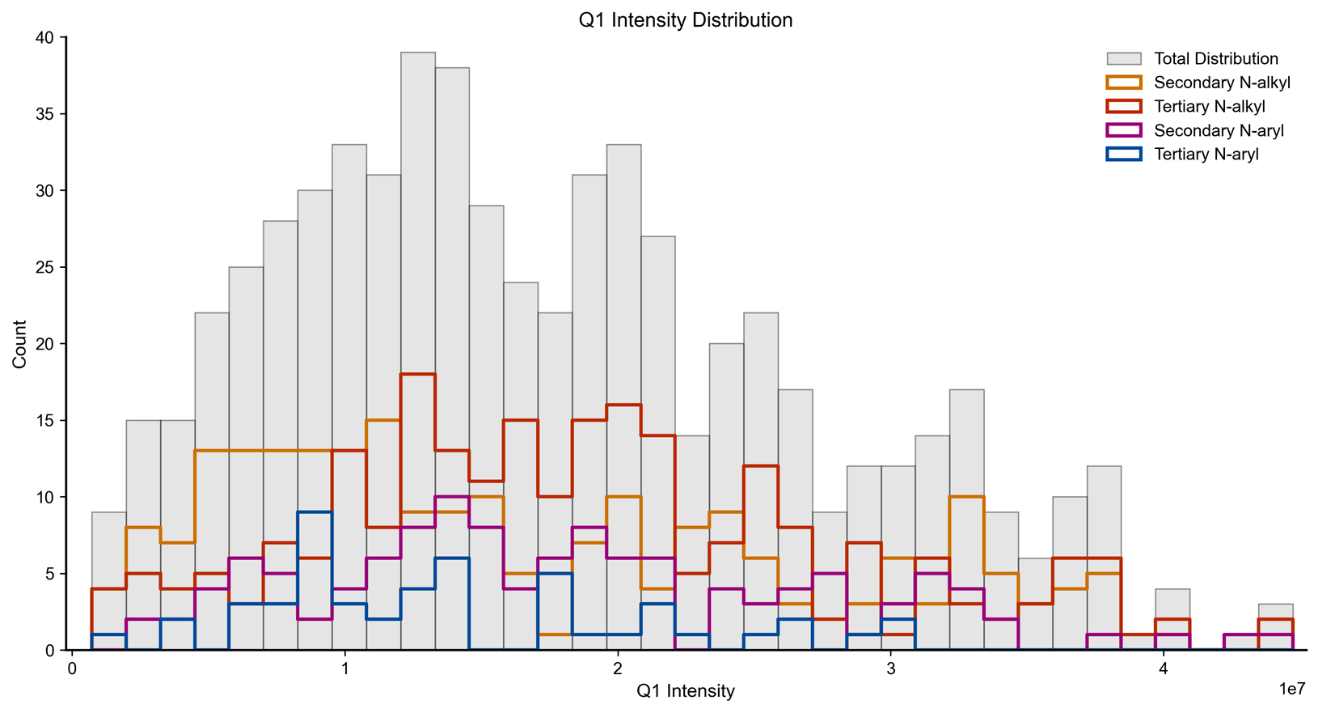


### **Figure S4**. Distribution of parent signal intensity (Q1) for 634 covalent library compounds


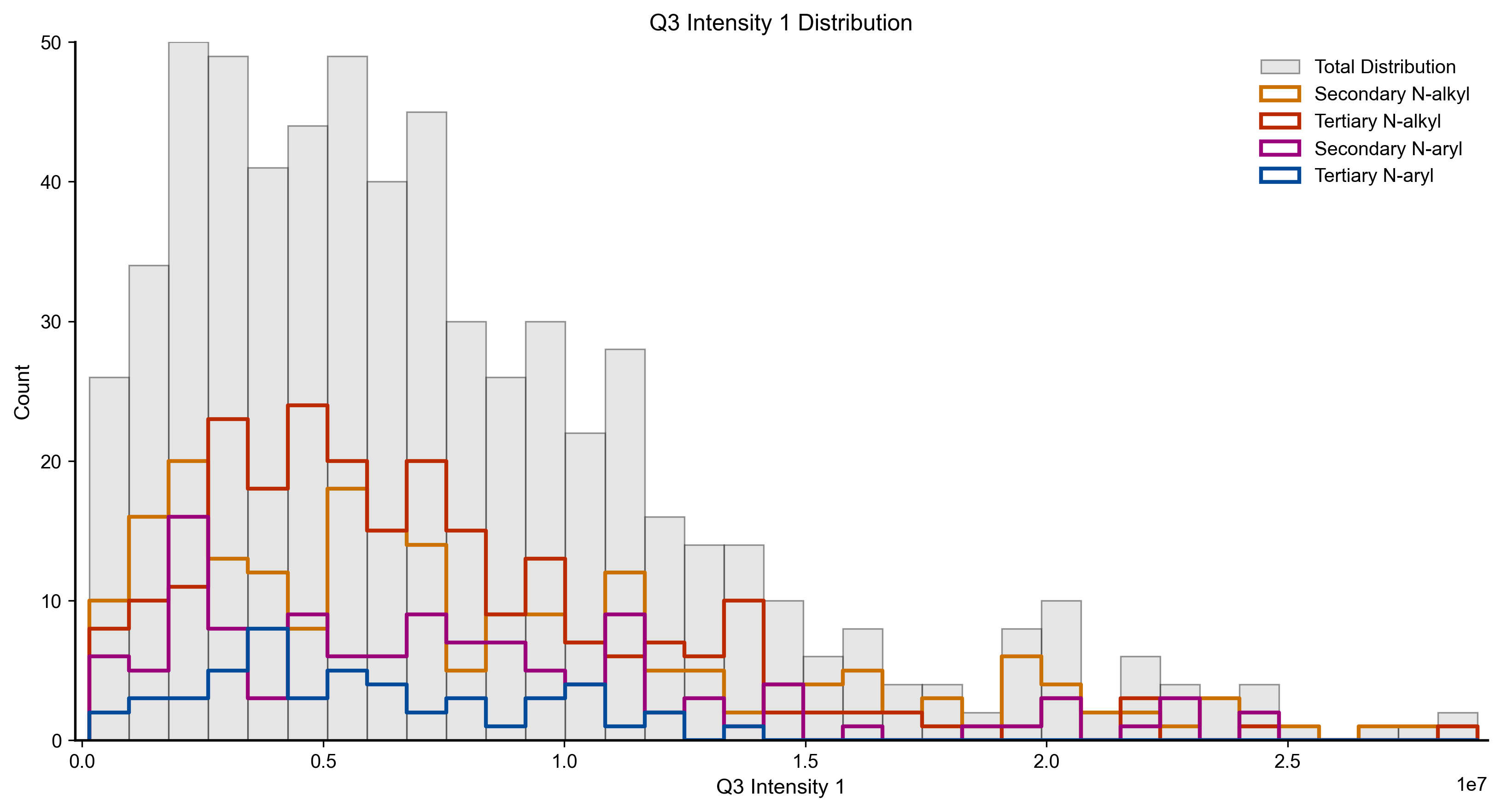


### **Figure S5**. Distribution of daughter signal intensity (Q3) for 634 covalent library compounds


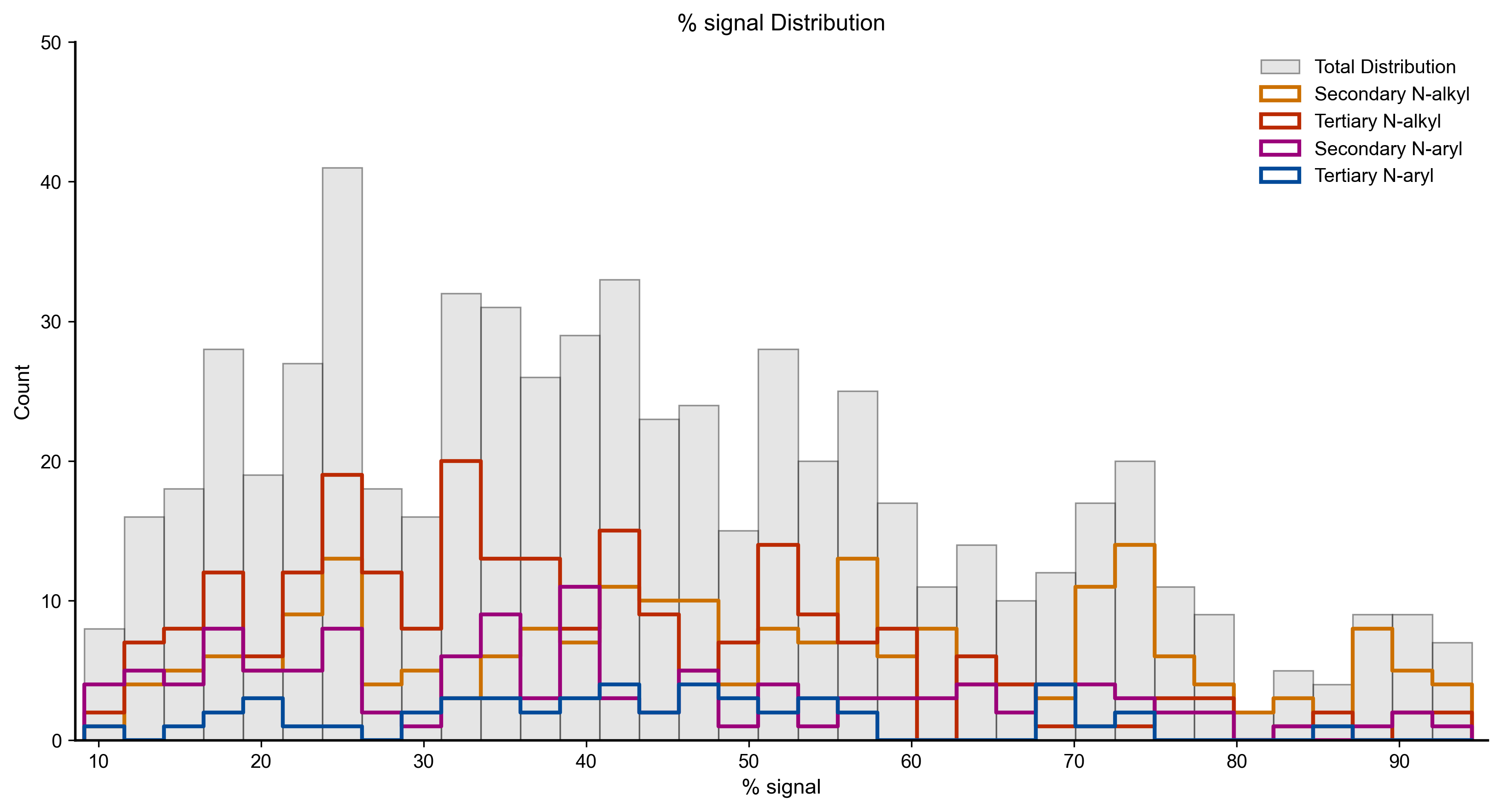


### **Figure S6.** Calculated % signal (Q3/Q1) for 634 covalent library compounds


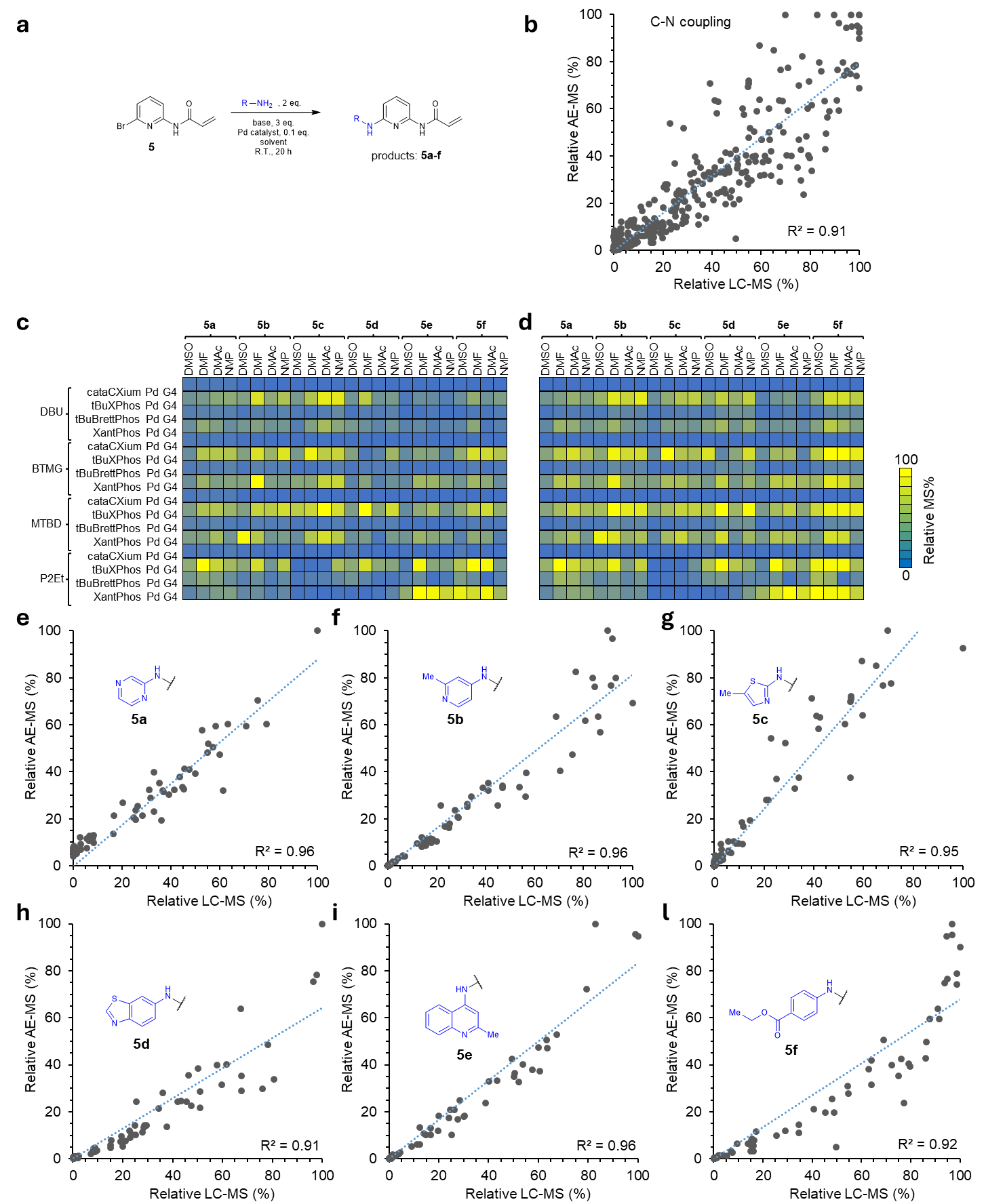


Figure S7. Pyridinyl acrylamide 5 Buchwald-Hartwig C-N coupling reaction. a) Reaction scheme. b) 384 point-to-point comparison. Heat map of c) AE-MS and d) LC-MS. Point-to-point results of product e) **5a**, f) **5b**, g) **5c**, h) **5d**, i) **5e**, and j) **5f**.


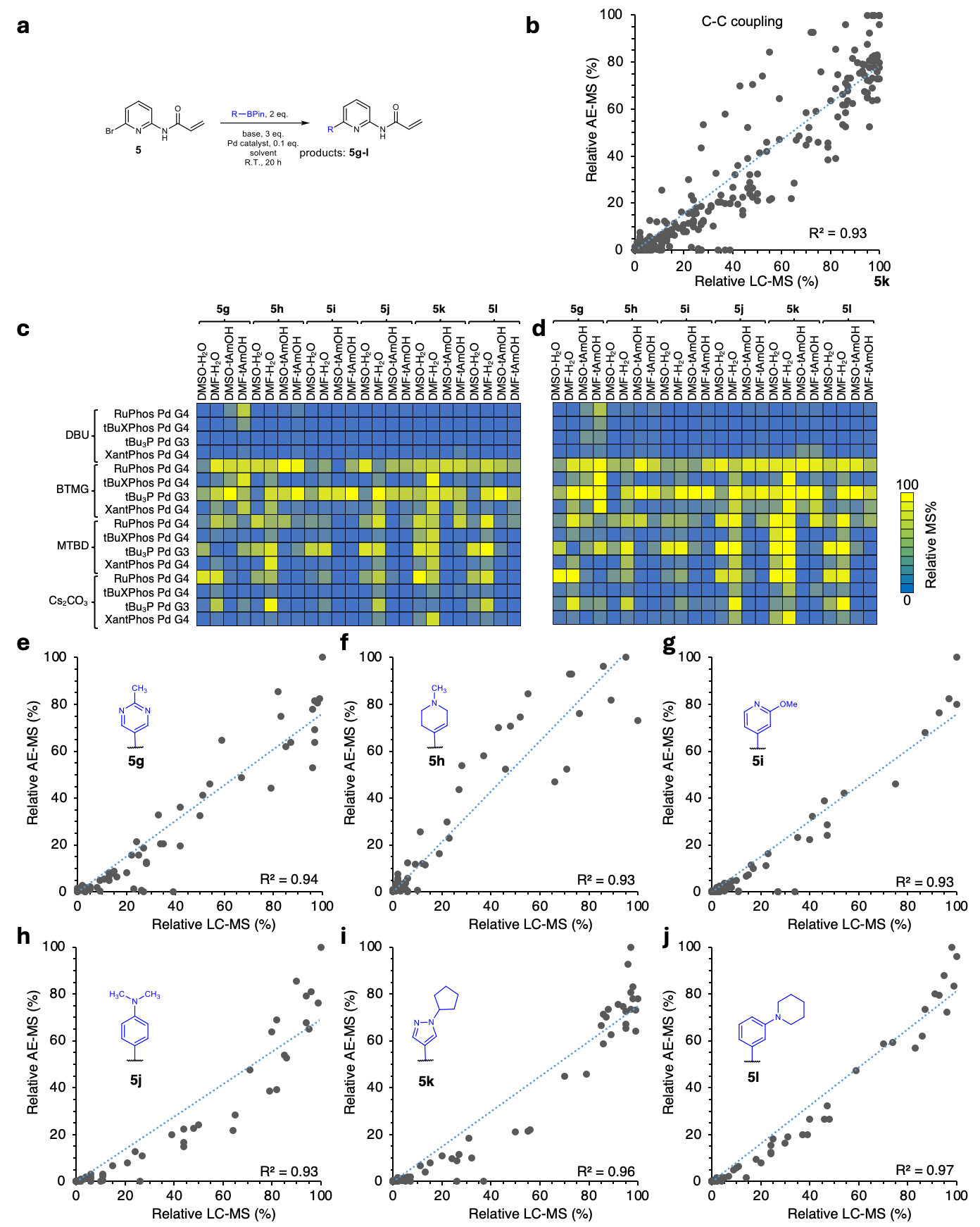


Figure S8. Pyridinyl acrylamide 5 Suzuki–Miyaura C-C coupling reaction. a) Reaction scheme. b) 384 point-to-point comparison. Heat map of c) AE-MS and d) LC-MS. Point-to-point results of product e) **5g**, f) **5h**, g) **5i**, h) **5j**, i) **5k**, and j**) 5l**.

**
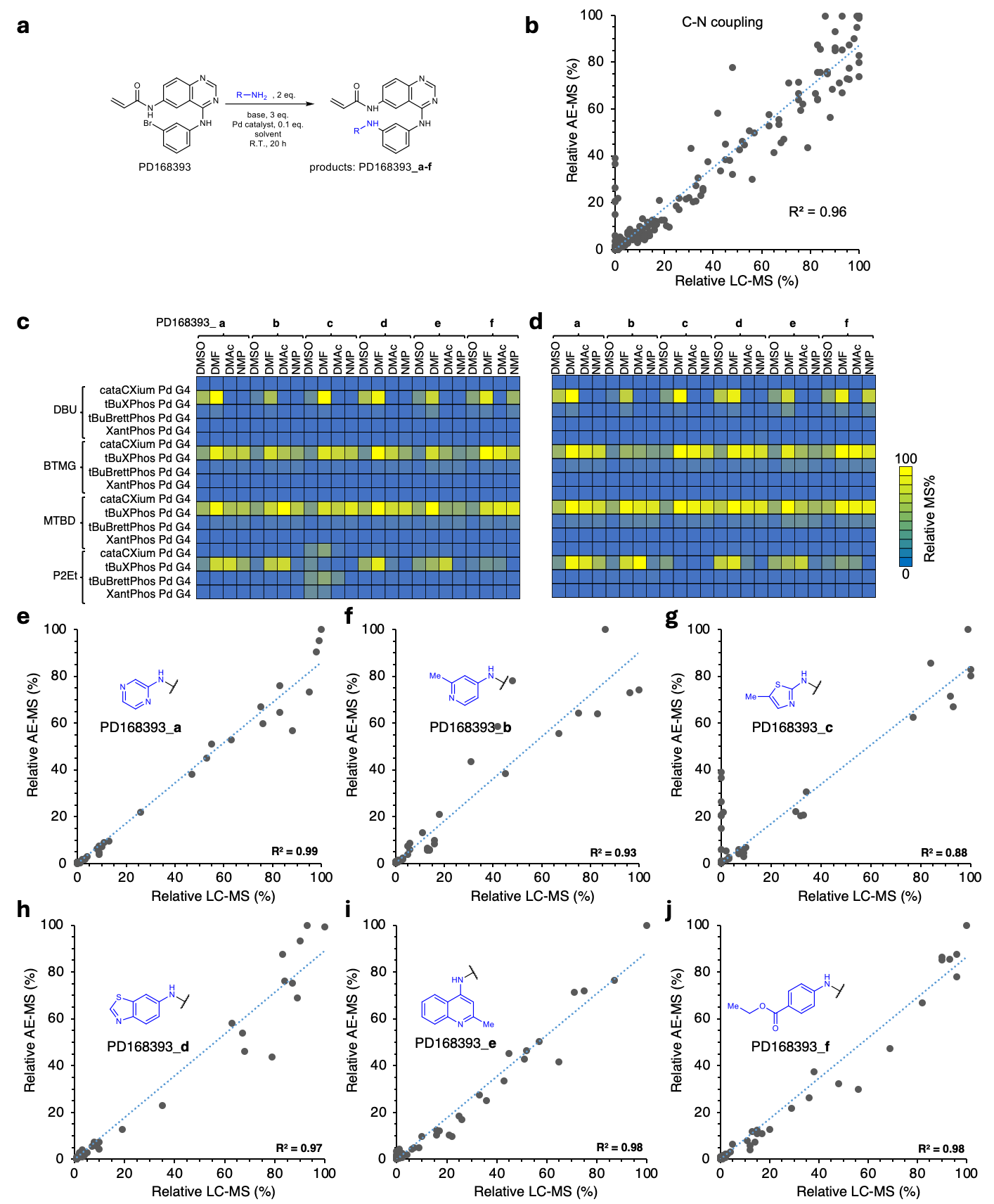
**

Figure S9. PD168393 Buchwald-Hartwig C-N coupling reaction. a) Reaction scheme. b) 384 point-to-point comparisons. Heat map of c) AE-MS and d) LC-MS. Point-to-point results of product e) PD168393_**a**, f) PD168393_**b**, g) PD168393_**c**, h) PD168393_**d**, i) PD168393_**e**, and j) PD168393_**f**.


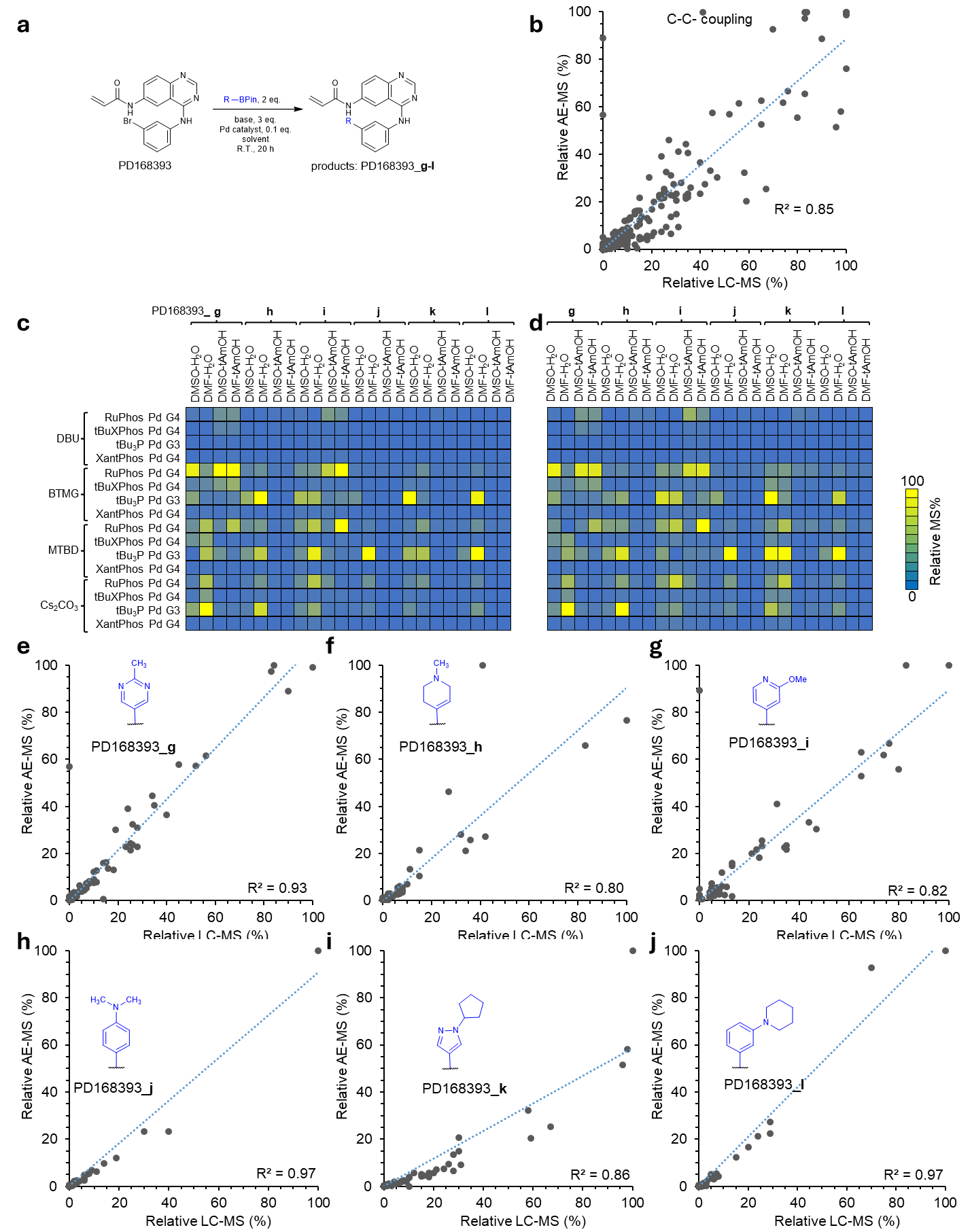


Figure S10. PD168393 Suzuki–Miyaura C-C coupling reaction**.** a**)** Reaction scheme. b) 384 point-to-point comparisons. Heat map of c) AE-MS and d) LC-MS. Point-to-point results of product e) PD168393_**g**, f) PD168393_**h**, g) PD168393_**i**, h) PD168393_**j**, i) PD168393_**k**, and j) PD168393_**l**.


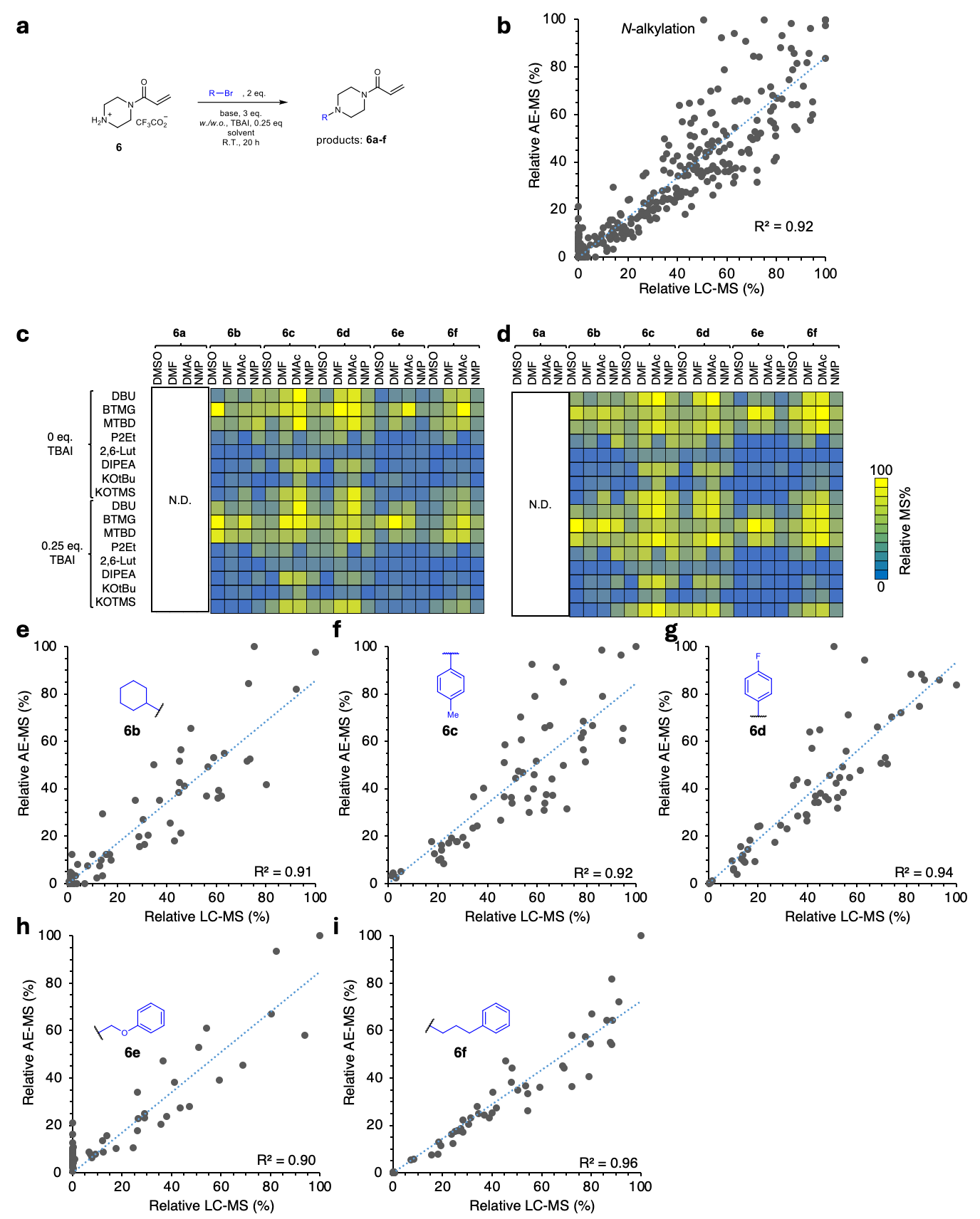


Figure S11. Piperazinyl acrylamide 6 *N*-alkylation reaction. a) Reaction scheme. b) 384 point-to-point comparison. c) Heat map of AE-MS (left) and LC-MS (right). Point-to-point results of product d) **6a**, e) **6b**, f) **6c**, g) **6d**, h) **6e**, and i) **6f**.


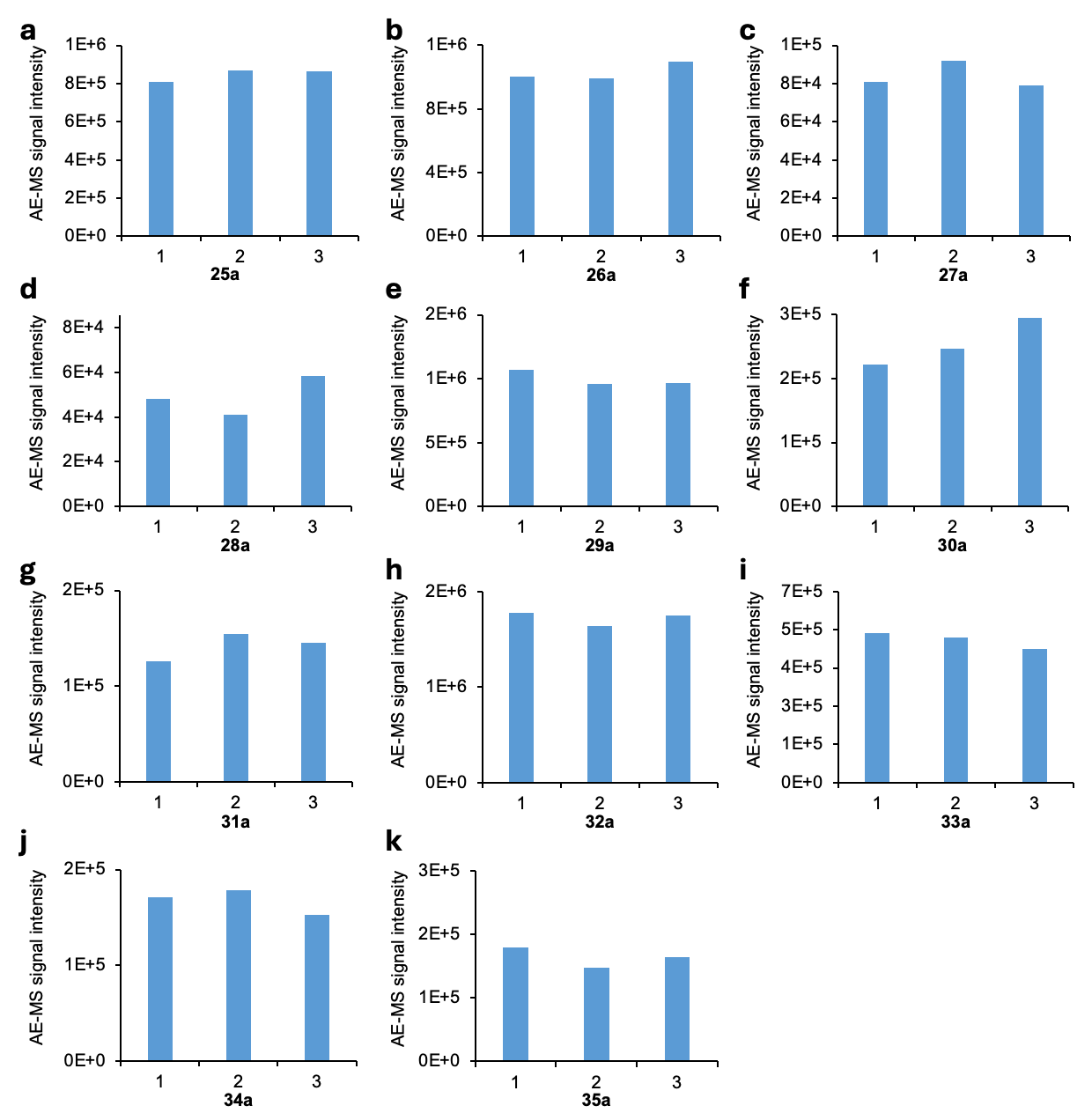


Figure S12. AE-MS to analyze 28a-35a by the common loss of a 56 Da fragment**.**

# 2. Materials and General Methods

## 2.1 Reagents and solvents

Air- and moisture-sensitive reactions were carried out in a nitrogen filled glovebox. Anhydrous Dimethylformamide (DMF) was purchased from Sigma Aldrich and stored in the glovebox. Dimethyl sulfoxide (DMSO) was purchased from Fisher Chemical in 4 L (ACS grade) bottles. Building blocks were selected from St. Jude in house chemical collection and used directly as received from a variety of sources, Oakwood, Acros Organics, Alfa Aesar, Frontier Scientific, Combi-Blocks, Enamine, Matrix Scientific, and Sigma Aldrich. Acetonitrile (LC-MS grade) were acquired from Fisher Scientific (Waltham, MA). Milli-Q water (Millipore, Molsheim Cedex, France) was used as produced. LC-MS grade formic acid (for mass spectrometry) was purchased from Honeywell. ^1^H and ^13^C Nuclear Magnetic Resonance (NMR) spectra were recorded in CDCl_3_ solvent on Bruker Ascend 500 with Avance III HD Console (at 500 MHz and 126 MHz). Chemical shifts (δ_H_ , δ_C_) are calibrated to the residual protio solvent signals of CDCl_3_ (7.26 ppm, 77.2 ppm) and DMSO-d6 (2.50 ppm, 39.5 ppm). Coupling constants are quoted in parts per million (ppm) and refer to apparent multiplicities. Data are listed as follows: chemical shift, multiplicity (s = singlet, d = doublet, t = triplet, q = quartet, quin =quintet, sext = sextet, sept = septet, m = multiplet, br = broad peak, dd = doublet of doublet, etc.), coupling constant, integration, and assignment.

## 2.2 Product ion scan

Product ion scanning MS/MS data for each compound were collected using a SCIEX ExionLC/Qtrap 6500 plus triple-quadrupole mass spectrometer system from SCIEX (Forster City, CA), equipped with an electrospray ion (Turbo Ionspray) interface. The SCIEX ExionLC featured two pumps, an integrated degasser, column oven, autosampler, and sample organizer. Nitrogen served as curtain, nebulizer, and collision gas. Analytes were dissolved individually in 50% acetonitrile aqueous solution and were introduced into the ion source of the mass spectrometer by an autosampler. The following solvent series of was used to introduce the sample and wash the system: The isocratic program had a run time of 2 minutes at 25% A (0.1% formic acid in Milli-Q H_2_O) and 75% B (0.1% formic acid in acetonitrile). The flow rate started at 0.4 mL/min, held for 0.3 minutes, transitioned to 0.05 mL/min over 0.01 minutes, changed to 0.03 mL/min over 1.39 minutes, then returned to 0.5 mL/min over 0.01 minutes, and held for 0.29 minutes. MRM transitions and compound-dependent parameters (including declustering potential (DP), entrance potential (EP), collision energy (CE), and collision cell exit potential (CXP)) for each tested compound were selected using Discovery Quant Software (version 3.0.2). The temperature was set at 300 °C, employing high collision gas, and using a 30 µL injection volume. Two or three of the most abundant fragments of the parent ion were documented for drug compounds, their thiol adducts, and for the covalent library compounds. Scan step size was 0.1 amu, with Q3 scan at 200 amu/s. Compounds were acquired in positive mode, and negative mode if required. Data acquisition was performed with Analyst 1.7.3 (SCIEX).

## 2.3 Acoustic droplet ejection-mass spectrometry (AE-MS)

Samples were prepared in 384-well PP plates (001-14615) with 50 µL of DMSO/H_2_O (*v:v* = 70:30). Acoustic transfer of solutions from 384-well LDV echo plates (001-12782) was the typical method used to generate these samples from quenched reaction mixtures (12.5 mM). Prior to loading into the AE-MS system, the source microplates containing the sample cosolvent underwent centrifugation (5 minutes at 2000 rpm) to eliminate gas bubbles and ensure a uniform fluid meniscus shape. Sample collection was facilitated using the Sciex OS-MQ Analytics Software (version 2.1.6.59781) from Sciex (Forster City, CA), to control an AE-MS system operating in neutral loss mode. This system comprised an externalized transducer assembly from an AE-MS autosampler, an open port interface (OPI) connected to a carrier solvent pump, and a transfer capillary leading to a standard IonDrive Turbo V ESI source of an AB Sciex Triple Quad 6500+ system. The carrier liquid utilized was methanol supplemented with 1 mM ammonium fluoride in methanol, flowing at a rate of 350 µL/min, generating a stable vortex at the OPI inlet for optimized signal performance. Contactless sampling involved ejecting of 2.5 nL directly from the microtiter plate wells into the carrier liquid vortex of the OPI, at a frequency of 3 seconds per well. The electrospray ionization (ESI) source of the triple quadrupole MS instrument operated in positive ionization mode, with nebulizer gas (GS1) set to 90 psi, heater gas (GS2) to 70 psi, curtain gas and collision-activated dissociation (CAD) gas to 35 psi and 9 units, respectively. The mass scan range was set according to samples being studied. For analyte measurements, the following MS parameters were employed: ion source temperature: 500 ˚C; spray voltage: 5500 V; pause time: 5 ms; Q1 operated at unit resolution. To simplify testing, neutral loss AE-MS methods were set up based on the results from multiple reaction monitoring of the model compounds as shown in **Table S1** and **Table S2** (individual) and **Table S3** (average), inclusive of declustering potential (DP), entrance potential (EP), collision energy (CE), and collision cell exit potential (CXP). After acquiring sample batches, data processing was conducted using an in-house-built R script we previously described^28^.

### Table S1 Neutral loss data from product ion scanning

| Molecule | NL (Da) | CE (V) | DP (V) | EP (V) | CXP |
| --- | --- | --- | --- | --- | --- |
|  | 56 | 54 | 120 | 10 | 21 |
|  | 152^a^ | 49 | 140 | 10 | 9 |
|  | 137 | 43 | 110 | 10 | 20 |
| PD16893-L-cys (**1a**) | 89 | 45 | 110 | 10 | 13 |
| Sotorasib-L-cys (**1b**) | 89 | 49 | 170 | 10 | 10 |
| Ibrutinib-L-cys (**1c**) | 89 | 40 | 120 | 10 | 15 |
| PD16893-Boc-L-cys (**2a**) | 100 | 38 | 110 | 10 | 15 |
| Sotorasib-Boc-L-cys (**2b**) | 100 | 35 | 110 | 10 | 13 |
| Ibrutinib-Boc-L-cys (**2c**) | 100 | 30 | 110 | 10 | 17 |
| PD16893-GSH (**3a**) | 129 | 40 | 150 | 10 | 18 |
| Sotorasib-GSH (**3b**) | 129 | 43 | 160 | 10 | 14 |
| Ibrutinib-GSH (**3c**) | 129 | 35 | 150 | 10 | 12 |
| **25** | 76 | 24 | 50 | 10 | 11 |
| **26** | 28 | 23 | 70 | 10 | 17 |
| **27** | 76 | 25 | 80 | 10 | 11 |
| **28** | 46 | 28 | 70 | 10 | 11 |
| **29** | 182 | 28 | 80 | 10 | 11 |
| **Table S1 continue…** |  |  |  |  |  |
| Molecule | NL (Da) | CE (V) | DP (V) | EP (V) | CXP |
| **30** | 45 | 19 | 50 | 10 | 12 |
| **31** | 35 | 34 | 70 | 10 | 8 |
| **32** | 77 | 35 | 70 | 10 | 8 |
| **33** | 35 | 31 | 70 | 10 | 10 |
| **34** | 44 | 28 | 70 | 10 | 9 |
| **35** | 92 | 34 | 60 | 10 | 9 |
| **25a** | 56 | 17 | 70 | 10 | 9 |
| **26a** | 56 | 17 | 70 | 10 | 9 |
| **27a** | 56 | 15 | 70 | 10 | 11 |
| **28a** | 56 | 11 | 50 | 10 | 11 |
| **29a** | 56 | 27 | 90 | 10 | 13 |
| **30a** | 56 | 23 | 70 | 10 | 10 |
| **31a** | 56 | 22 | 70 | 10 | 9 |
| **32a** | 56 | 24 | 80 | 10 | 10 |
| **33a** | 56 | 24 | 70 | 10 | 18 |
| **34a** | 56^b^ | N/A | 70 | 10 | 9 |
| **35a** | 56 | 14 | 80 | 10 | 11 |

^a^ 5th highest fragment used. ^b^ 4^th^ highest fragment used.

### Table S2 LC-MS and MS/MS analysis of failed Boc-L-cysteine adducts

| Molecule | LC-MS peak area | | Ratio  (Parent/Fragment) | Product ion scanning | | | | |
| --- | --- | --- | --- | --- | --- | --- | --- | --- |
|  | m/z | m/z ⎼ 100 Da |  | NL (Da) | CE (V) | DP (V) | EP (V) | CXP (V) |
| **S1** | 2E+6 | 1E+7 | 0.2 | 100 | 17 | 80 | 10 | 12 |
| **S2** | 3E+6 | 3E+7 | 0.1 | 100 | 17 | 70 | 10 | 11 |
| **S3** | 2E+7 | 3E+7 | 0.5 | 100 | 18 | 70 | 10 | 9 |
| **S4** | 2E+7 | 1E+7 | 1.9 | 100 | 17 | 70 | 10 | 13 |
| **S5** | 3E+7 | 4E+7 | 0.7 | 100 | 16 | 60 | 10 | 10 |
| **S6** | 1E+7 | 2E+7 | 0.8 | 100 | 18 | 70 | 10 | 12 |
| **S7** | 1E+7 | 4E+7 | 0.3 | 100 | 16 | 60 | 10 | 10 |
| **S8** | N/D | N/D | N/A | N/A | N/A | N/A | N/A | N/A |
| **S9** | 1E+7 | 7E+6 | 2.1 | 100 | 22 | 110 | 10 | 11 |
| **S10** | trace | N/D | N/A | 100 | 27 | 90 | 10 | 11 |
| **S11** | 3E+6 | 3E+7 | 0.1 | 100 | 16 | 80 | 10 | 11 |
| **S12** | N/D | 1E+7 | N/A | N/A | N/A | N/A | N/A | N/A |
| **S13** | 8E+6 | 2E+7 | 0.4 | 100 | 16 | 50 | 10 | 11 |
| **S14** | 6E+6 | 2E+7 | 0.3 | 100 | 17 | 60 | 10 | 12 |
| **S15** | N/D | 2E+7 | N/A | N/A | N/A | N/A | N/A | N/A |
| **S16** | 6E+6 | 4E+7 | 0.1 | 100 | 18 | 70 | 10 | 11 |
| **S17** | 2E+7 | 1E+7 | 2.0 | 100 | 17 | 50 | 10 | 12 |
| **S18** | 9E+6 | 4E+7 | 0.2 | 100 | 18 | 70 | 10 | 11 |
| **S19** | 3E+6 | 1E+7 | 0.2 | 100 | 17 | 100 | 10 | 9 |
| **S20** | 4E+7 | 2E+7 | 2.2 | 100 | 18 | 90 | 10 | 10 |
| **S21** | 1E+7 | 3E+7 | 0.4 | 100 | 17 | 70 | 10 | 11 |
| **S22** | 2E+7 | 2E+7 | 1.1 | 100 | 18 | 70 | 10 | 18 |
| **S23** | 1E+7 | 5E+7 | 0.3 | 100 | 17 | 70 | 10 | 11 |
| **S24** | 2E+6 | 2E+6 | 1.2 | 100 | 17 | 70 | 10 | 10 |
| **S25** | 1E+7 | 4E+7 | 0.3 | 100 | 15 | 60 | 10 | 11 |
| **S26** | 4E+6 | 3E+7 | 0.1 | 100 | 16 | 70 | 10 | 11 |
| **S27** | 3E+7 | 3E+7 | 0.9 | 100 | 18 | 70 | 10 | 11 |
| **S28** | 5E+7 | 1E+7 | 5.1 | 100 | 19 | 80 | 10 | 11 |
| **S29** | 1E+7 | 3E+7 | 0.5 | 100 | 18 | 70 | 10 | 11 |
| **S30** | 2E+6 | 6E+6 | 0.2 | 100 | 17 | 70 | 10 | 14 |
| **S31** | 5E+6 | 3E+7 | 0.2 | 100 | 16 | 70 | 10 | 11 |
| **S32** | 7E+6 | 4E+7 | 0.2 | 100 | 17 | 70 | 10 | 11 |
| **S33** | 1E+6 | 3E+7 | 0.0 | 100 | 16 | 50 | 10 | 11 |

**Table S2 continue…**

| Molecule | LC-MS peak area | | Ratio  (Parent/Fragment) | Product ion scanning | | | | |
| --- | --- | --- | --- | --- | --- | --- | --- | --- |
|  | m/z | m/z ⎼ 100 Da |  | NL (Da) | CE (V) | DP (V) | EP (V) | CXP (V) |
| **S34** | 8E+6 | 2E+7 | 0.4 | 100 | 18 | 70 | 10 | 11 |
| **S35** | 2E+7 | 5E+7 | 0.4 | 100 | 15 | 60 | 10 | 11 |
| **S36** | 2E+7 | 5E+7 | 0.4 | 100 | 17 | 60 | 10 | 11 |
| **S37** | 2E+7 | 5E+7 | 0.3 | 100 | 17 | 60 | 10 | 21 |
| **S38** | 3E+7 | 4E+7 | 0.7 | 100 | 17 | 70 | 10 | 9 |
| **S39** | 1E+7 | 4E+7 | 0.3 | 100 | 14 | 70 | 10 | 12 |
| **S40** | 4E+7 | 4E+7 | 0.9 | 100 | 17 | 70 | 10 | 11 |
| **S41** | 5E+5 | 2E+6 | 0.3 | 100 | 17 | 70 | 10 | 10 |
| **S42** | 1E+7 | 2E+7 | 0.4 | 100 | 17 | 90 | 10 | 10 |
| **S43** | 2E+7 | 4E+7 | 0.5 | 100 | 17 | 60 | 10 | 10 |
| **S44** | 1E+7 | 2E+7 | 0.7 | 100 | 15 | 60 | 10 | 11 |
| **S45** | N/D | 2E+7 | N/A | N/A | N/A | N/A | N/A | N/A |
| **S46** | 2E+7 | 5E+7 | 0.4 | 100 | 16 | 60 | 10 | 12 |
| **S47** | 1E+7 | 4E+7 | 0.3 | 100^a^ | N/A | 50 | 10 | 19 |
| **S48** | 1E+7 | 4E+7 | 0.2 | 100 | 18 | 70 | 10 | 11 |

^a^ 4^th^ highest fragment used.

### Table S3 AE-MS parameters

| Probe | NL (Da) | CE (V) | DP (V) | EP (V) | CXP |
| --- | --- | --- | --- | --- | --- |
| Boc-L-cysteine | 100 | 34 | 110 | 10 | 15 |
| L-cysteine | 89 | 45 | 133 | 10 | 13 |
| glutathione | 129 | 39 | 153 | 10 | 15 |
| thiophenol **36** | 56 | 19 | 72 | 10 | 11 |
|  |  |  |  |  |  |

## 2.4 Liquid chromatography-mass spectrometry (LC-MS)

The chromatographic system utilized was a Waters Acquity I class ultra-performance liquid chromatography (UPLC) system, comprising a binary pump, integrated degasser, column oven, autosampler, and sample organizer, all sourced from Waters Corporation (Milford, MA, USA). Separation was carried out on an Acquity UPLC BEH C18 1.7 μm, 2.1 x 50 mm column from Waters Corporation (Milford, MA, USA). Data acquisition utilized Masslynx v. 4.2, with analysis conducted using the Openlynx software suite. UV data within the 220-350 nm range was captured using an Acquity photodiode array detector. The flow was evenly split, with one half directed to an evaporative light scattering detector (ELSD) and the other to a single quadrupole (SQ) mass spectrometer. The total flow rate was maintained at 1.0 mL/min, with a sample injection volume of 2 or 5 μL. The UPLC column temperature was set at 63 °C, and the gradient program began at 90% A (0.1 % formic acid in MilliQ H_2_O), transitioning to 70% A over 0.2 minutes, then to 95% B (0.1 % formic acid in acetonitrile) over 1.4 minutes, holding for 0.35 minutes, before returning to 90% A over 0.05 minutes. Positive-ion mode electrospray ionization was employed for the mass spectrometer, with the following parameters: capillary voltage of 3.5 kV, cone voltage of 30 V, source temperature of 150 °C, desolvation temperature of 350 °C, desolvation gas flow of 750 L/hr, and cone gas flow of 25 L/hr. Mass spectrometry data acquisition involved a full scan range from m/z = 50−1200 Da within a 0.2 s time frame. Single ion recording mass spectrometry was utilized for sample quantification of each compound.

## 2.5 Probe quench condition optimization

In triplicate 650 nL (10 mM in DMSO) of covalent inhibitors PD168393, sotorasib, and ibrutinib were transferred by acoustic liquid handling. Solutions of probe (L-cysteine, Boc-L-cysteine, or glutathione) and bases (triethyl amine (TEA) and 1,8-Diazabicyclo(5.4.0)undec-7-ene (DBU)) were made in DMSO as a concentration series spanning 100 eq. to 6.25 eq. Then, 50 µL of probe and base mixture was add to covalent inhibitors. The plate was centrifuged for 5 minutes at 1000 rpm to mix and left to react overnight before direct LC-MS analysis. The peak area (by MS) of covalent inhibitors PD168393, sotorasib, and ibrutinib and its corresponding probe adduct product was collected. Conversion was calculated by dividing adduct peak area by the sum of residual covalent inhibitor and the adduct peak areas.

# 3. Synthesis and Plate Reactions

## 3.1 Synthesis of pyridinyl acrylamide 5

Acryloyl chloride **S50** (0.467 mL, 5.78 mmol, 1.0 eq.) was dissolved in anhydrous CH_2_Cl_2_ (15 mL) and cooled to 0 °C. A solution of 6-bromopyridin-2-amine **S49** (1.0 g, 5.78 mmol, 1.0 eq.) and 4-methylmorpholine (699 µL, 6.36 mmol, 1.1 eq.) in CH_2_Cl_2_ (10 mL) was then added slowly. The reaction was stirred for 1 hour at 0 °C, then allowed to warm to room temperature gradually. The progress of the reaction was monitored via TLC and LC-MS. Stop the reaction when the spot of starting material disappeared or 3 hours by quenched with saturated aqueous NaHCO_3_ (50 mL) and extracted with CH_2_Cl_2_ (3 × 50 mL). The combined organic layers were dried over anhydrous Na_2_SO_4_, concentrated under vacuum, and the residue was purified by flash column chromatography on silica gel to afford pyridinyl acrylamide **5** (1.3 g, 99% yield) as a white solid powder. ^1^H NMR (500 MHz, CDCl_3_) δ 8.26 (d, *J* = 7.5 Hz, 1H), 8.10 (s, 1H), 7.57 (t, *J* = 7.9 Hz, 1H), 7.23 (d, *J* = 8.4 Hz, 1H), 6.46 (dd, *J* = 0.9, 17.0 Hz, 1H), 6.23 (dd, *J* = 16.9, 10.3 Hz, 1H), 5.84 (dd, *J* = 1.0, 10.3 Hz, 1H). ^13^C NMR (126 MHz, CDCl_3_) δ 163.6, 151.4, 140.8, 139.4, 130.7, 129.3, 123.9, 112.7.

**Synthesis of piperazinyl acrylamide 6**

The protocol was adopted from Engel *et. al.*^33^ and was slightly modified. Triethylamine (1.50 mL, 10.7 mmol, 2.0 eq.) was added to the stirring solution of tert-butyl piperazine-1-carboxylate **S51** (1.0 g, 5.37 mmol, 1.0 eq. ) in CH_2_Cl_2_ (15 mL) and cooled to 0 °C using an ice bath. Acryloyl chloride **S50** (0.48 mL, 5.91 mmol, 1.1 eq.) in CH_2_Cl_2_ (5 mL) and added dropwise over 5 minutes. The reaction mixture was then stirred at room temperature for 3 hours. The progress of the reaction was monitored via TLC (10% MeOH in CH_2_Cl_2_, Rf_product_ = 1, Rf_starting material_ = 0) and LC-MS (retention time = 0.57 min). The reaction mixture was washed three times with aqueous saturated NaHCO_3_ (3 × 10 mL) and twice with brine (2 × 10 mL). The organic layer was then dried over Na_2_SO_4,_ and after filtration, the solvent was removed under reduced pressure. The product **S52** (1.26 g, 5.24 mmol, 98% yield) was obtained as a white solid and was used for the next reaction without further purification. ^1^H NMR (500 MHz, CDCl_3_) δ 6.56 (dd, *J* = 16.8, 10.5 Hz, 1H), 6.31 (dd, *J* = 16.8, 1.8 Hz, 1H), 5.73 (dd, *J* = 10.6, 1.8 Hz, 1H), 3.66 (s, 2H), 3.53 (s, 2H), 3.45 (dd, *J* = 6.6, 3.9 Hz, 4H), 1.48 (s, 9H). ^13^C NMR (126 MHz, CDCl_3_) δ 165.7, 154.7, 128.5, 127.4, 80.5, 45.7, 41.9, 28.5.

**S52** (500 mg, 2.08 mmol, 1.0 eq.) was dissolved in 20% trifluoroacetic acid in CH_2_Cl_2_ (25.0 mL), and the reaction mixture was stirred at room temperature for 3 hours. The progress of the reaction was monitored via TLC (10% MeOH, Rf_starting material_ = 1, Rf_product_ = 0). The solvent was evaporated under reduced pressure, and the residue was co-distilled with CH_2_Cl_2_ three times to afford piperazinyl acrylamide **6** (467 mg, 1.84 mmol, 88%) as a yellowish oil of high purity. ^1^H NMR (500 MHz, DMSO-*d_6_*) δ 8.81 (s, 2H), 6.80 (dd, *J* = 16.7, 10.4 Hz, 1H), 6.16 (dd, *J* = 16.7, 2.3 Hz, 1H), 5.79 – 5.73 (m, 1H), 3.74 (d, *J* = 26.0 Hz, 4H), 3.13 (s, 4H). ^13^C NMR (126 MHz, DMSO-*d_6_*) δ 164.5, 158.3 (q, *J* = 36.1 Hz, TFA), 128.3, 127.6, 115.8 (q, *J* = 293.3 Hz, TFA), 42.8 (d, *J* = 37.7 Hz), 42.0.

## 3.2 Pyridinyl acrylamide 5 Buchwald-Hartwig C-N coupling reaction

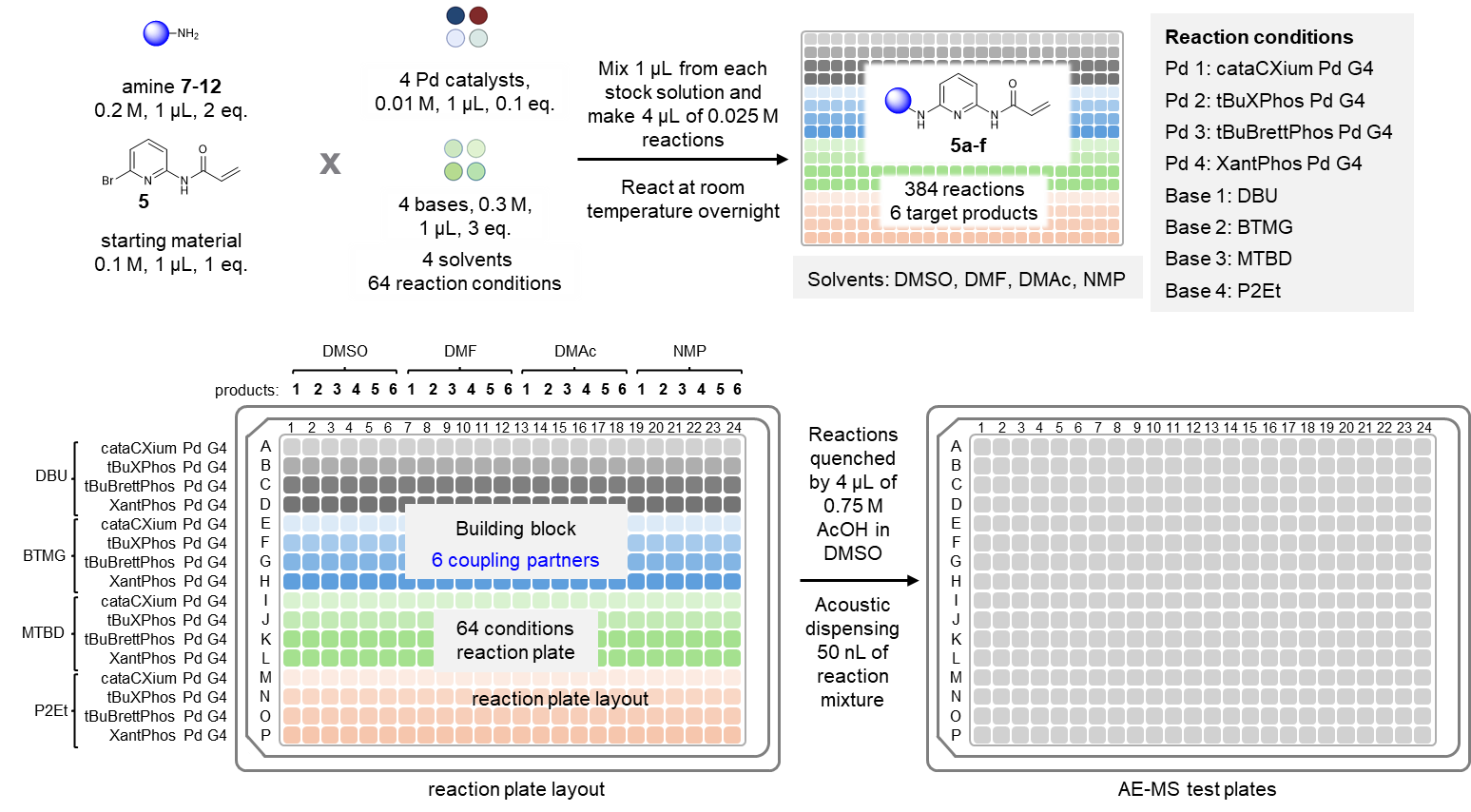


All stock solutions were prepared inside the glovebox using anhydrous solvents: pyridinyl acrylamide **5** (0.1 M in DMSO, DMF, DMAc, and NMP, 1 eq.). *Amines* **7-12** (0.2 M in DMSO, DMF, DMAc, and NMP, 2 eq.). *Palladium* catalysts cataCXium Pd G4, tBuXPhos Pd G4, tBuBrettPhos Pd G4, and XantPhos Pd G4 (0.01 M in DMSO, DMF, DMAc, and NMP, 0.1 eq.) *Bases* DBU, BTMG, MTBD, and P2Et (0.3 M in DMSO, DMF, DMAc, and NMP, 3 eq.). Using multi-channel pipetting 1 µL of each of the four components were arrayed in the following manner: **5** was added across every well of a 384-well COC plate (001-12782) separated by solvent class DMSO, columns 1-6; DMF, columns 7-12; DMAc, columns 13-18; DMP, columns 19-24. Next, *amines* **7-12** were added to their respective columns following the same pattern of solvents, i.e. **7** column 1 (DMSO), column 7 (DMF), column 13 (DMAc), and column 19 (NMP); **8** column 2 (DMSO), column 8 (DMF), … *Palladium* catalysts were distributed by column to each solvent grouping arrayed every 4^th^ row (i.e. cataCXium Pd G4 rows A, E, I, and M). *Bases* were distributed by column to each solvent grouping arrayed as identical blocks of 4 (i.e. DBU rows A-D). Upon completion of the transfers the plates were centrifuged for ~1 minute at 1000 rpm and then immediately sealed with polyolefin sealing tape (Thermo 232701). The mixtures were left to stand at room temperature for 20 h. Upon completion plates were removed from the glovebox and quenched through addition of acetic acid (AcOH) in DMSO (4 µL, 0.75 M) using a Multidrop combi nL.

Analysis plates were generated as follows by acoustic liquid handling:

AE-MS – the 384-well COC reaction plate (001-12782) was directly copied to a single 384-well PP plate (001-14615) (50 nL per source well). 50 µL of freshly made DMSO/H_2_O (*v:v* = 70:30) solution containing 1.25 mM Boc-L-cysteine and 2.5 mM DBU was add to each well by multidrop Combi nL. The plate was centrifuged for 5 minutes at 1000 rpm and then left to react at room temperature for overnight before AE-MS analysis.

LC-MS –a plate copy was generated from the 384-well reaction plate (001-12782) by creating direct 1-1 copies through transfer of 200 nL to a costar 384-well plate (3657). A 50 nL of internal standard warfarin at 10 mM in DMSO was transferred to each well. The plate was diluted using a multidrop combi nL with DMSO (50 µL) to provide a final maximum concentration of 50 µM.

## 3.3 Pyridinyl acrylamide 5 Suzuki–Miyaura C-C coupling reaction

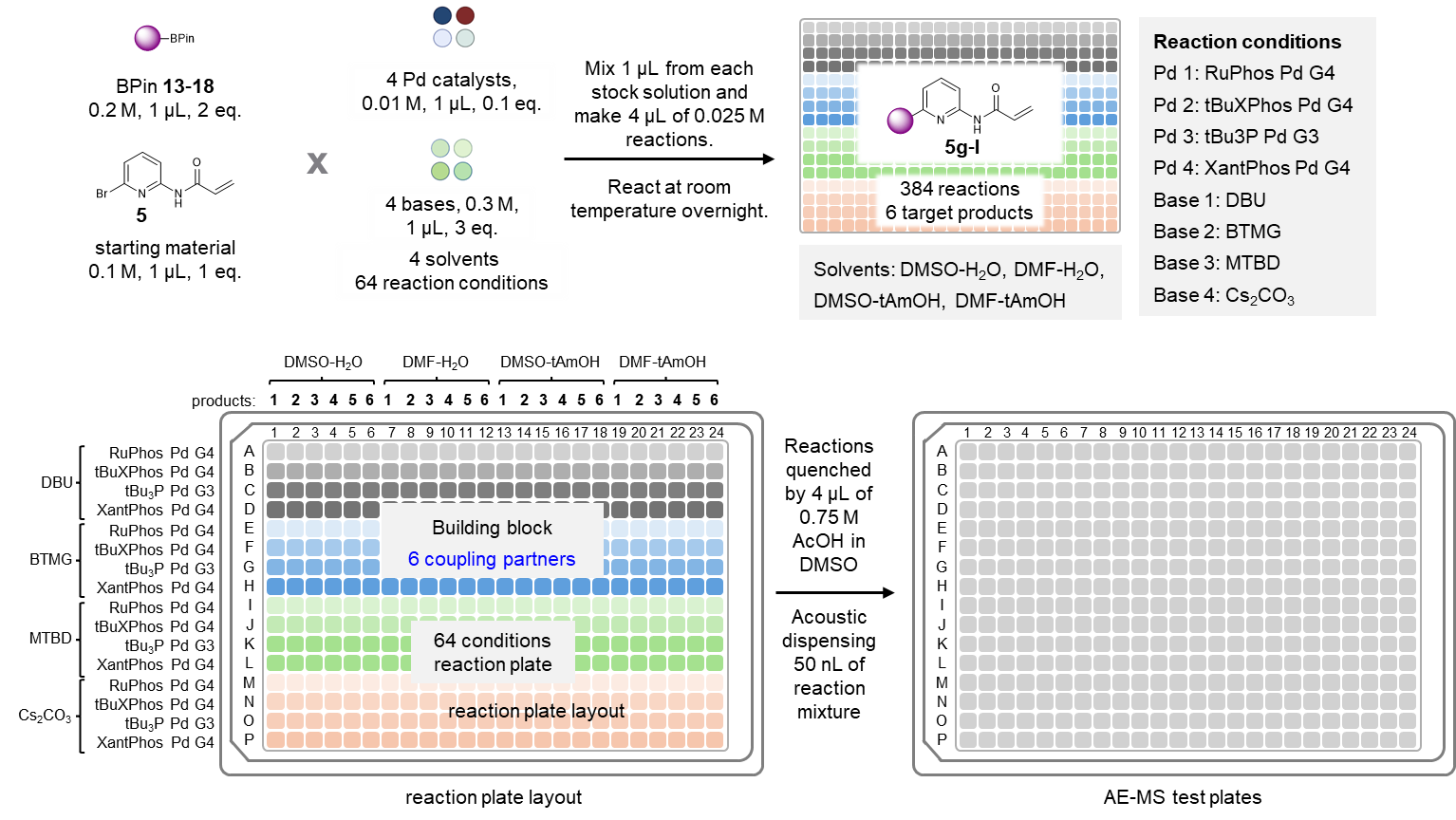


All stock solutions were prepared inside the glovebox using anhydrous solvents: pyridinyl acrylamide **5** (0.1 M in DMSO and DMF, 1 eq.). *Boronic acid pinacol ester (Bpin)* **13-18** (0.2 M in DMSO and DMF, 2 eq.). *Palladium* catalysts RuPhos Pd G4, tBuXPhos Pd G4, tBu3P Pd G3, and XantPhos Pd G4 (0.01 M in DMSO and DMF, 0.1 eq.) *Bases* DBU, BTMG, MTBD, and Cs_2_CO_3_ (0.3 M in H_2_O and tAmOH, 3 eq.). Using multi-channel pipetting 1 µL of each of the four components were arrayed in the following manner: **5** was added across every well of a 384-well COC plate (001-12782) separated by solvent class DMSO, columns 1-6; DMF, columns 7-12; DMSO, columns 13-18; DMF, columns 19-24. Next, *boronic acid pinacol ester* (Bpin) **13-18** were added to their respective columns following the same pattern of solvents, i.e. **13** column 1 (DMSO), column 7 (DMF), column 13 (DMSO), and column 19 (DMSO); **14** column 2 (DMSO), column 8 (DMF), … Palladium catalysts were distributed by column to each solvent grouping arrayed every 4^th^ row (i.e. RuPhos Pd G4 rows A, E, I, and M). Bases in H_2_O were distributed to column 1 to 12 arrayed as identical blocks of 4 (i.e. DBU rows A-D). Bases in tAmOH were distributed to column 13 to 24 arrayed as identical blocks of 4 (i.e. DBU rows A-D). Upon completion of the transfers the plates were centrifuged for ~1 minute at 1000 rpm and then immediately sealed with polyolefin sealing tape (Thermo 232701). The mixtures were left to stand at room temperature for 20 h. Upon completion plates were removed from the glovebox and quenched through addition of AcOH in DMSO (4 µL, 0.75 M) using a multidrop combi nL.

Analysis plates were generated as follows by acoustic liquid handling:

AE-MS – the 384-well COC reaction plate (001-12782) was directly copied to a single 384-well PP plate (001-14615) (50 nL per source well). 50 µL of freshly made DMSO/H_2_O (*v:v* = 70:30) solution containing 1.25 mM Boc-L-cysteine and 2.5 mM DBU was add to each well by multidrop Combi nL. The plate was centrifuged for 5 minutes at 1000 rpm and then left to react at room temperature for overnight before AE-MS analysis.

LC-MS –a plate copy was generated from the 384-well reaction plate (001-12782) by creating direct 1-1 copies through transfer of 200 nL to a costar 384-well plate (3657). A 50 nL of internal standard warfarin at 10 mM in DMSO was transferred to each well. The plate was diluted using a multidrop combi nL with DMSO (50 µL) to provide a final maximum concentration of 50 µM.

## 3.4 PD168393 Buchwald-Hartwig C-N coupling reaction

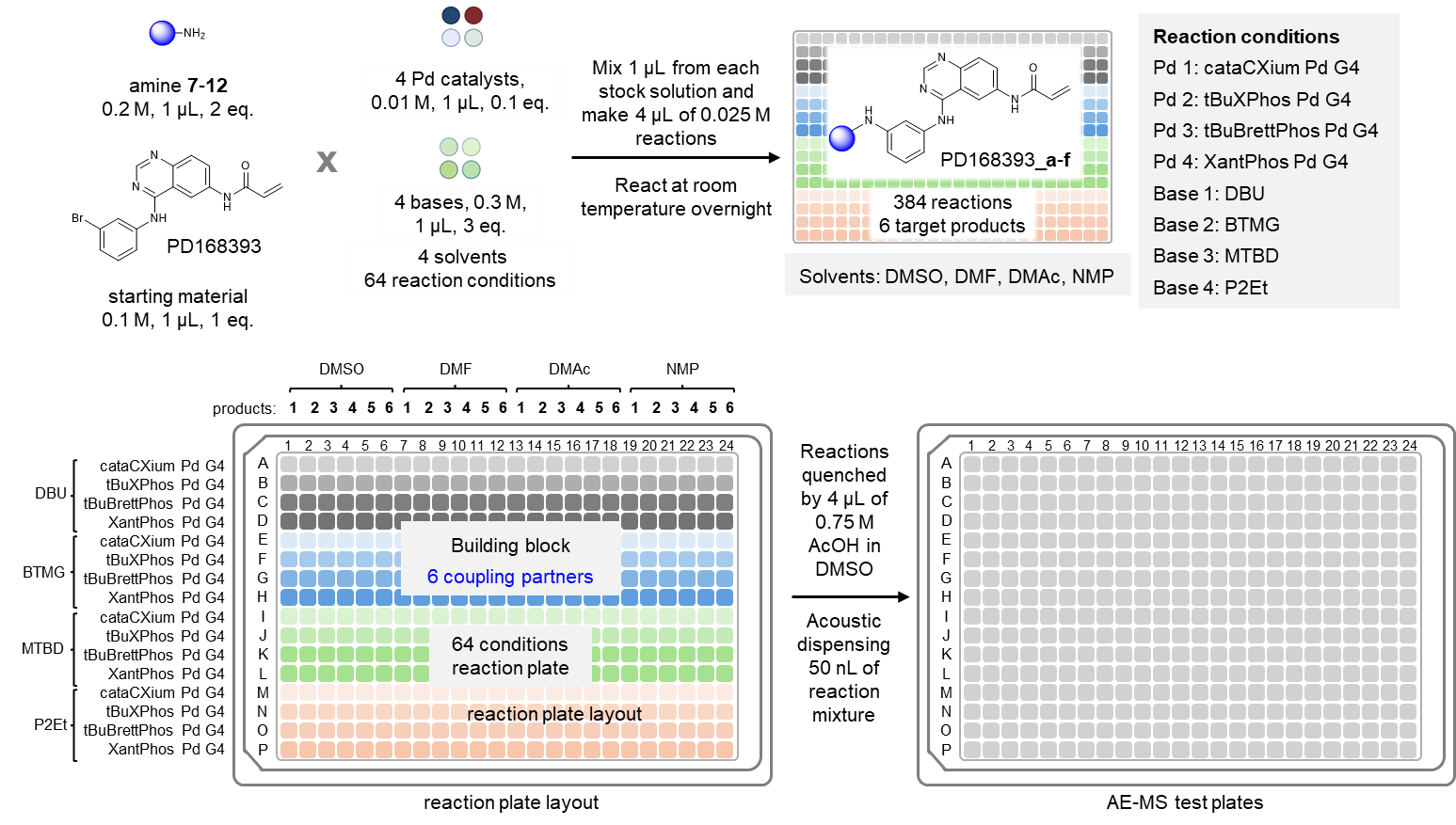


All stock solutions were prepared inside the glovebox using anhydrous solvents: PD168393 (0.1 M in DMSO, DMF, DMAc, and NMP, 1 eq.). *Amines* **7-12** (0.2 M in DMSO, DMF, DMAc, and NMP, 2 eq.). *Palladium* catalysts cataCXium Pd G4, tBuXPhos Pd G4, tBuBrettPhos Pd G4, and XantPhos Pd G4 (0.01 M in DMSO, DMF, DMAc, and NMP, 0.1 eq.) *Bases* DBU, BTMG, MTBD, and P2Et (0.3 M in DMSO, DMF, DMAc, and NMP, 3 eq.). Using multi-channel pipetting 1 µL of each of the four components were arrayed in the following manner: PD168393 was added across every well of a 384-well COC plate (001-12782) separated by solvent class DMSO, columns 1-6; DMF, columns 7-12; DMAc, columns 13-18; DMP, columns 19-24. Next, *amines* **7-12** were added to their respective columns following the same pattern of solvents, i.e. **7** column 1 (DMSO), column 7 (DMF), column 13 (DMAc), and column 19 (NMP); **8** column 2 (DMSO), column 8 (DMF), … *Palladium* catalysts were distributed by column to each solvent grouping arrayed every 4^th^ row (i.e. cataCXium Pd G4 rows A, E, I, and M). *Bases* were distributed by column to each solvent grouping arrayed as identical blocks of 4 (i.e. DBU rows A-D). Upon completion of the transfers the plates were centrifuged for ~1 minute at 1000 rpm and then immediately sealed with polyolefin sealing tape (Thermo 232701). The mixtures were left to stand at room temperature for 20 h. Upon completion plates were removed from the glovebox and quenched through addition of acetic acid (AcOH) in DMSO (4 µL, 0.75 M) using a multidrop combi nL.

Analysis plates were generated as follows by acoustic liquid handling:

AE-MS – the 384-well COC reaction plate (001-12782) was directly copied to a single 384-well PP plate (001-14615) (50 nL per source well). 50 µL of freshly made DMSO/H_2_O (*v:v* = 70:30) solution containing 1.25 mM Boc-L-cysteine and 2.5 mM DBU was add to each well by multidrop Combi nL. The plate was centrifuged for 5 minutes at 1000 rpm and then left to react at room temperature for overnight before AE-MS analysis.

LC-MS –a plate copy was generated from the 384-well reaction plate (001-12782) by creating direct 1-1 copies through transfer of 200 nL to a costar 384-well plate (3657). A 50 nL of internal standard warfarin at 10 mM in DMSO was transferred to each well. The plate was diluted using a multidrop combi nL with DMSO (50 µL) to provide a final maximum concentration of 50 µM.

## 3.5 PD168393 Suzuki–Miyaura C-C coupling reaction

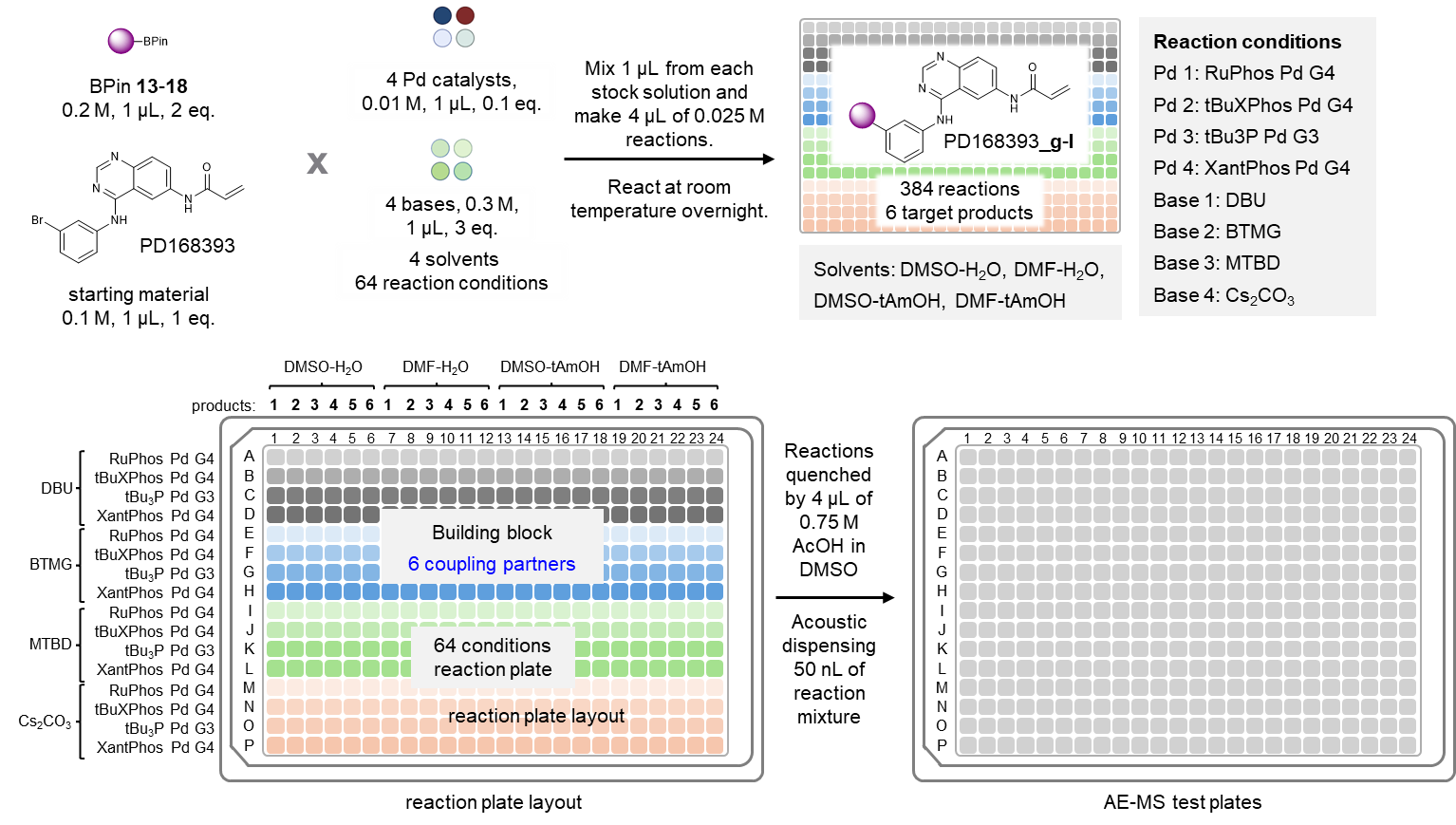


All stock solutions were prepared inside the glovebox using anhydrous solvents: PD168393 (0.1 M in DMSO and DMF, 1 eq.). *Boronic acid pinacol ester (Bpin)* **13-18** (0.2 M in DMSO and DMF, 2 eq.). *Palladium* catalysts RuPhos Pd G4, tBuXPhos Pd G4, tBu3P Pd G3, and XantPhos Pd G4 (0.01 M in DMSO and DMF, 0.1 eq.) *Bases* DBU, BTMG, MTBD, and Cs_2_CO_3_ (0.3 M in H_2_O and tAmOH, 3 eq.). Using multi-channel pipetting 1 µL of each of the four components were arrayed in the following manner: PD168393 was added across every well of a 384-well COC plate (001-12782) separated by solvent class DMSO, columns 1-6; DMF, columns 7-12; DMSO, columns 13-18; DMF, columns 19-24. Next, *boronic acid pinacol ester* (Bpin) **13-18** were added to their respective columns following the same pattern of solvents, i.e. **13** column 1 (DMSO), column 7 (DMF), column 13 (DMSO), and column 19 (DMSO); **14** column 2 (DMSO), column 8 (DMF), … Palladium catalysts were distributed by column to each solvent grouping arrayed every 4^th^ row (i.e. RuPhos Pd G4 rows A, E, I, and M). Bases in H_2_O were distributed to column 1 to 12 arrayed as identical blocks of 4 (i.e. DBU rows A-D). Bases in tAmOH were distributed to column 13 to 24 arrayed as identical blocks of 4 (i.e. DBU rows A-D). Upon completion of the transfers the plates were centrifuged for ~1 minute at 1000 rpm and then immediately sealed with polyolefin sealing tape (Thermo 232701). The mixtures were left to stand at room temperature for 20 h. Upon completion plates were removed from the glovebox and quenched through addition of AcOH in DMSO (4 µL, 0.75 M) using a multidrop combi nL.

Analysis plates were generated as follows by acoustic liquid handling:

Due to precipitation, 4 µL of DMSO was add to the 384-well COC reaction plate (001-12782) and centrifuged 5 minutes at 1000 rpm. Then 8 µL solution was transferred to another 384-well COC plate (001-12782) before the preparation of the analytical plate.

AE-MS – the 384-well COC new reaction plate (001-12782) was directly copied to a single 384-well PP plate (001-14615) (75 nL per source well). 50 µL of freshly made DMSO/H_2_O (*v:v* = 70:30) solution containing 1.25 mM Boc-L-cysteine and 2.5 mM DBU was add to each well by multidrop Combi nL. The plate was centrifuged for 5 minutes at 1000 rpm and then left to react at room temperature for overnight before AE-MS analysis.

LC-MS –a plate copy was generated from the 384-well reaction plate (001-12782) by creating direct 1-1 copies through transfer of 200 nL to a costar 384-well plate (3657). A 50 nL of internal standard warfarin at 10 mM in DMSO was transferred to each well. The plate was diluted using a multidrop combi nL with DMSO (50 µL) to provide a final maximum concentration of 50 µM.

## 3.6 Piperazinyl acrylamide 6 *N*-alkylation reaction


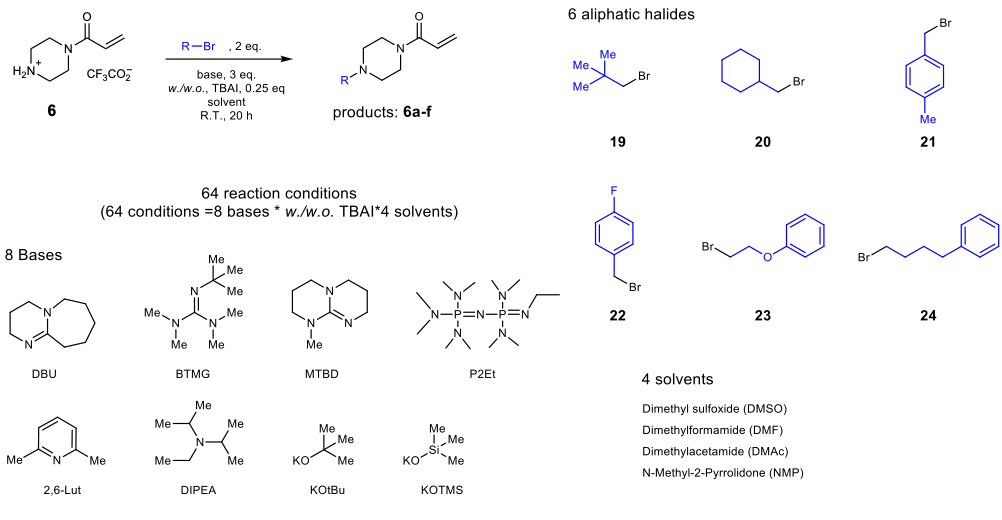


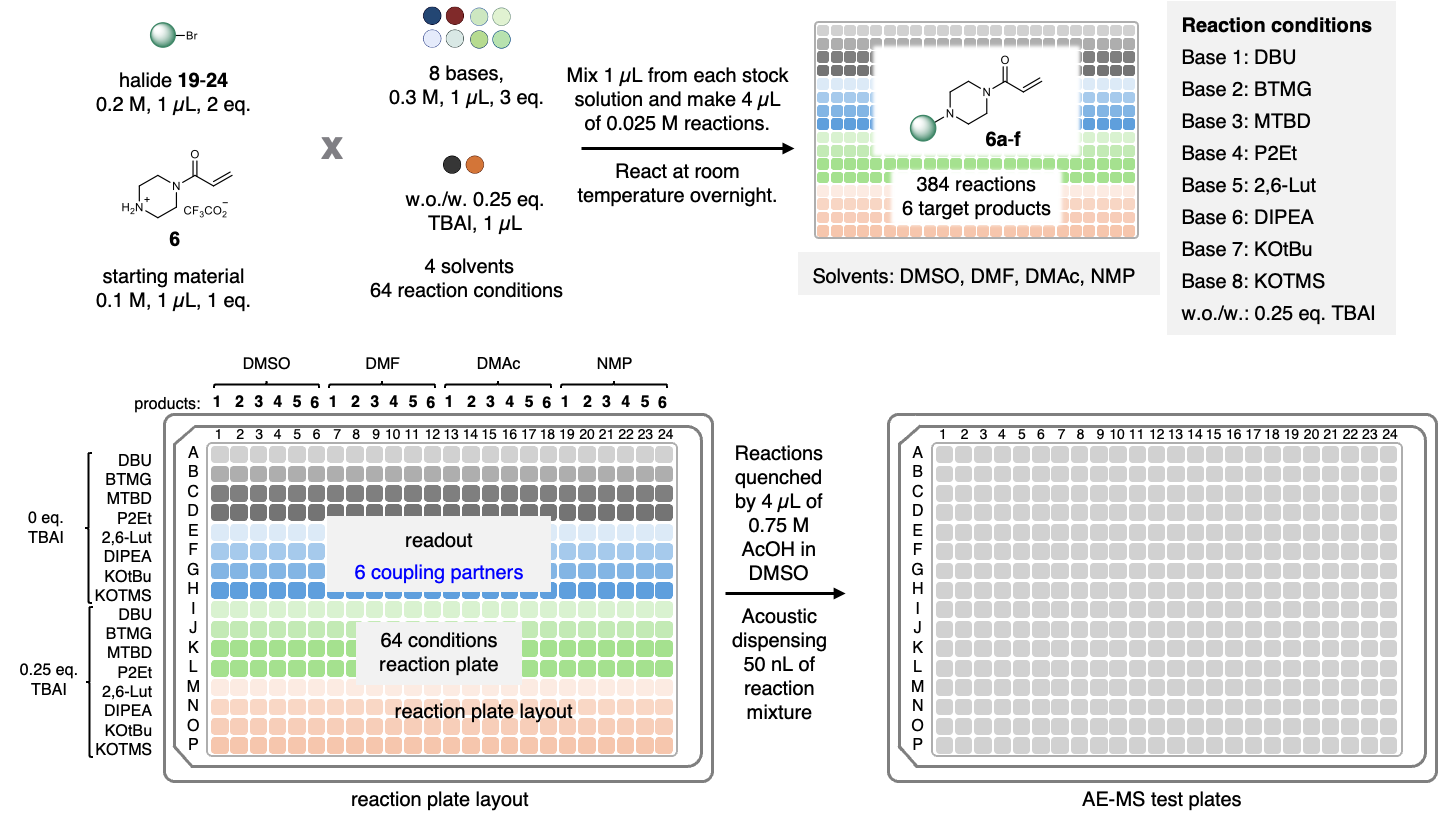


All stock solutions were prepared inside the glovebox using anhydrous solvents: piperazinyl acrylamide **6** piperazinyl acrylamide **6** (0.1 M in DMSO, DMF, DMAc, and NMP, 1 eq.). *Alkyl halides* **19-24** (0.2 M in DMSO, DMF, DMAc, and NMP, 2 eq.). *Bases* DBU, BTMG, MTBD, P2Et, 2,6-Lut, DIPEA, KOtBu, and KOTMS (0.3 M in DMSO, DMF, DMAc, and NMP, 3 eq.). Using multi-channel pipetting 1 µL of each of the four components were arrayed in the following manner: **6** was added across every well of a 384-well COC plate (001-12782) separated by solvent class DMSO, columns 1-6; DMF, columns 7-12; DMAc, columns 13-18; DMP, columns 19-24. Next, *alkyl halides* **19-24** were added to their respective columns following the same pattern of solvents, i.e. **19** column 1 (DMSO), column 7 (DMF), column 13 (DMAc), and column 19 (NMP); **20** column 2 (DMSO), column 8 (DMF), … *Bases* were distributed by column to each solvent grouping arrayed every 8^th^ row (i.e. DBU rows A and I). TBAI was distributed by column to each solvent grouping arrayed as 0 eq. of TBAI in row A-H, 0.25 eq. of TBAI in row I-P. Upon completion of the transfers the plates were centrifuged for ~1 minute at 1000 rpm and then immediately sealed with polyolefin sealing tape (Thermo 232701). The mixtures were left to stand at room temperature for 20 h. Upon completion plates were removed from the glovebox and quenched through addition of AcOH in DMSO (4 µL, 0.75 M) using a multidrop combi nL.

Analysis plates were generated as follows by acoustic liquid handling:

AE-MS – the 384-well COC reaction plate (001-12782) was directly copied to a single 384-well PP plate (001-14615) (50 nL per source well). 50 µL of freshly made DMSO/H_2_O (*v:v* = 70:30) solution containing 1.25 mM Boc-L-cysteine and 2.5 mM DBU was add to each well by multidrop Combi nL. The plate was centrifuged for 5 minutes at 1000 rpm and then left to react at room temperature for overnight before AE-MS analysis.

LC-MS –a plate copy was generated from the 384-well reaction plate (001-12782) by creating direct 1-1 copies through transfer of 200 nL to a costar 384-well plate (3657). A 50 nL of internal standard warfarin at 10 mM in DMSO was transferred to each well. The plate was diluted using a multidrop combi nL with DMSO (50 µL) to provide a final maximum concentration of 50 µM.

# 4. CID Spectra


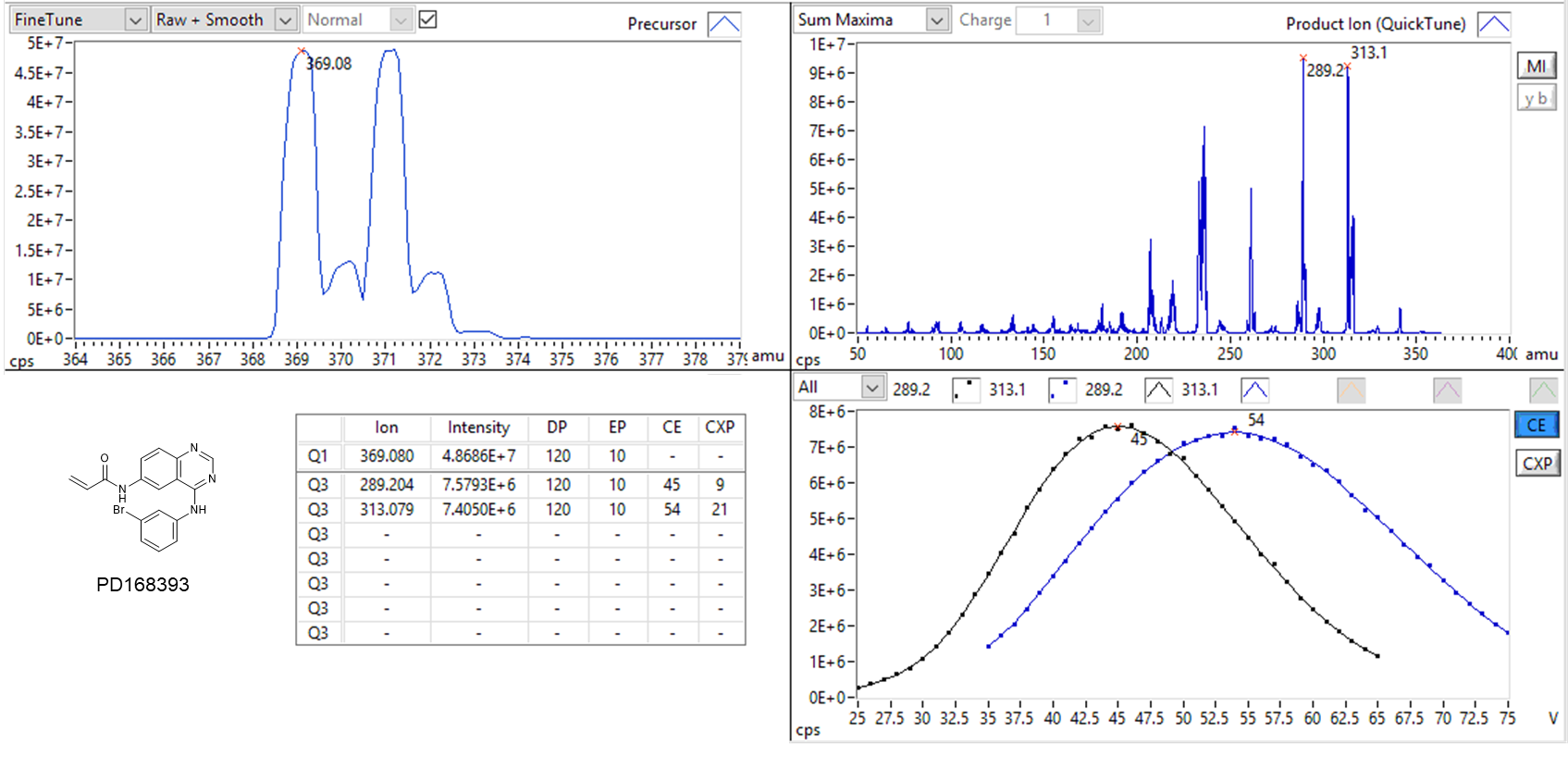


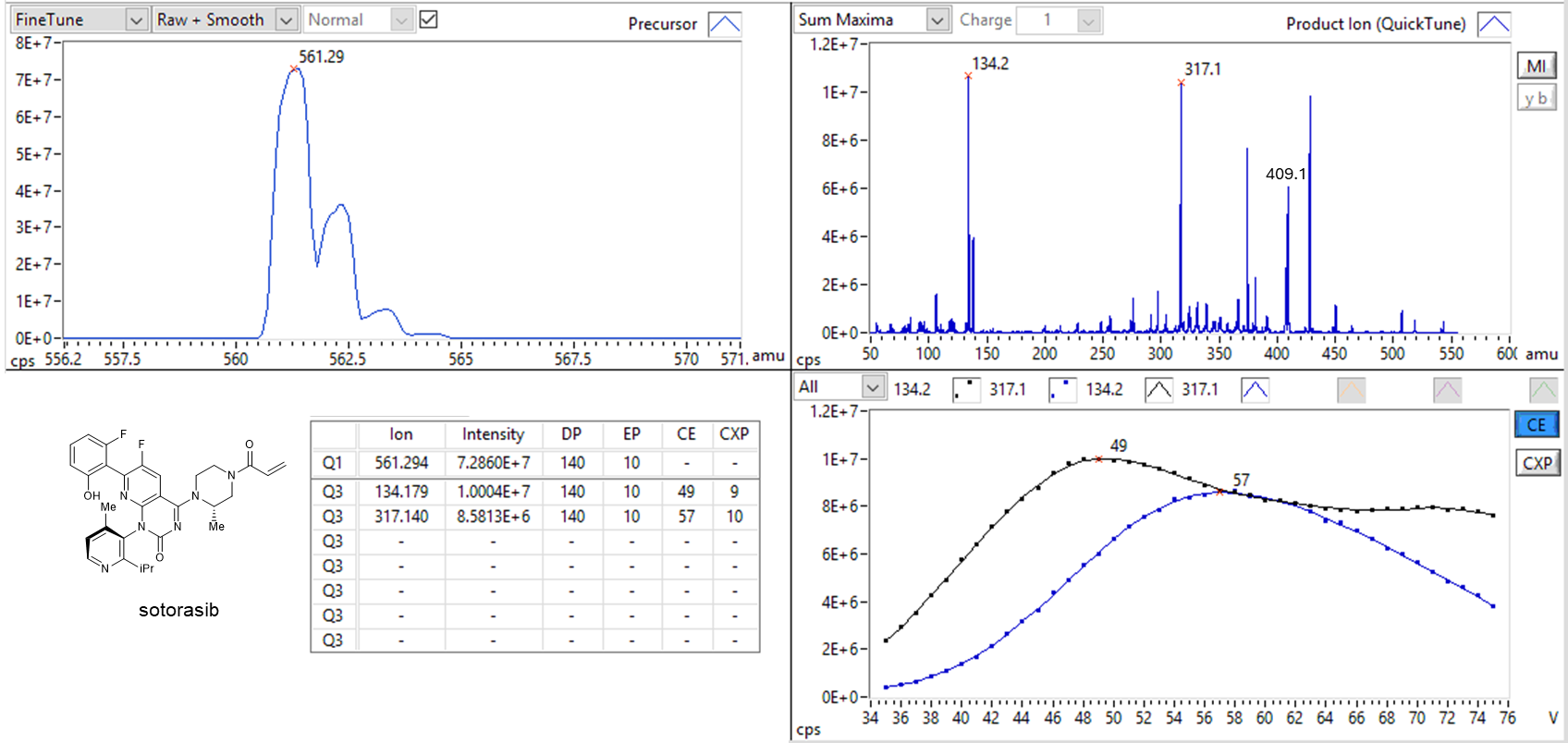


**
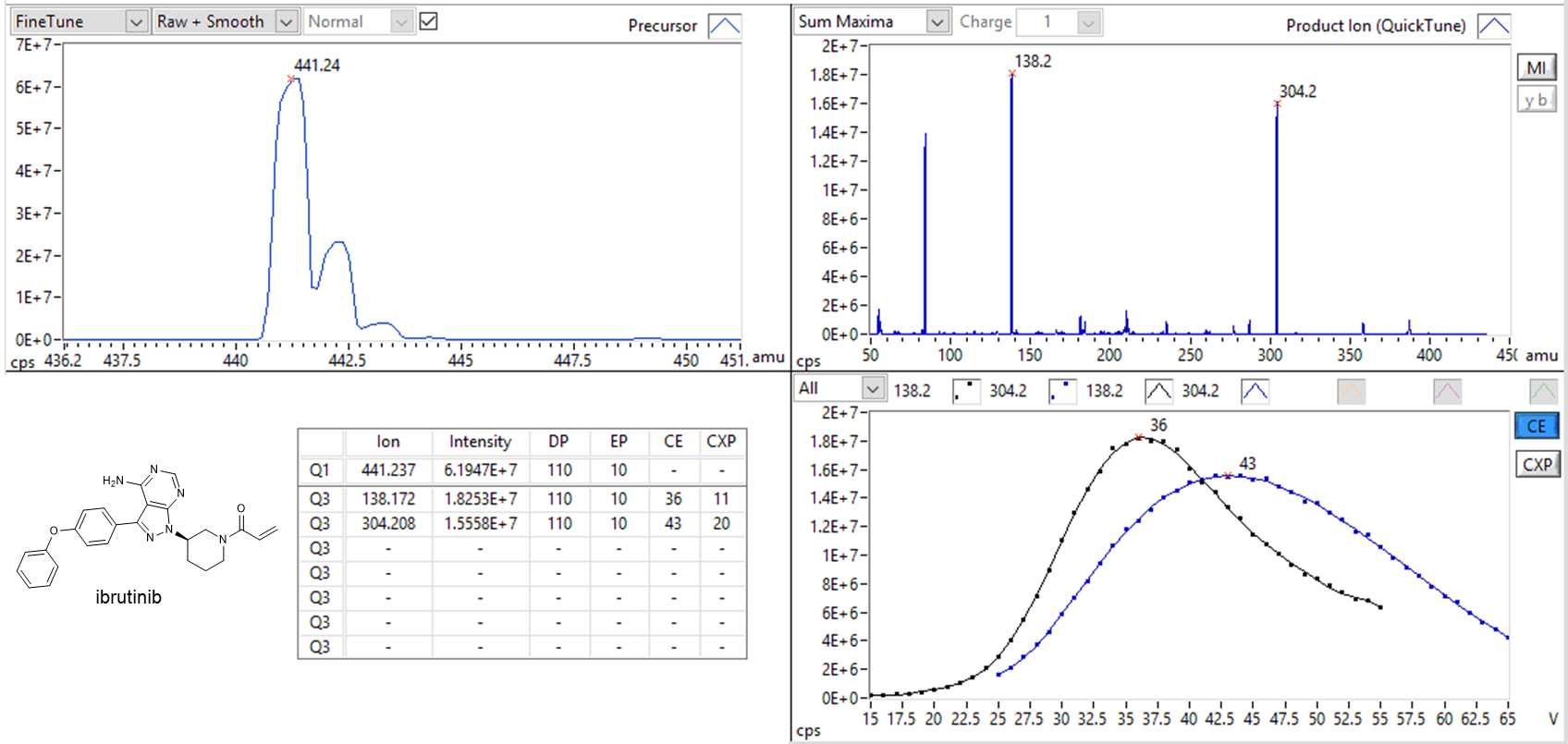
**

**
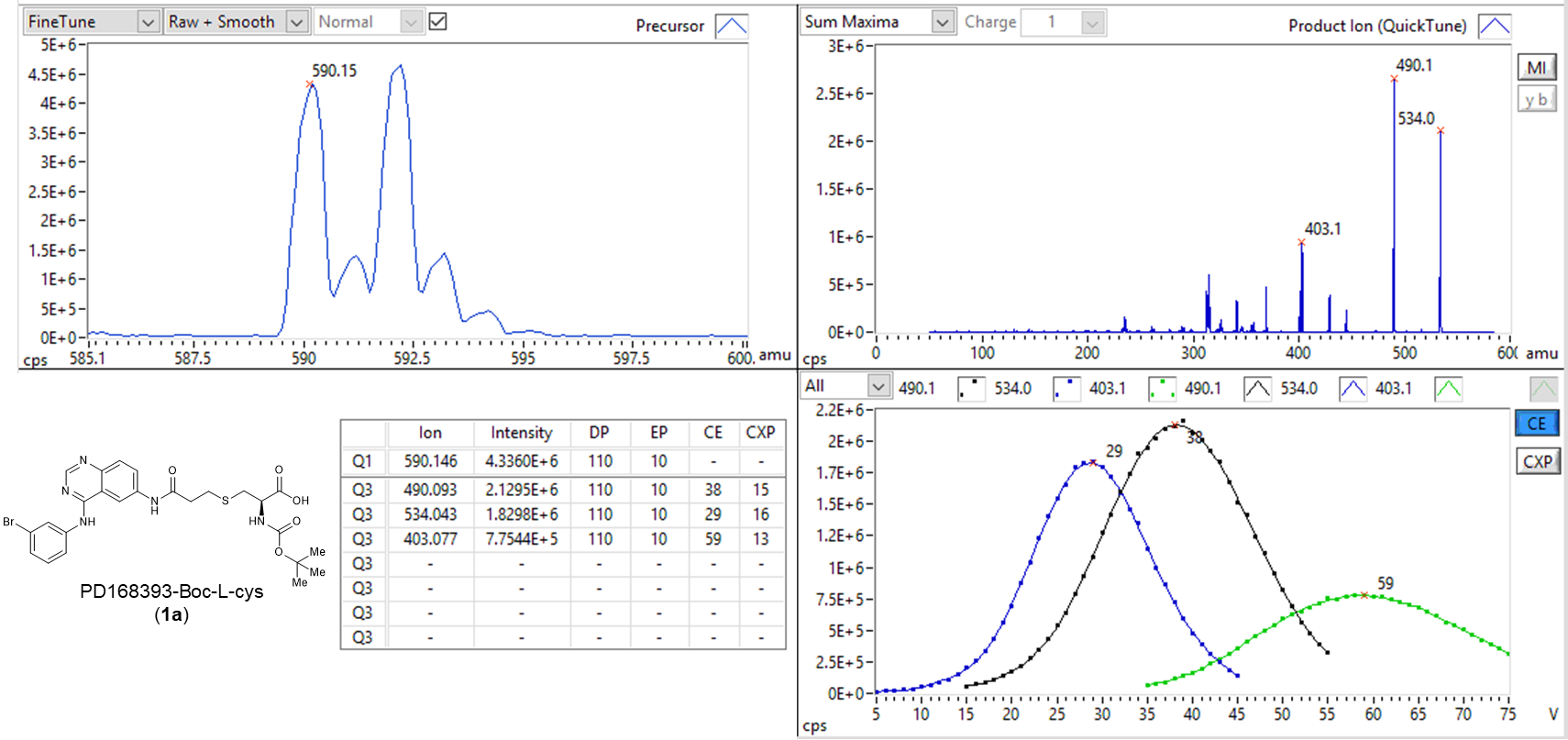
**

**
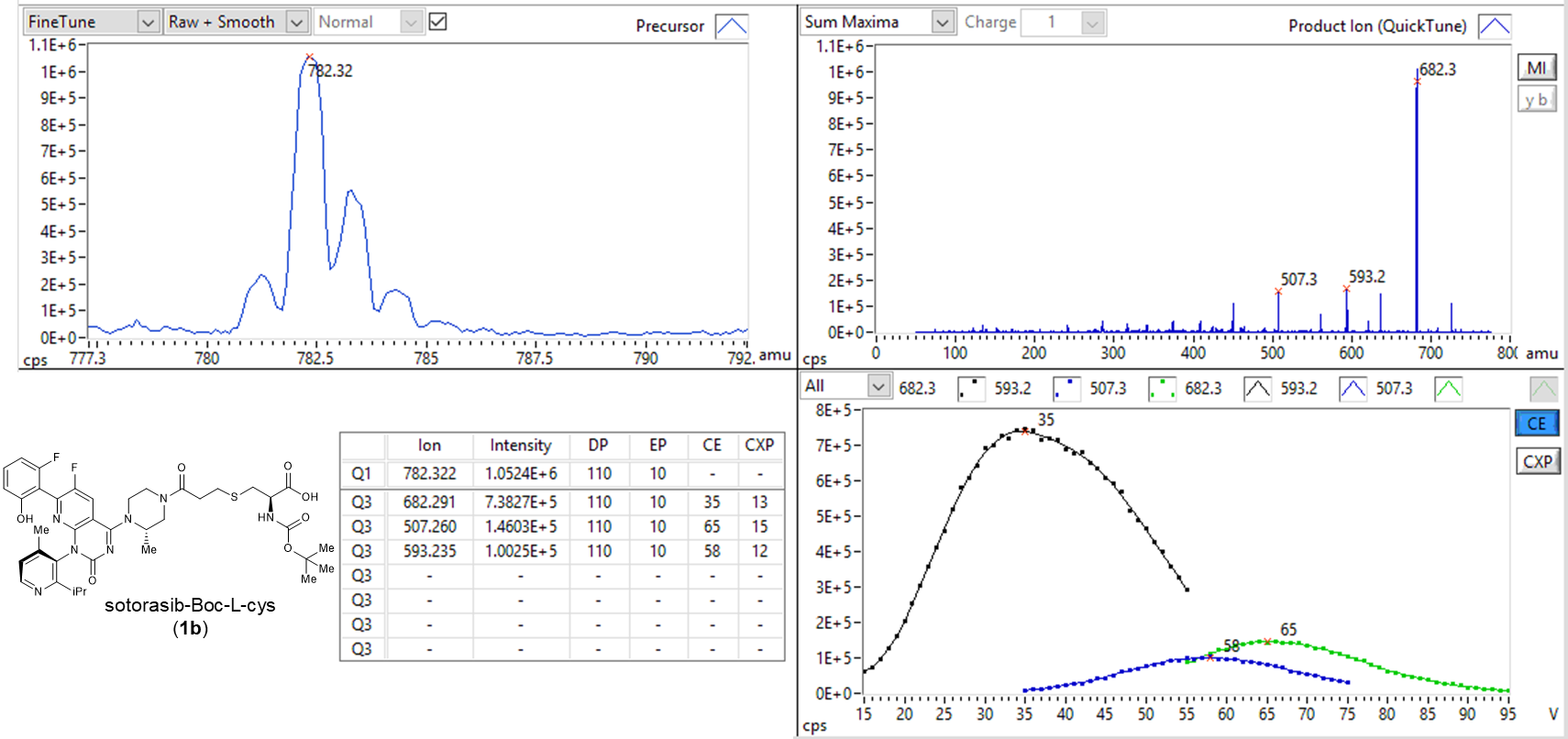
**

**
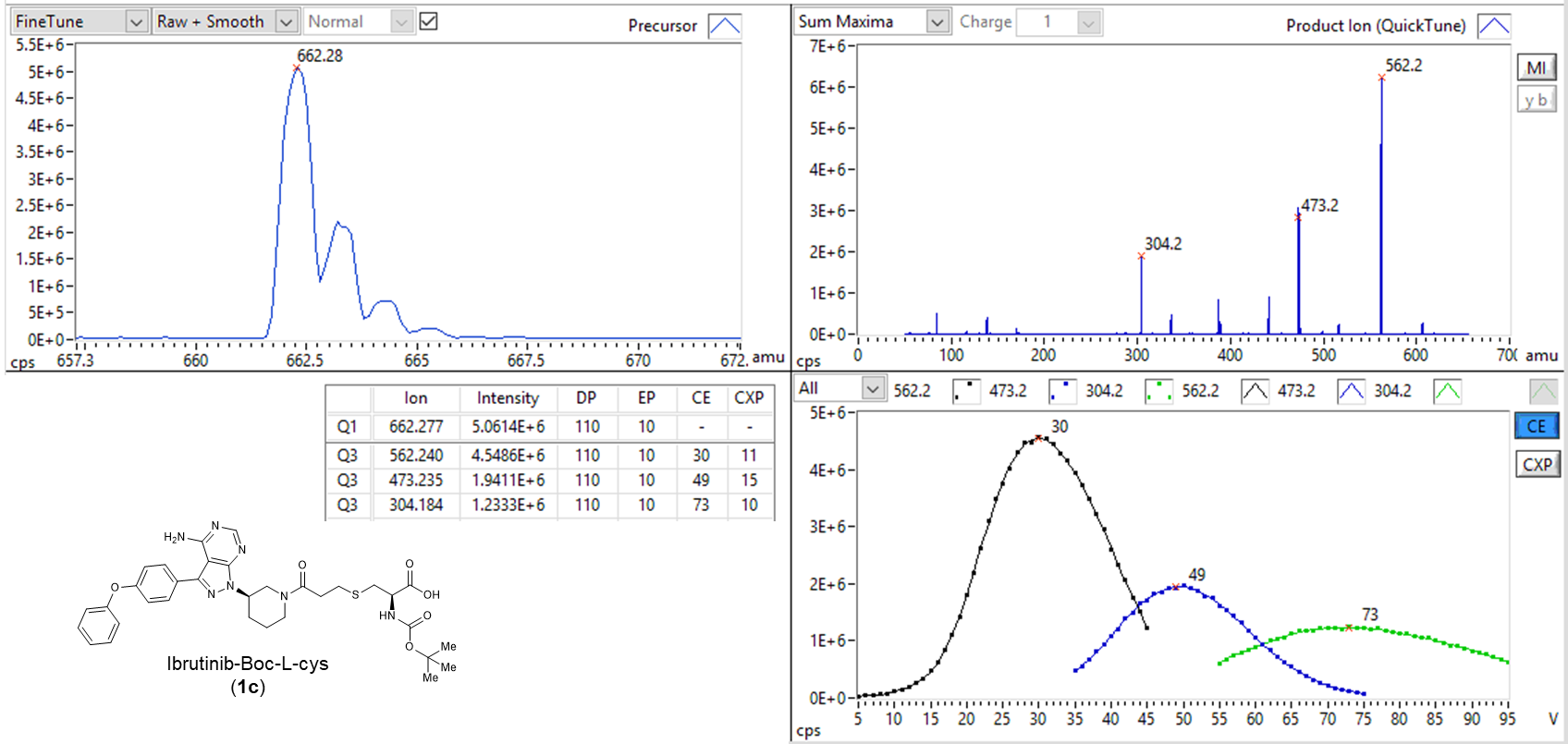
**

**
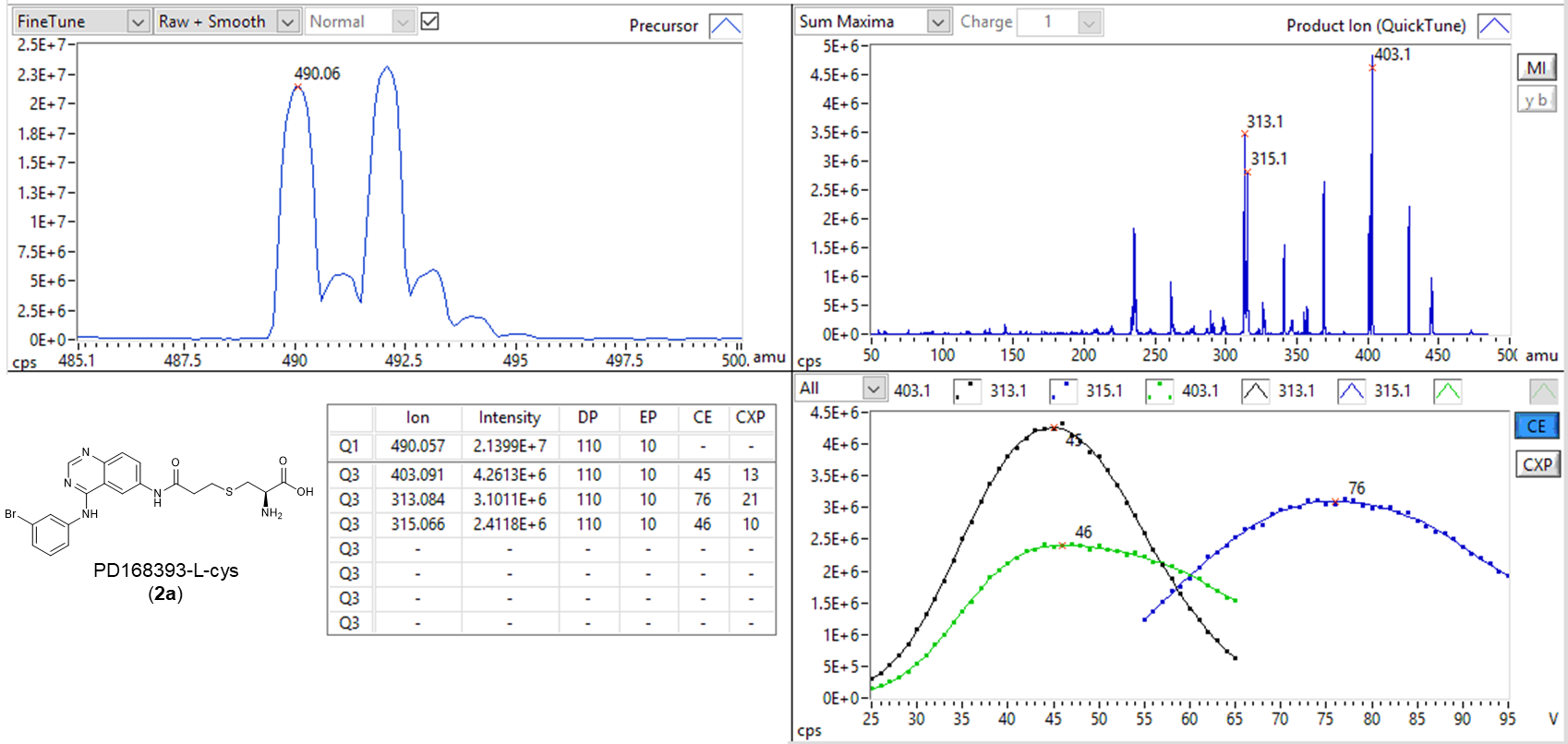
**

**
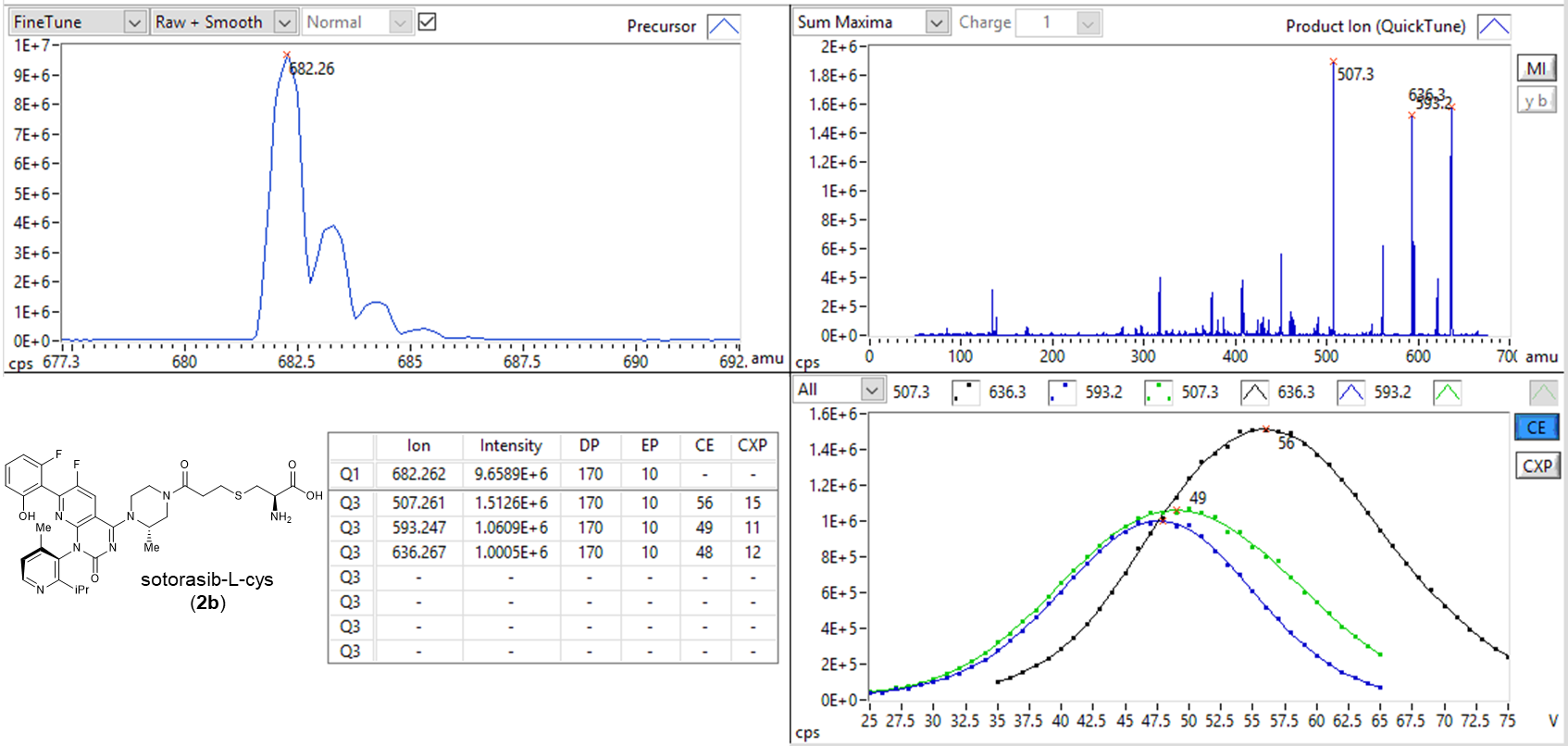
**

**
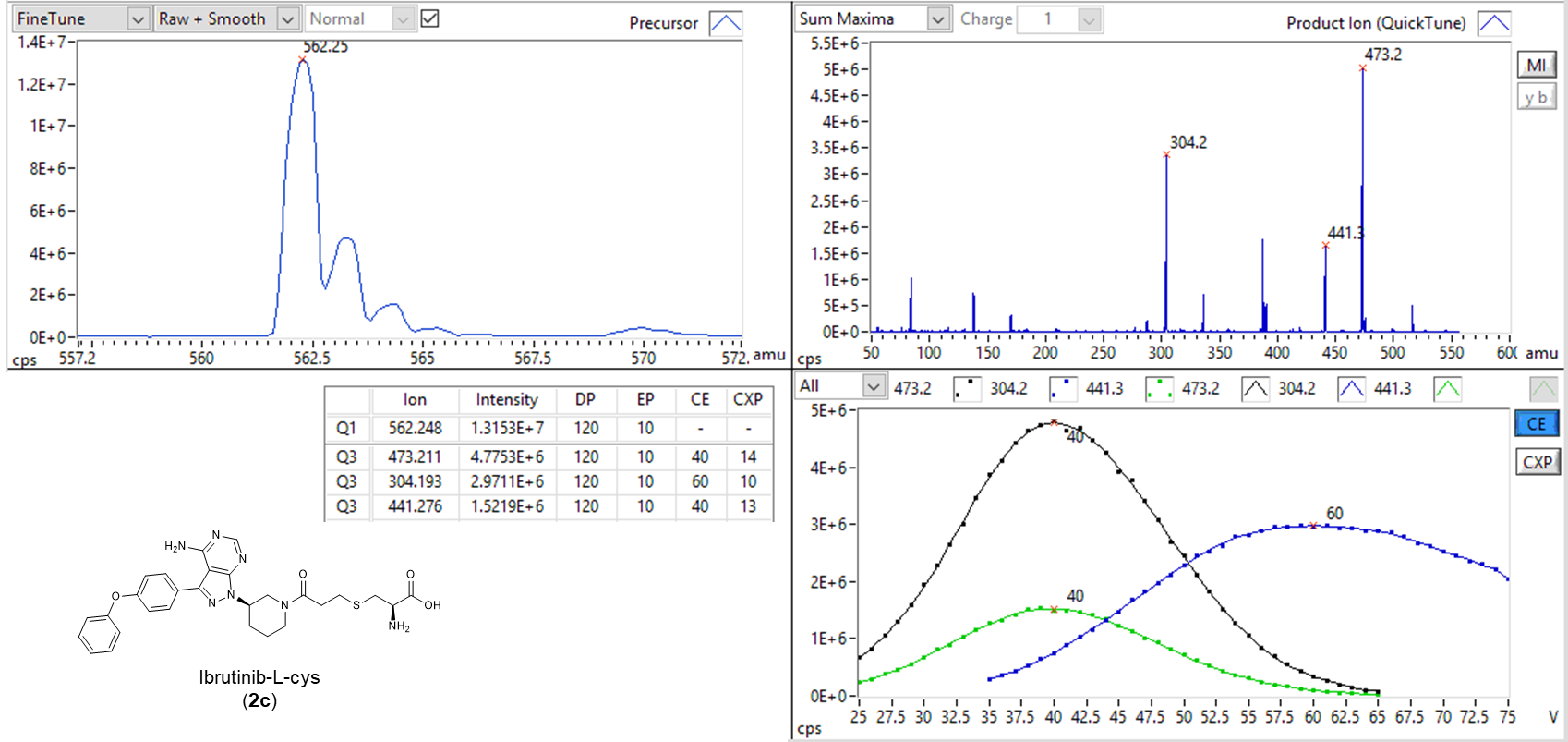
**

**
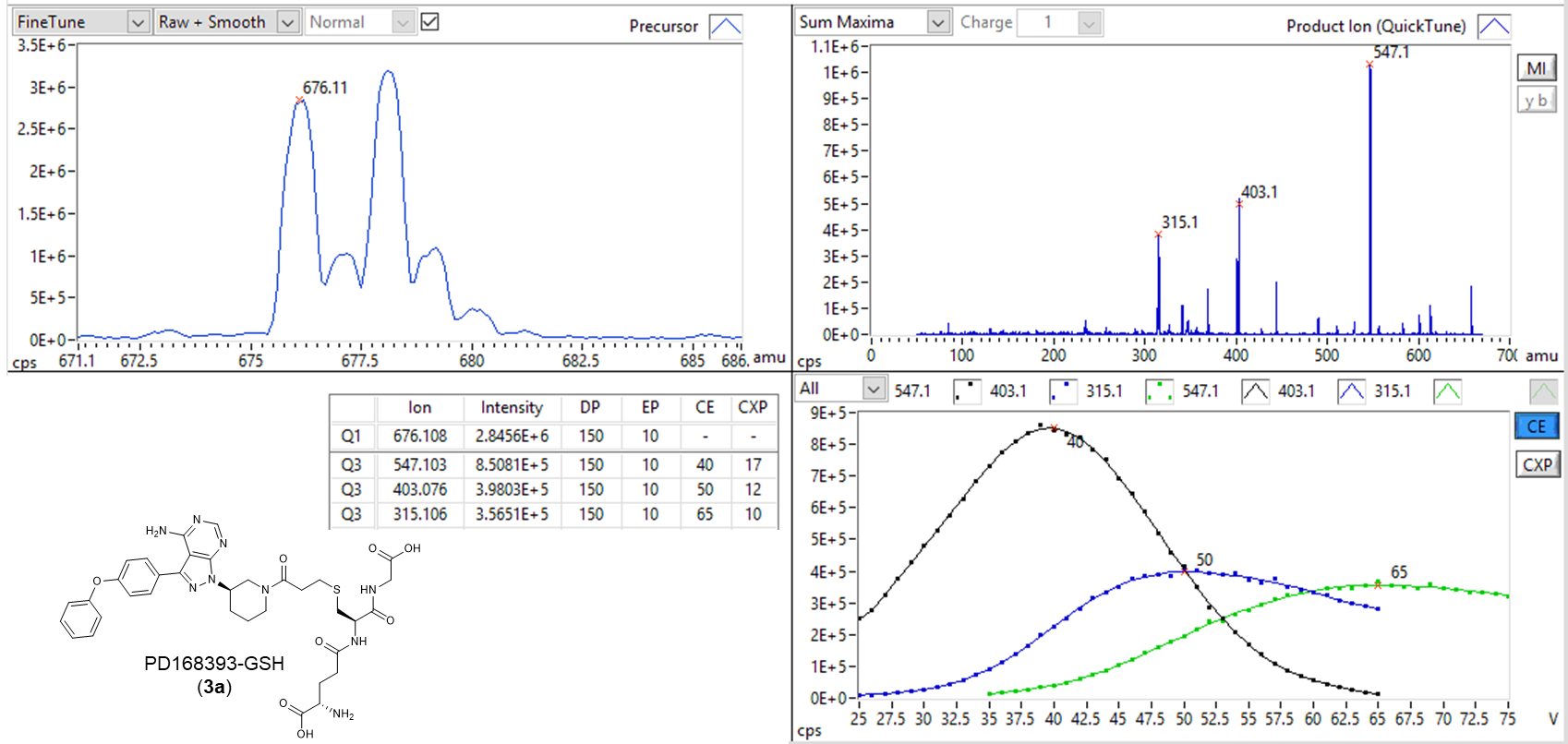
**

**
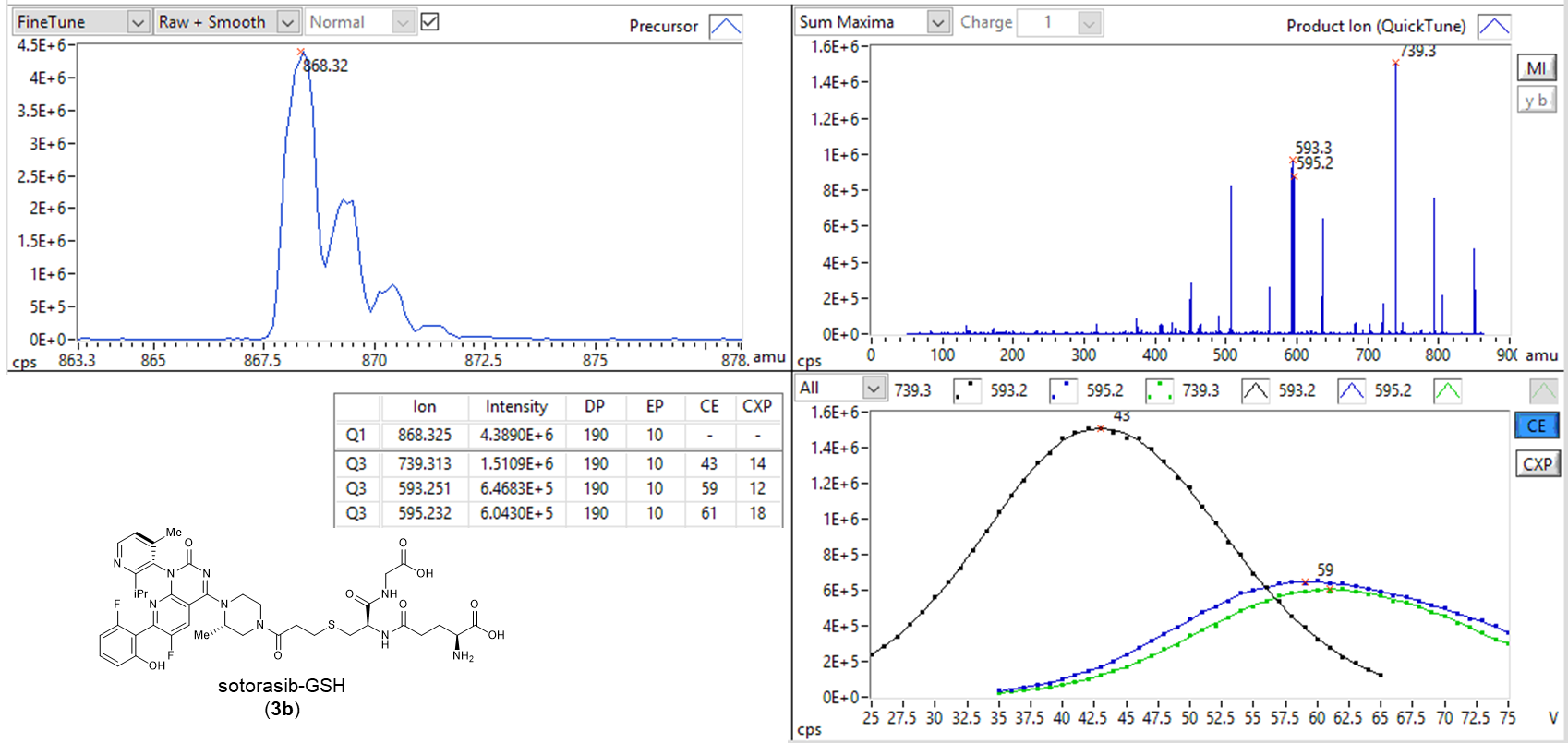
**

**
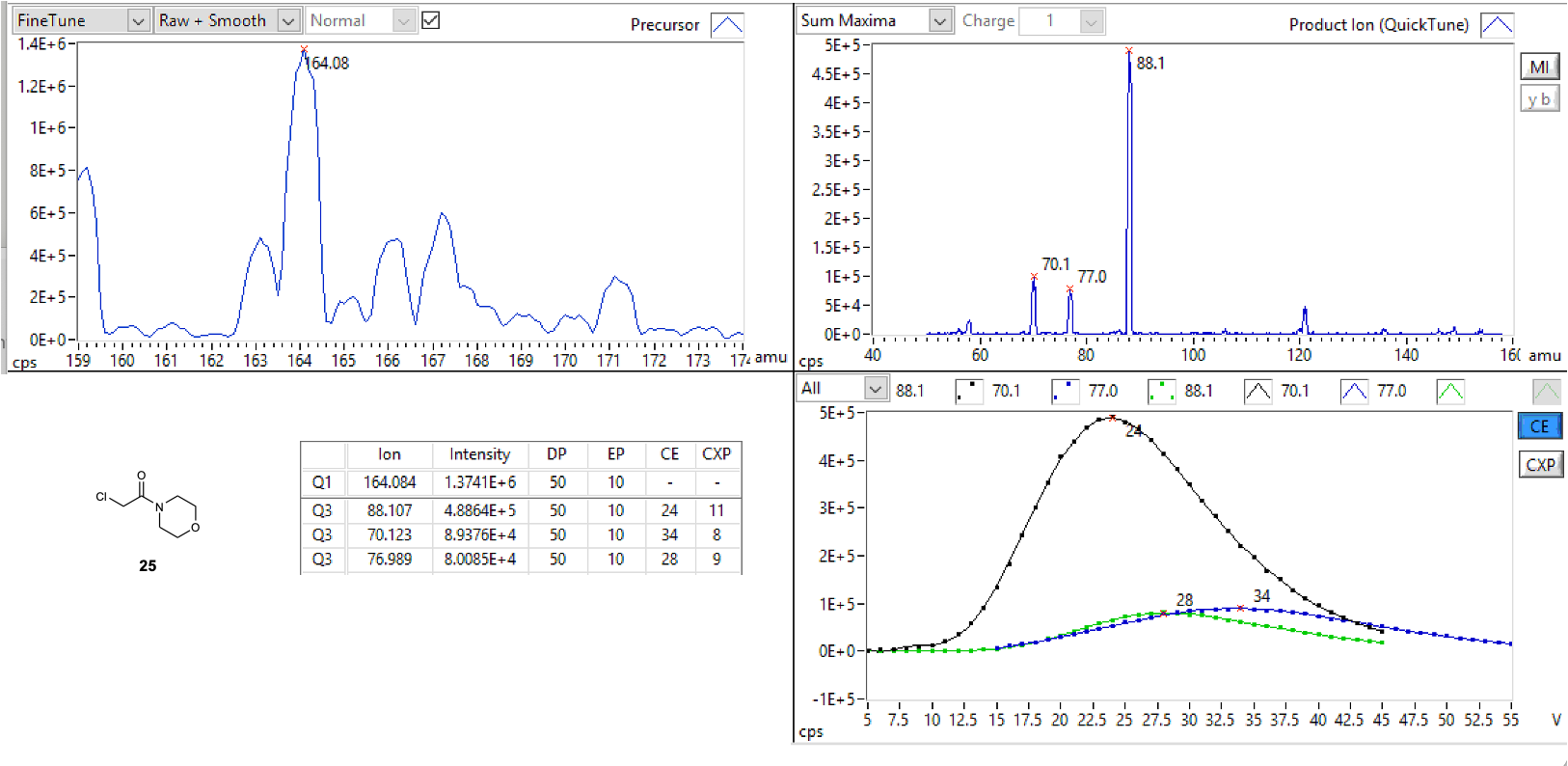
**

**
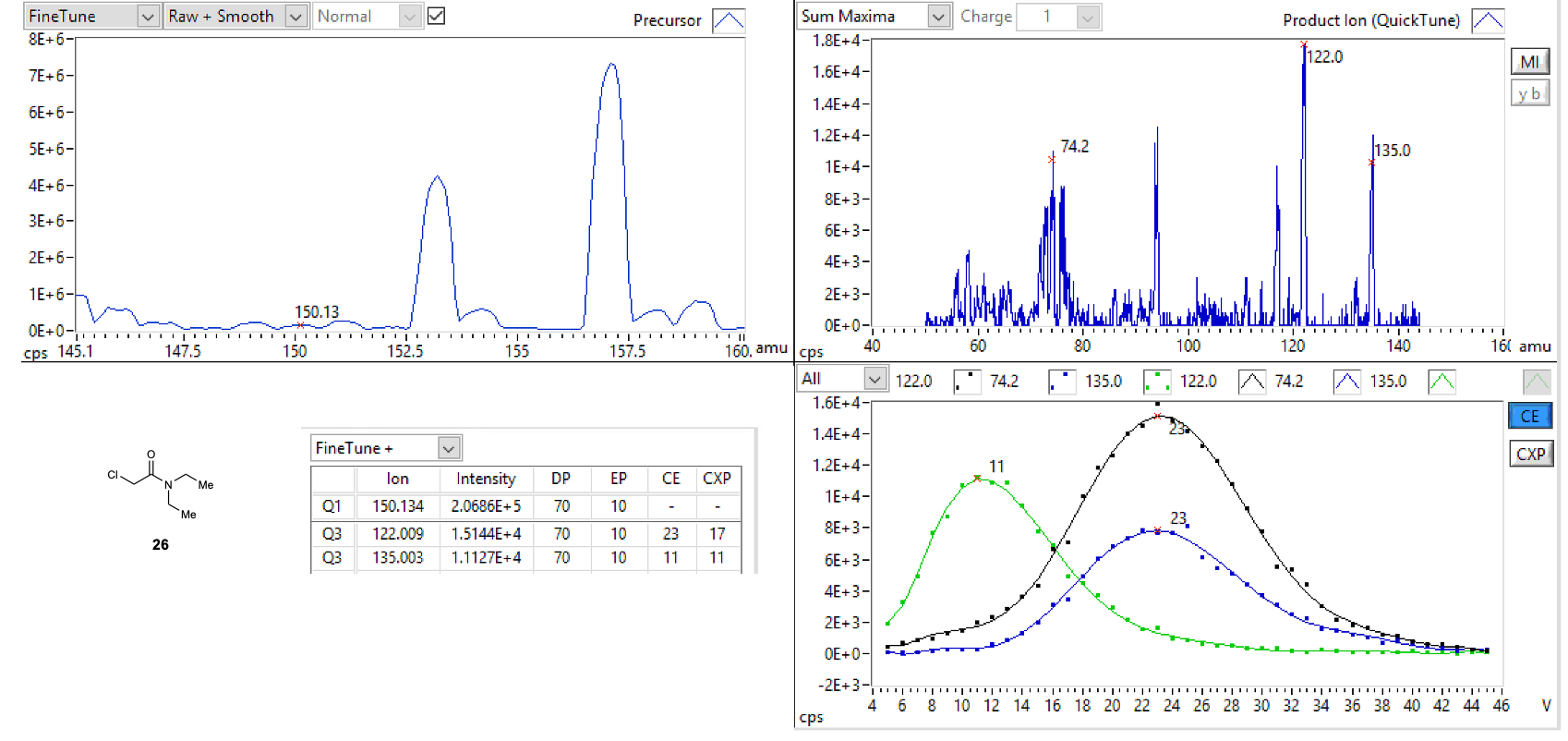
**

**
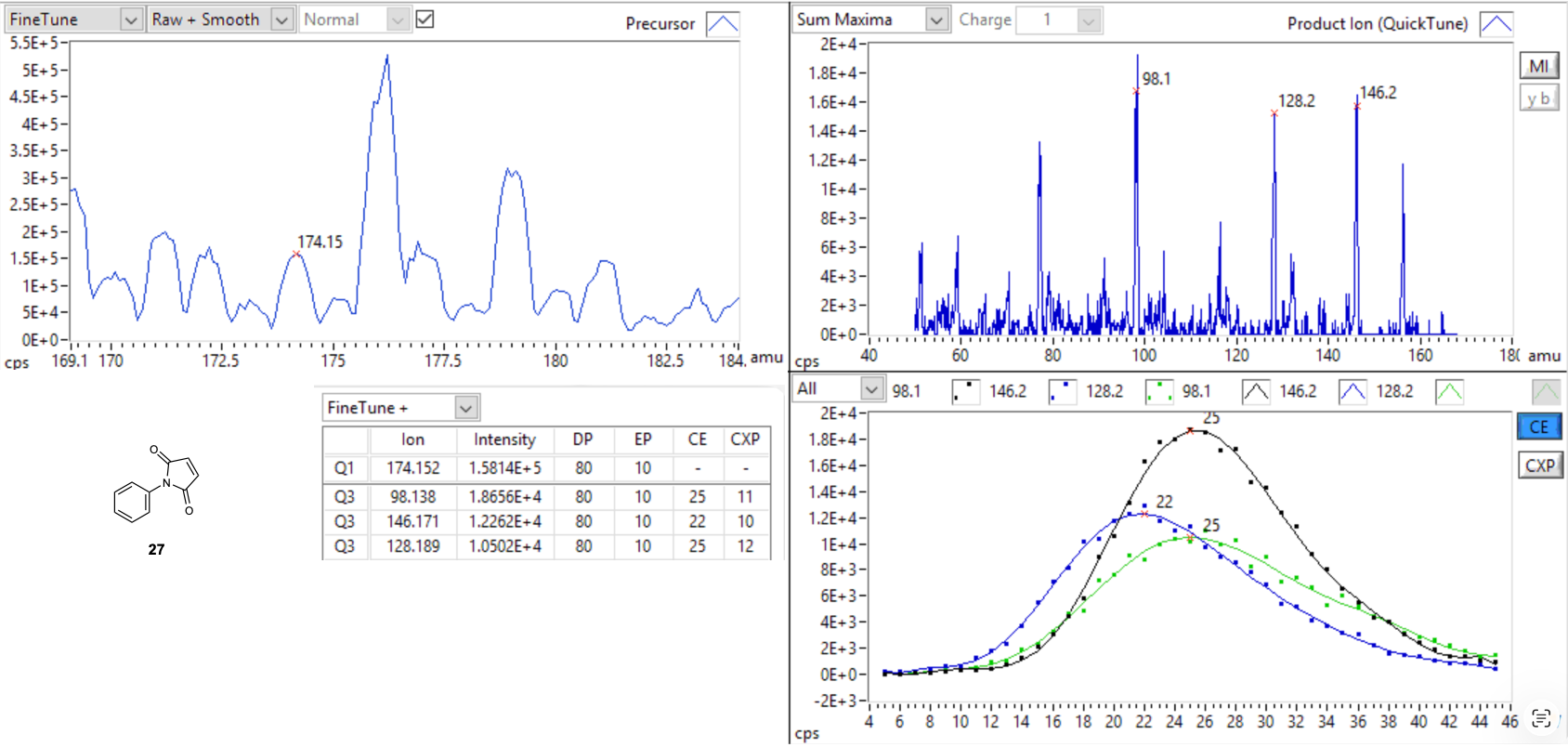
**

**
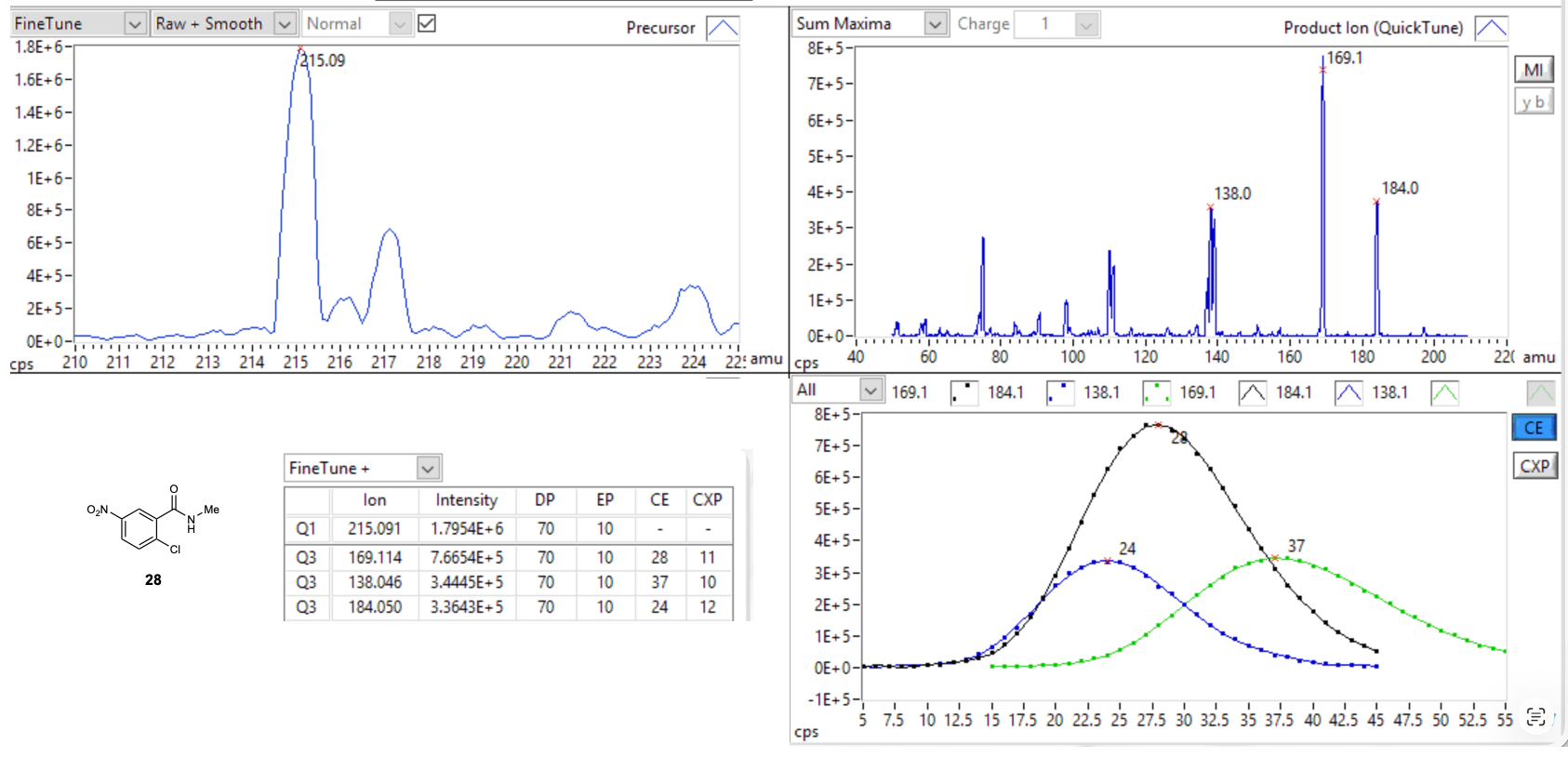
**

**
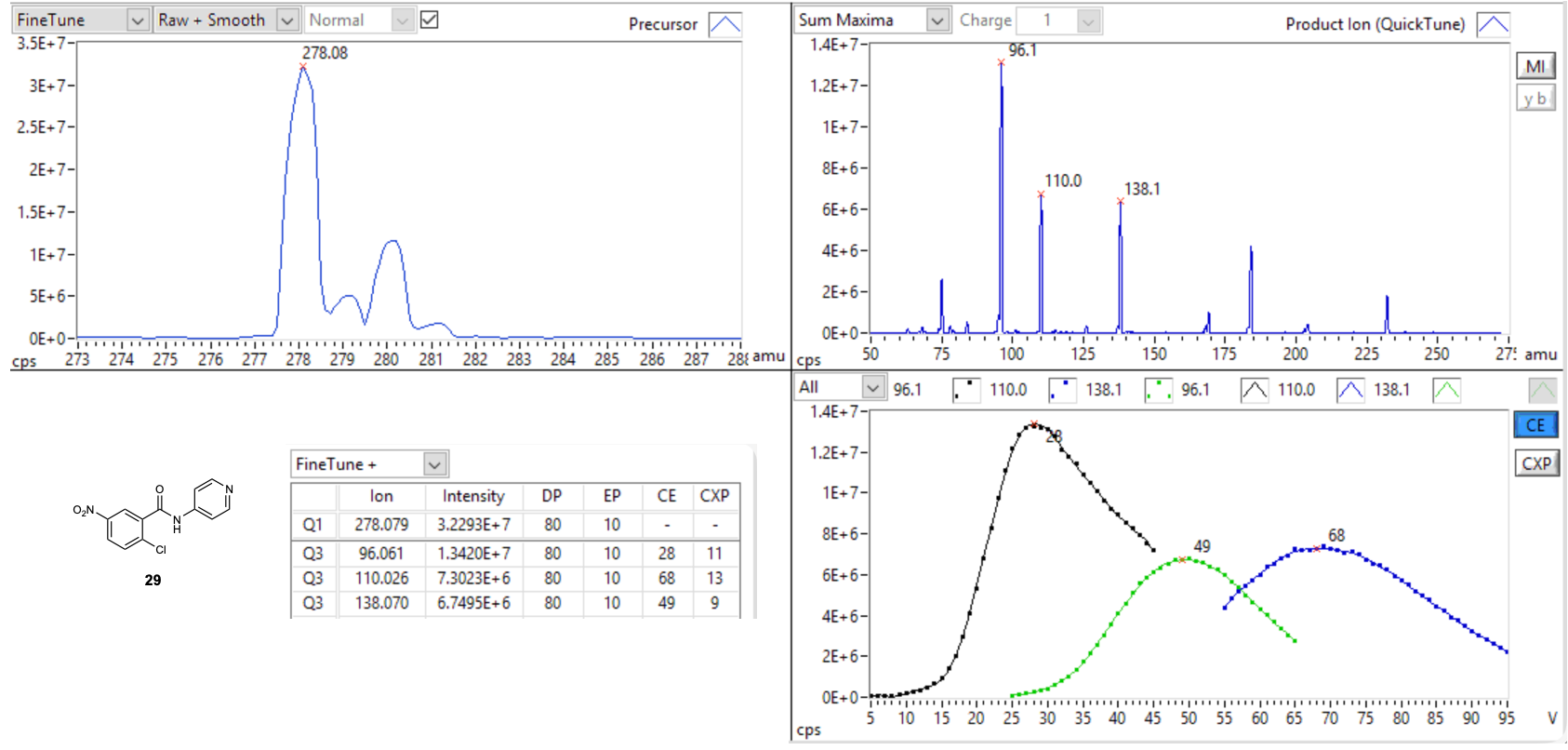
**

**
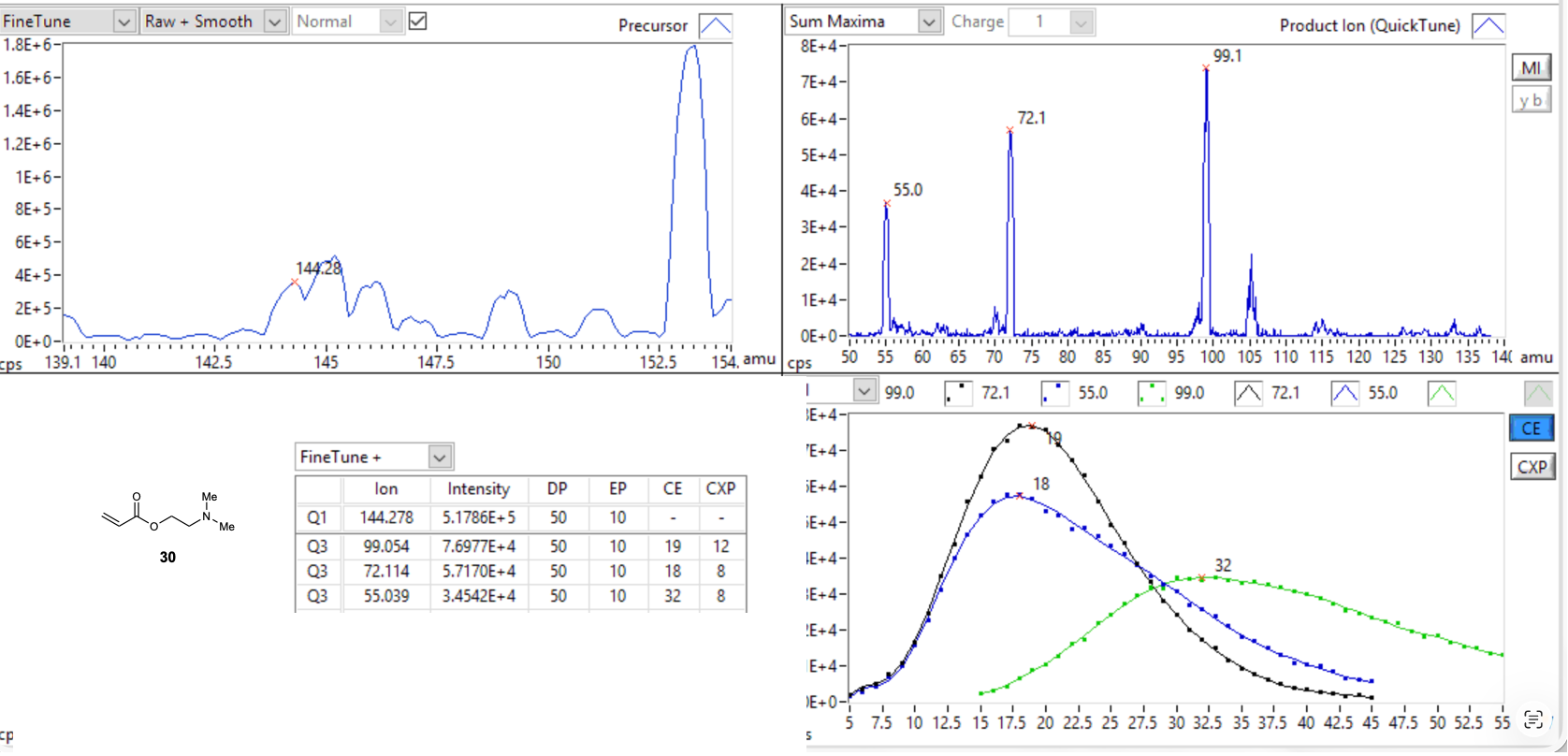
**

**
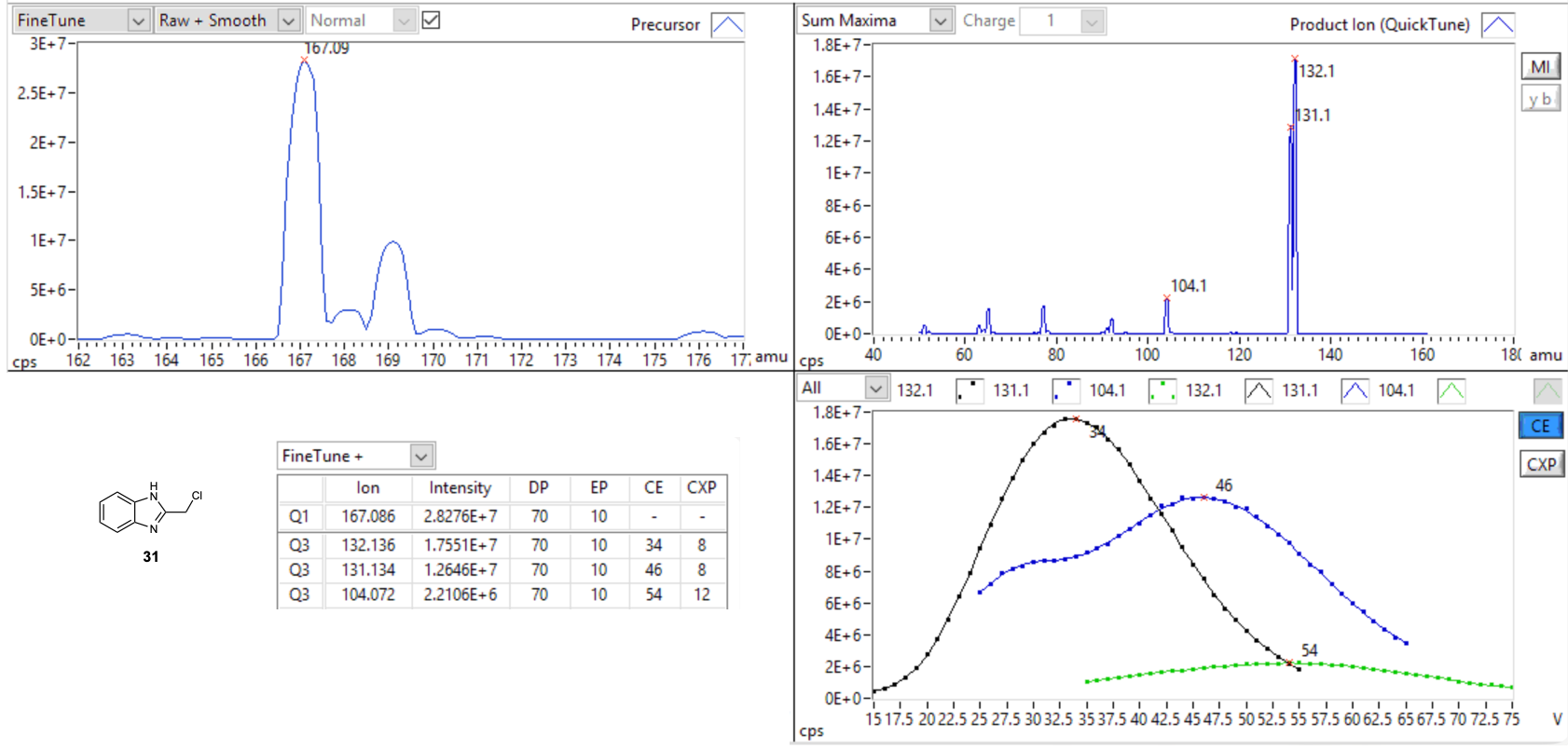
**

**
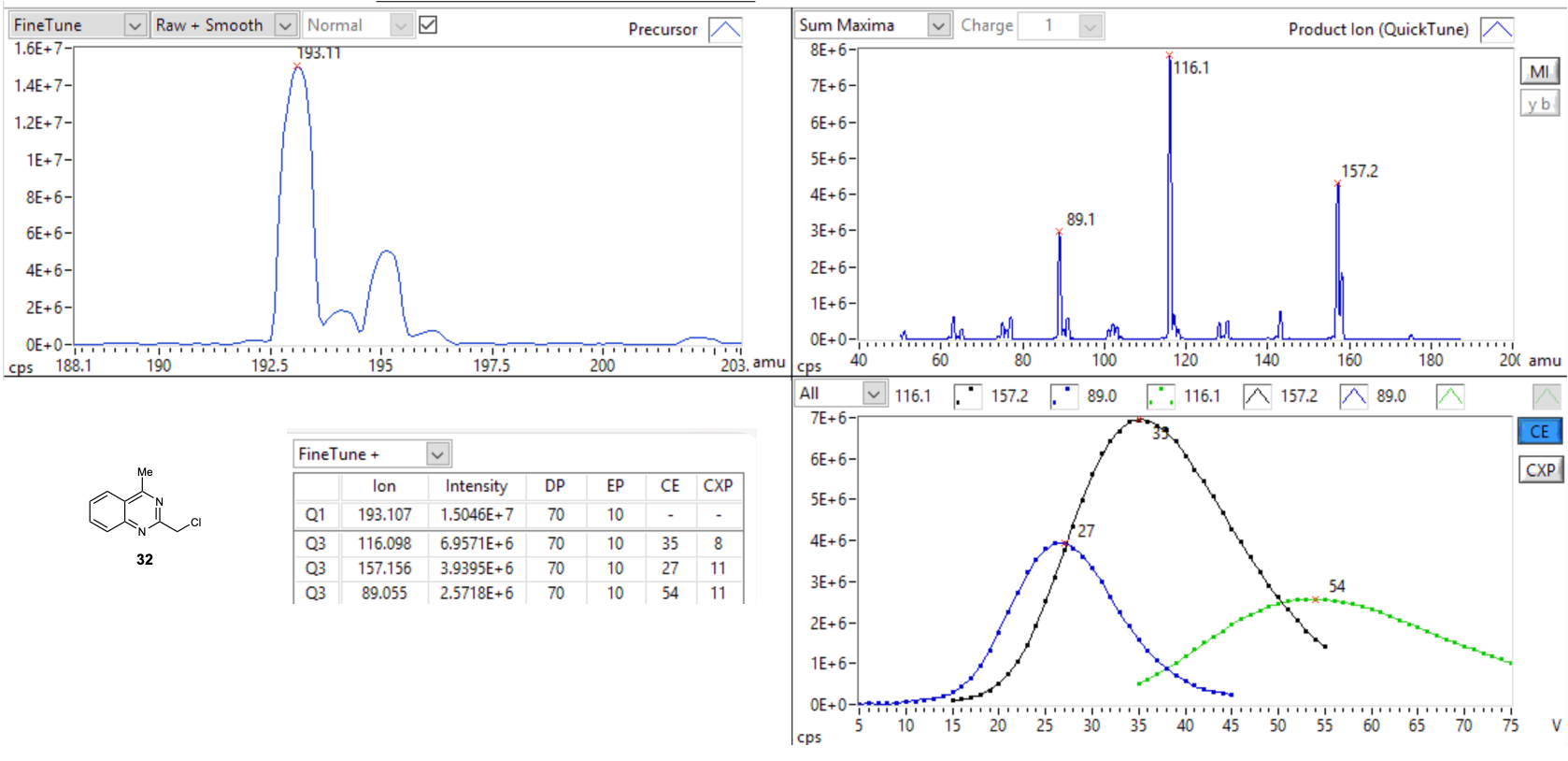
**

**
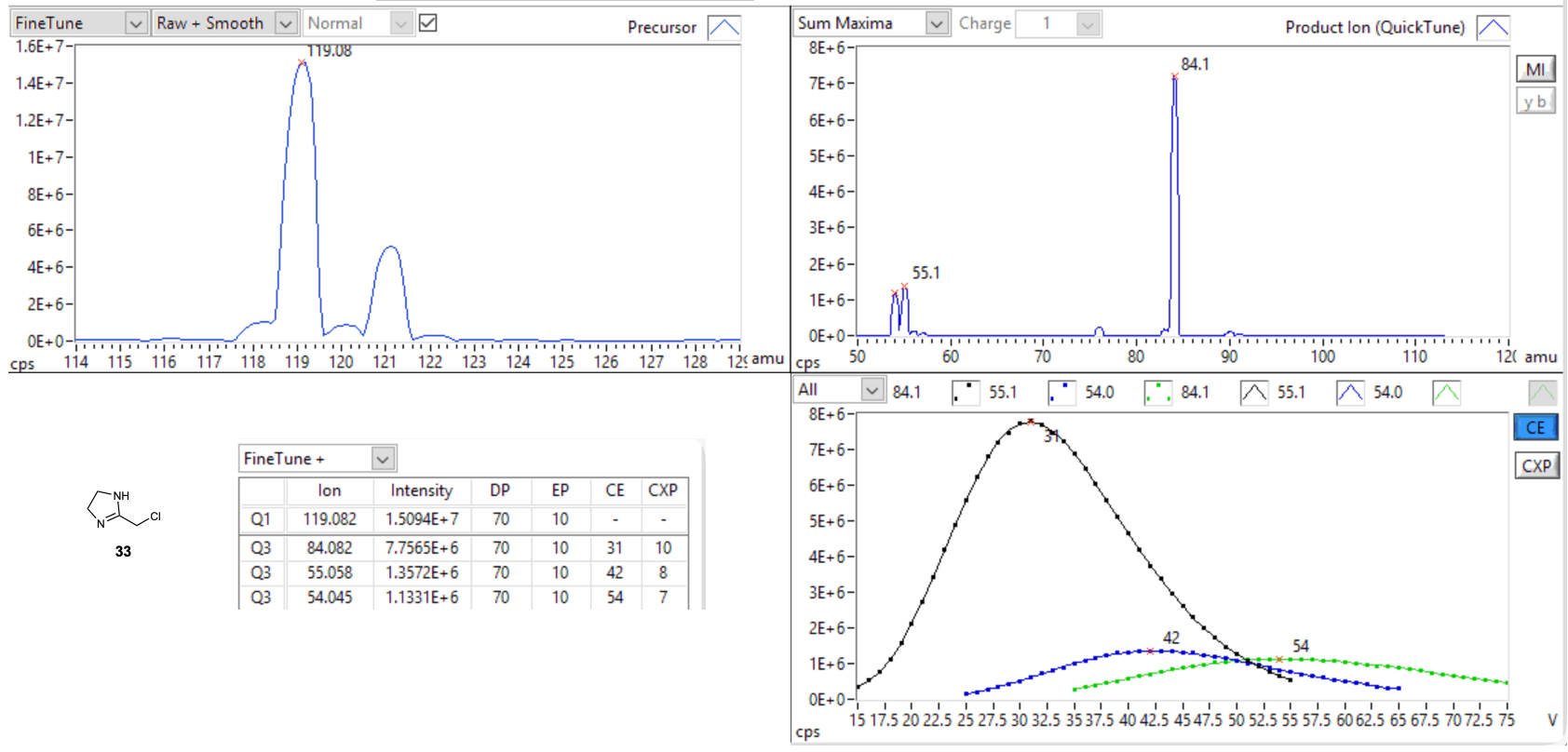
**

**
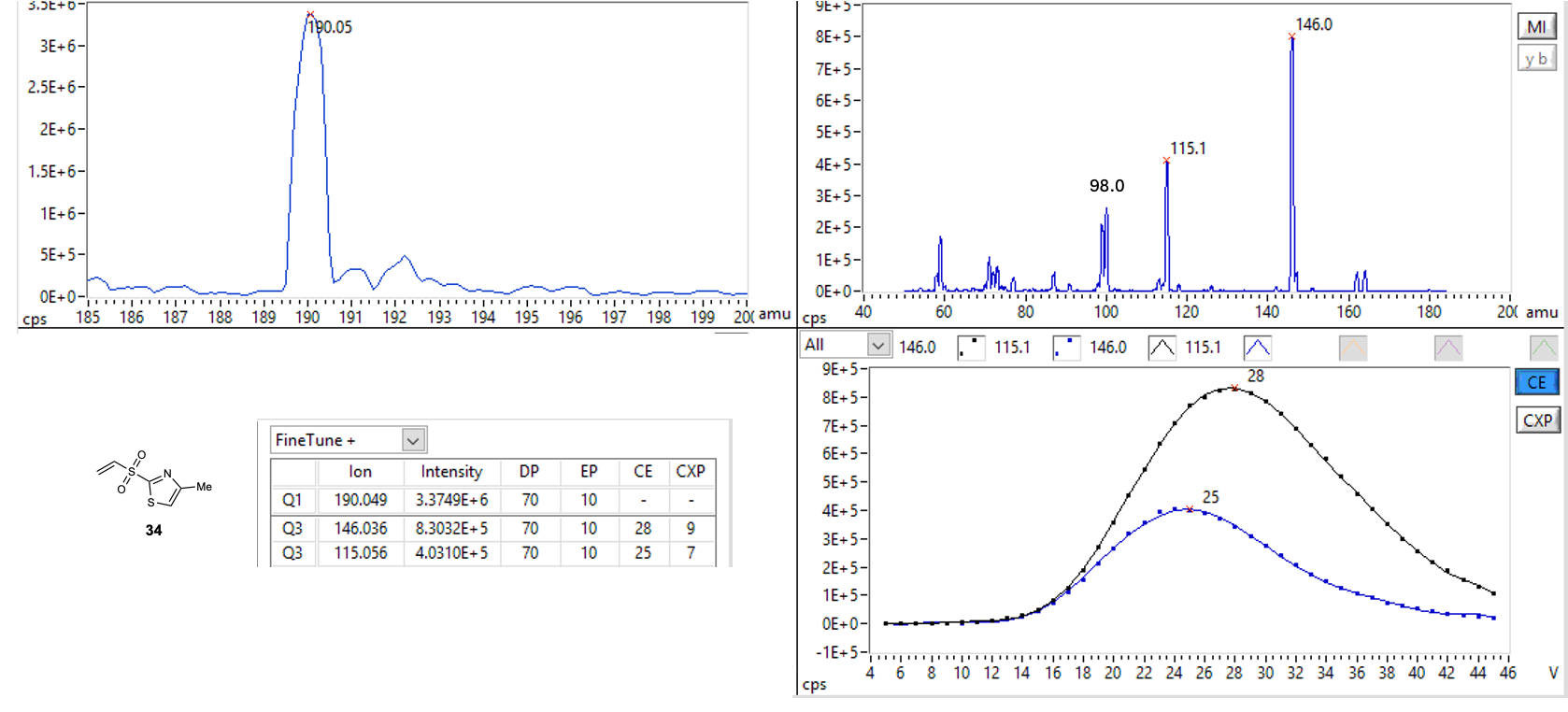
**

**
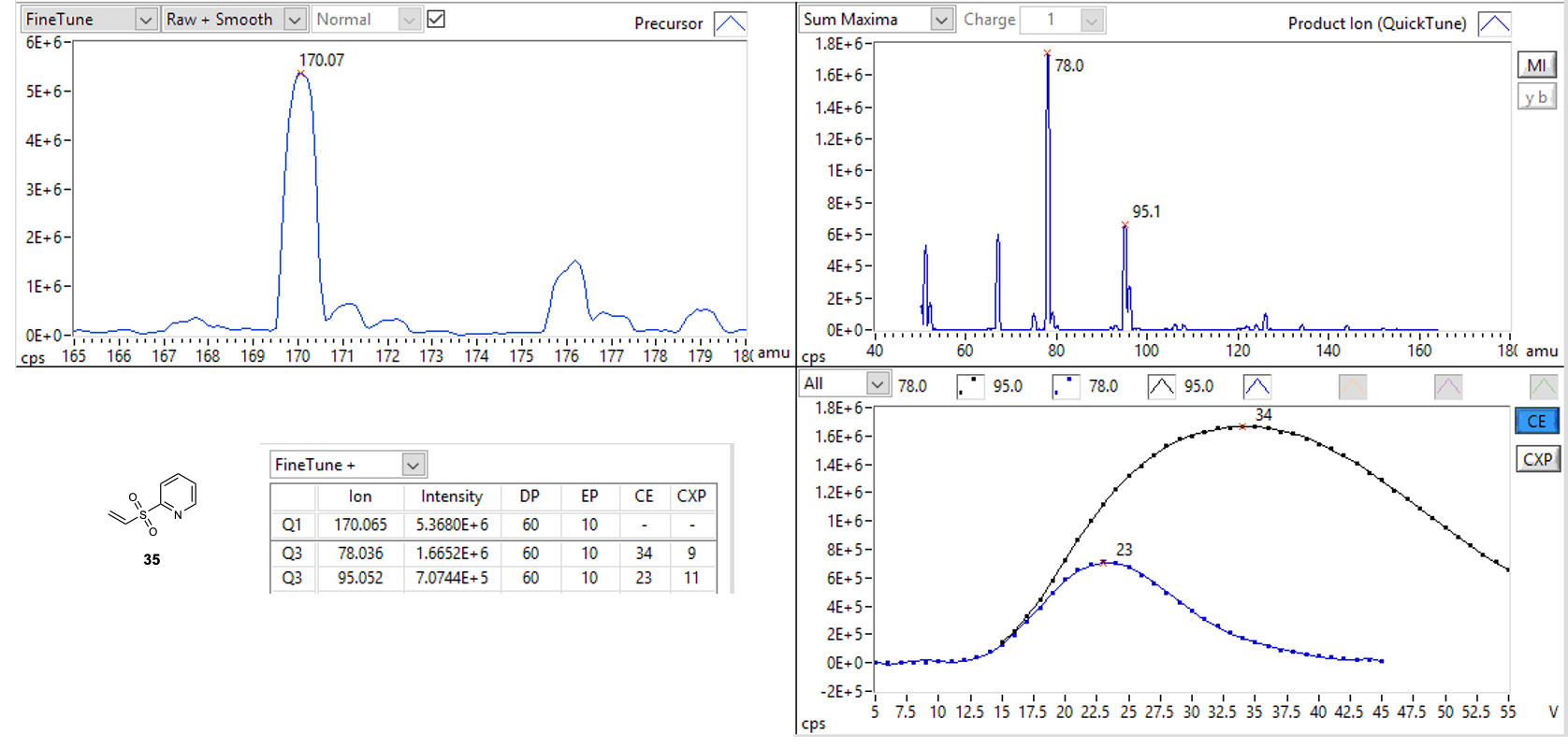
**

**
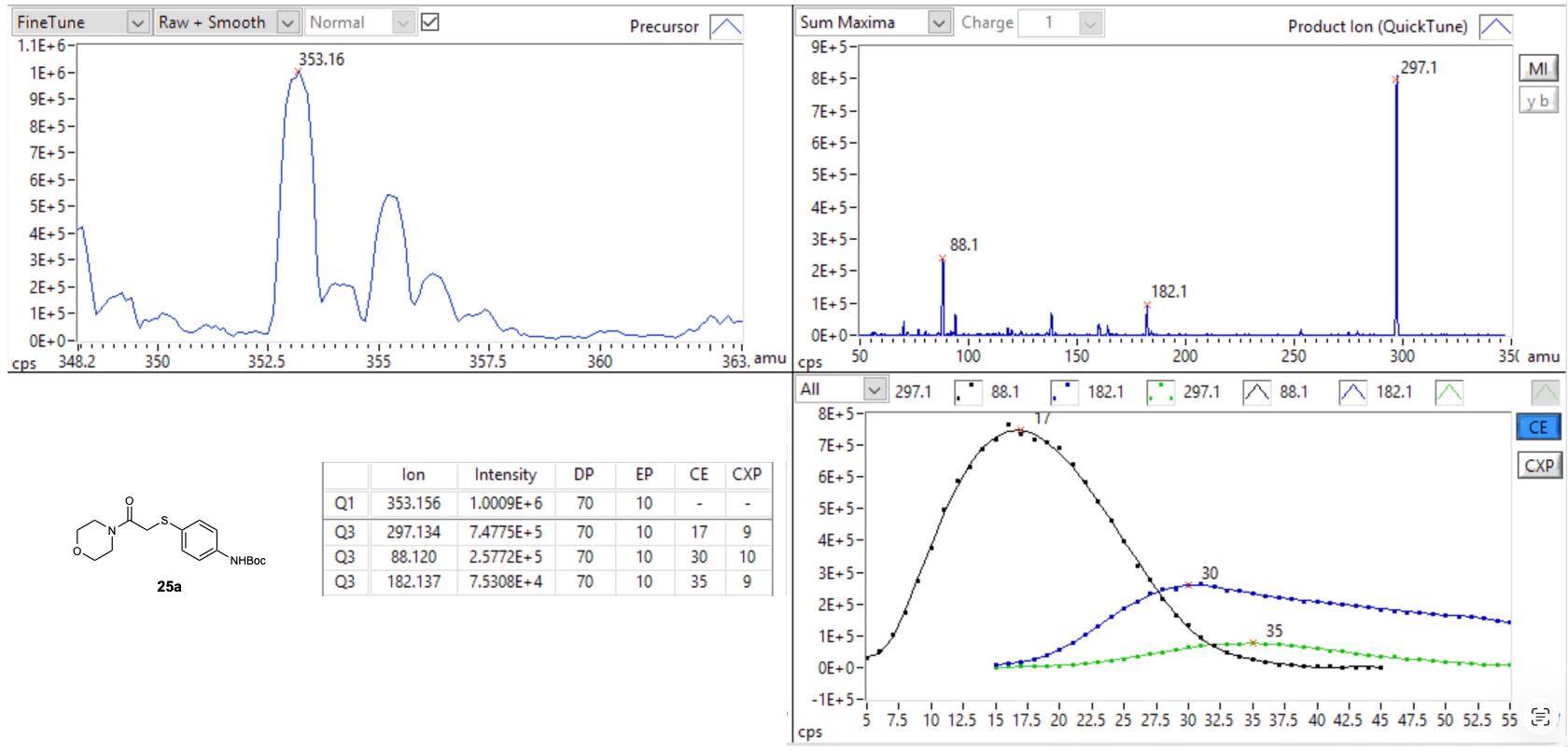
**

**
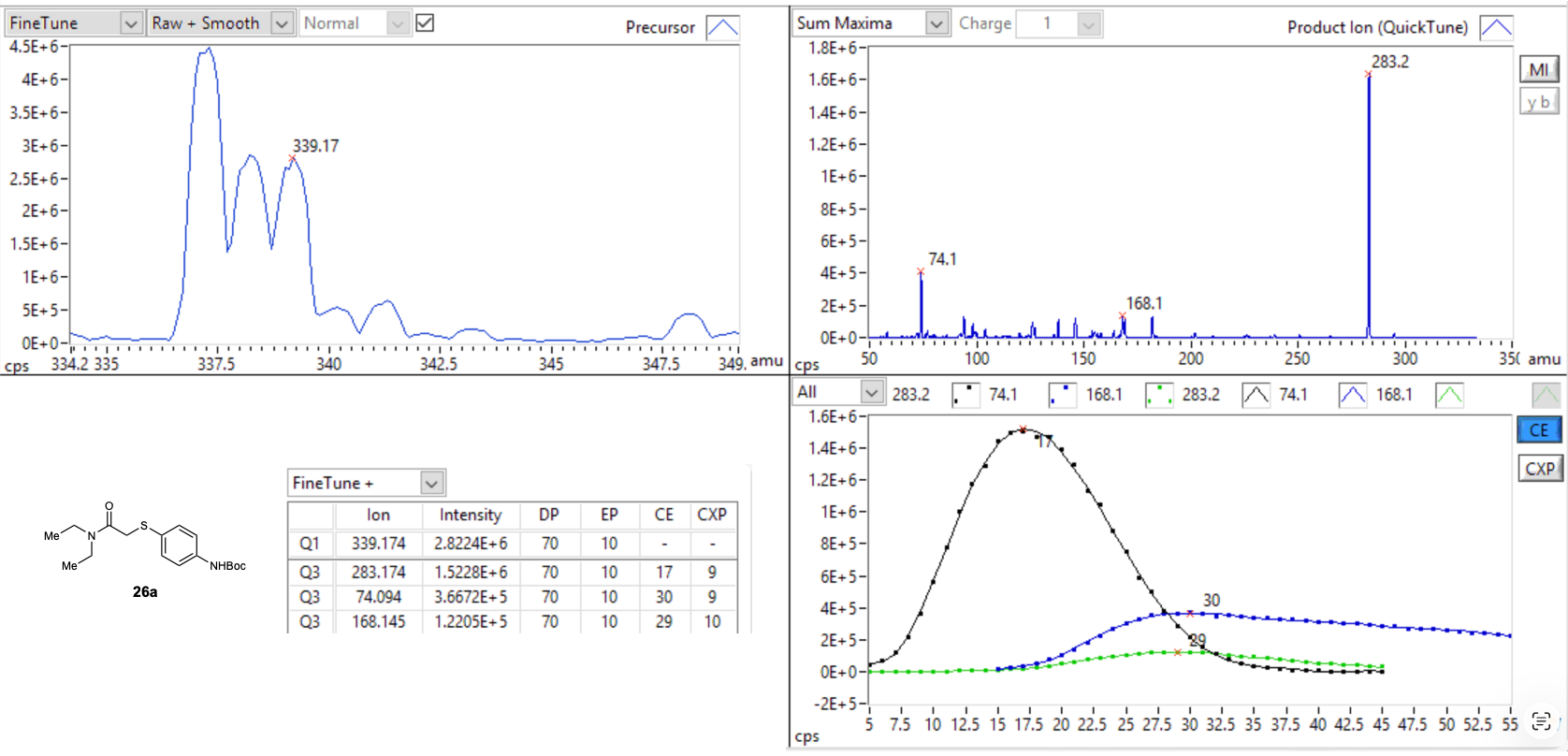
**

**
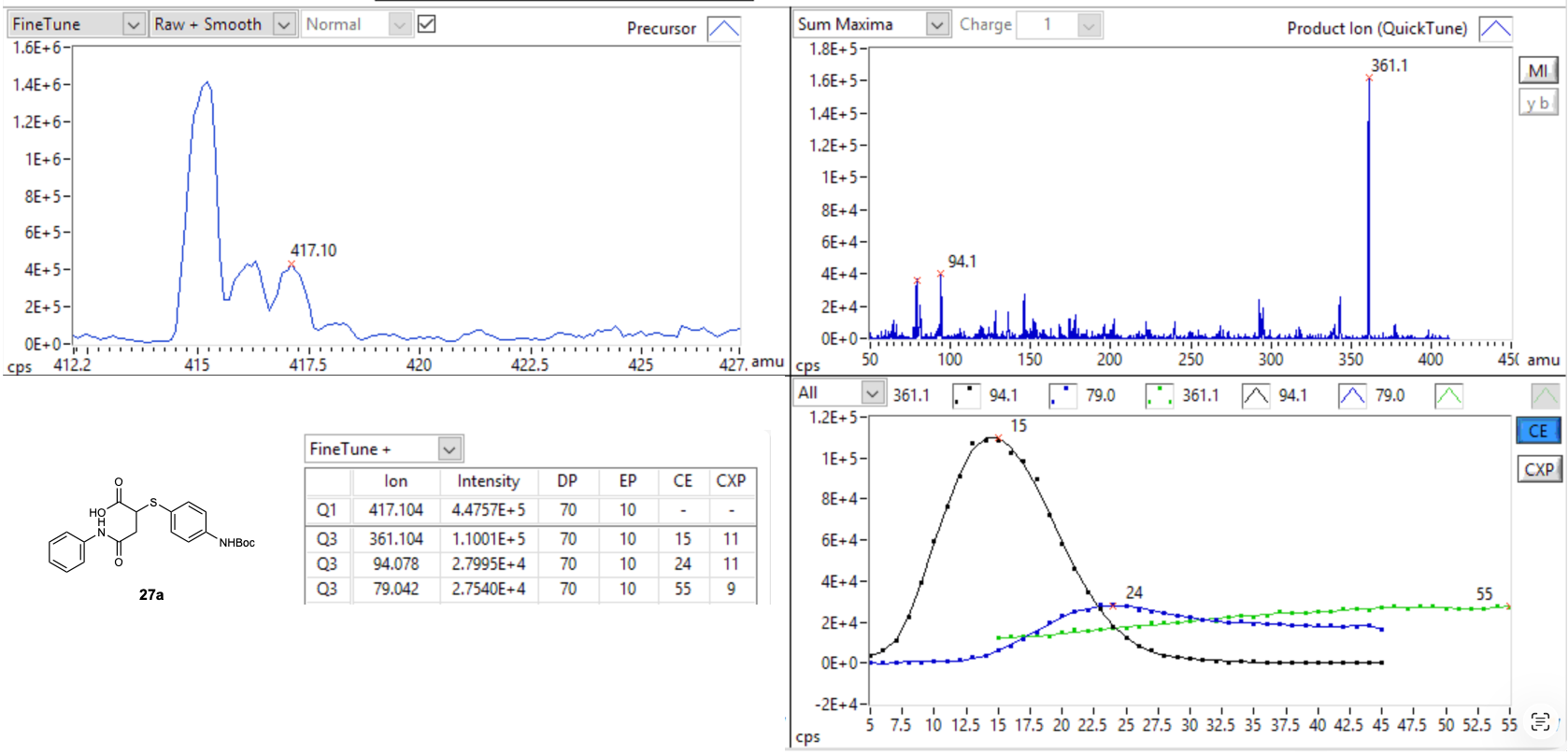
**

**
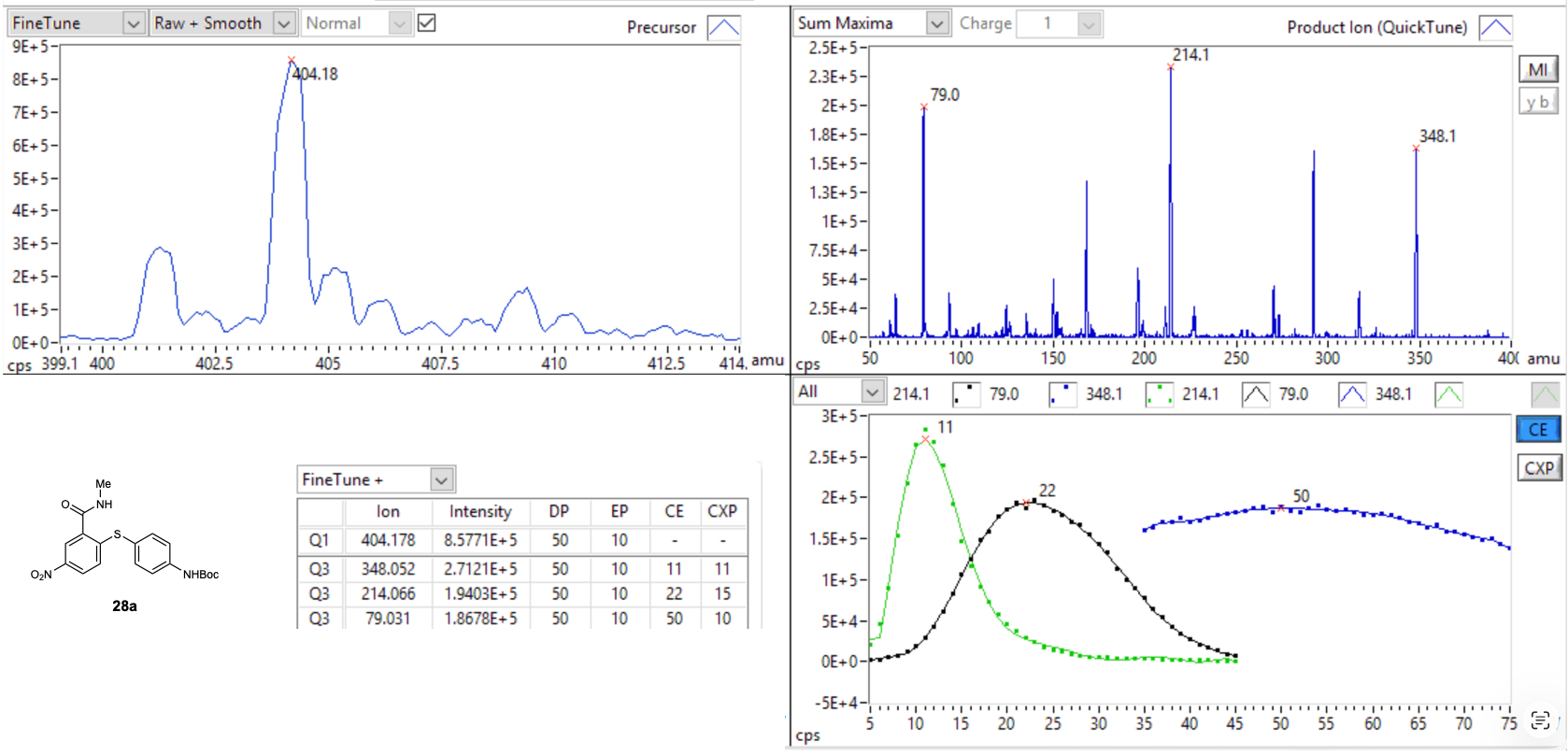
**

**
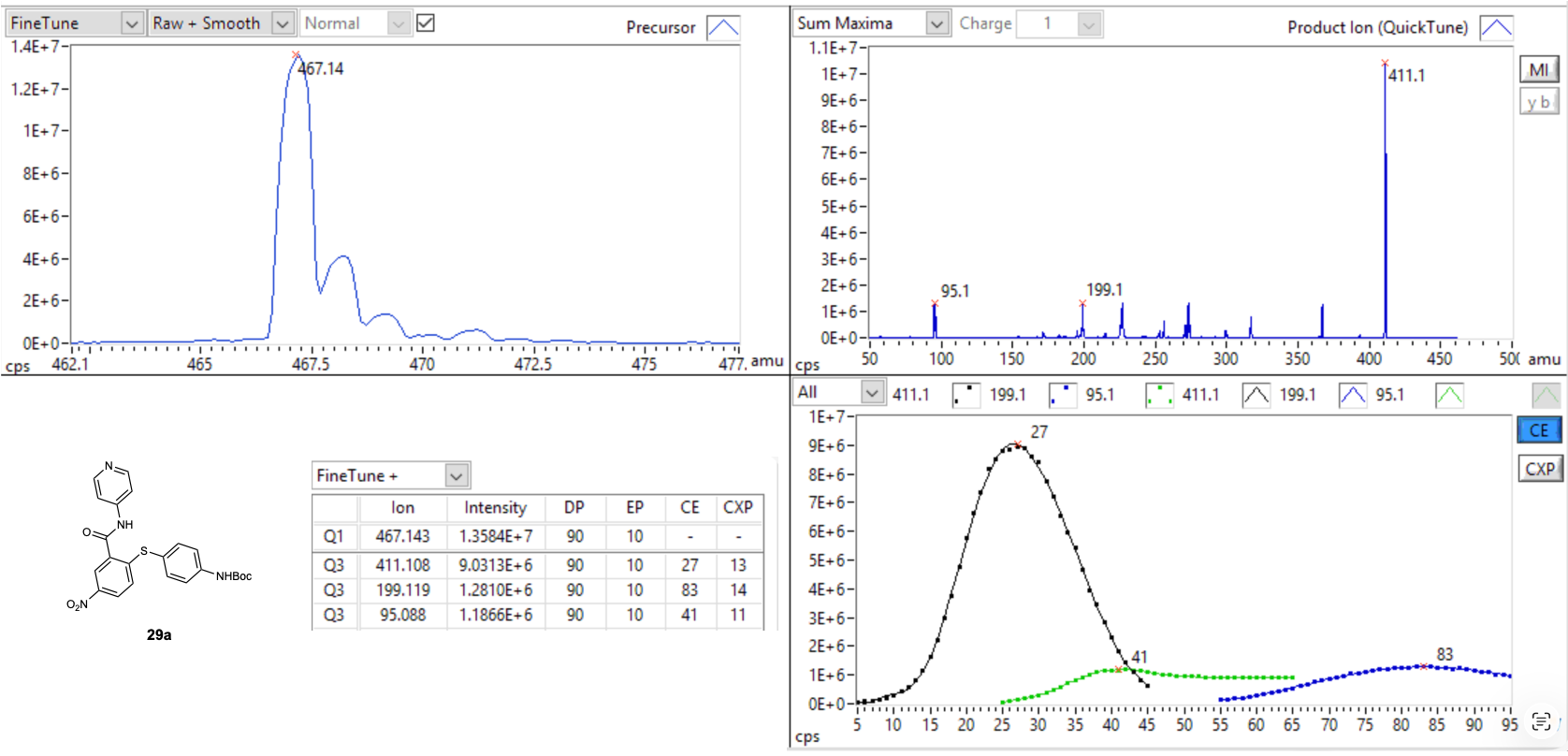
**

**
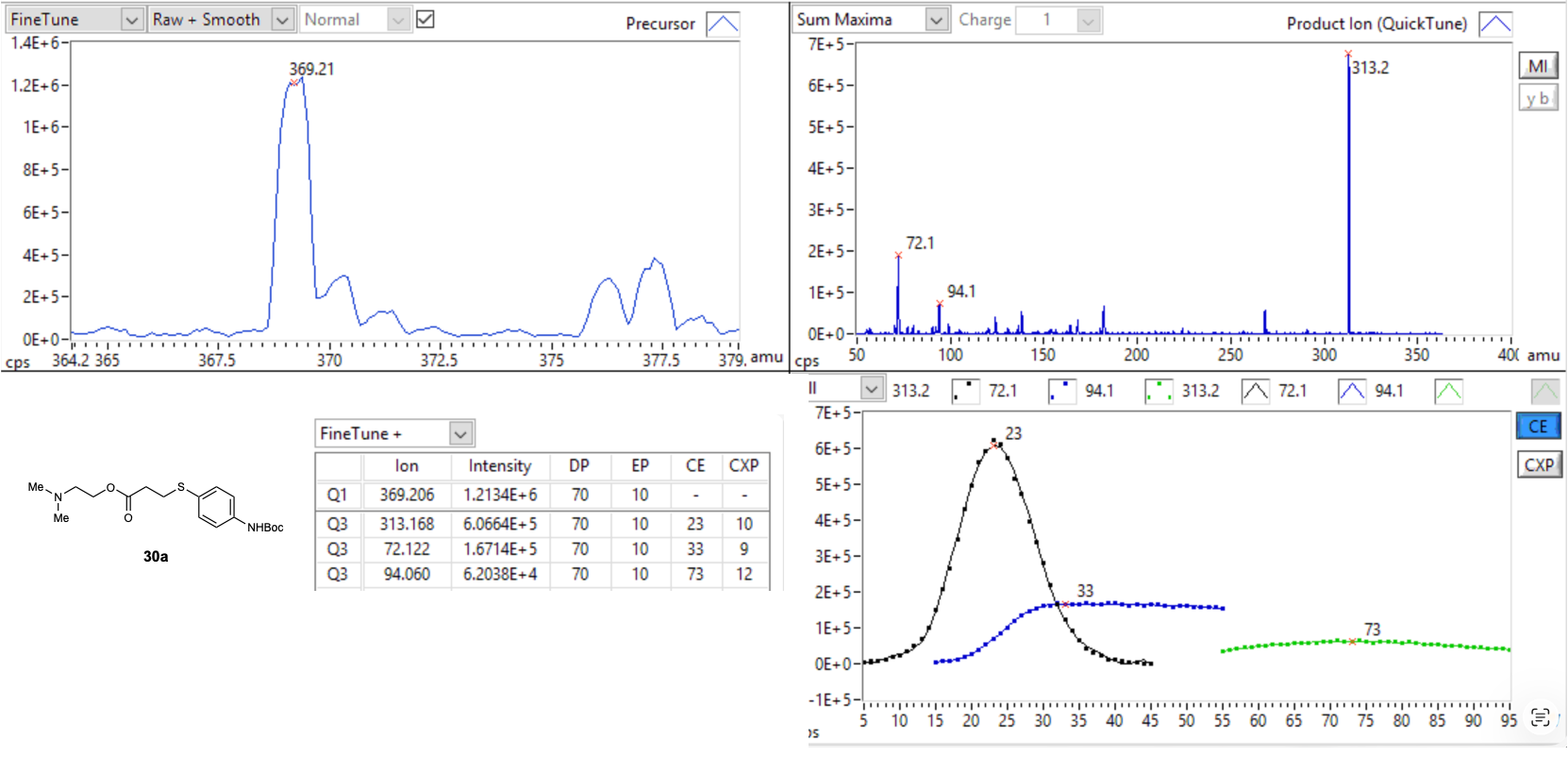
**

**
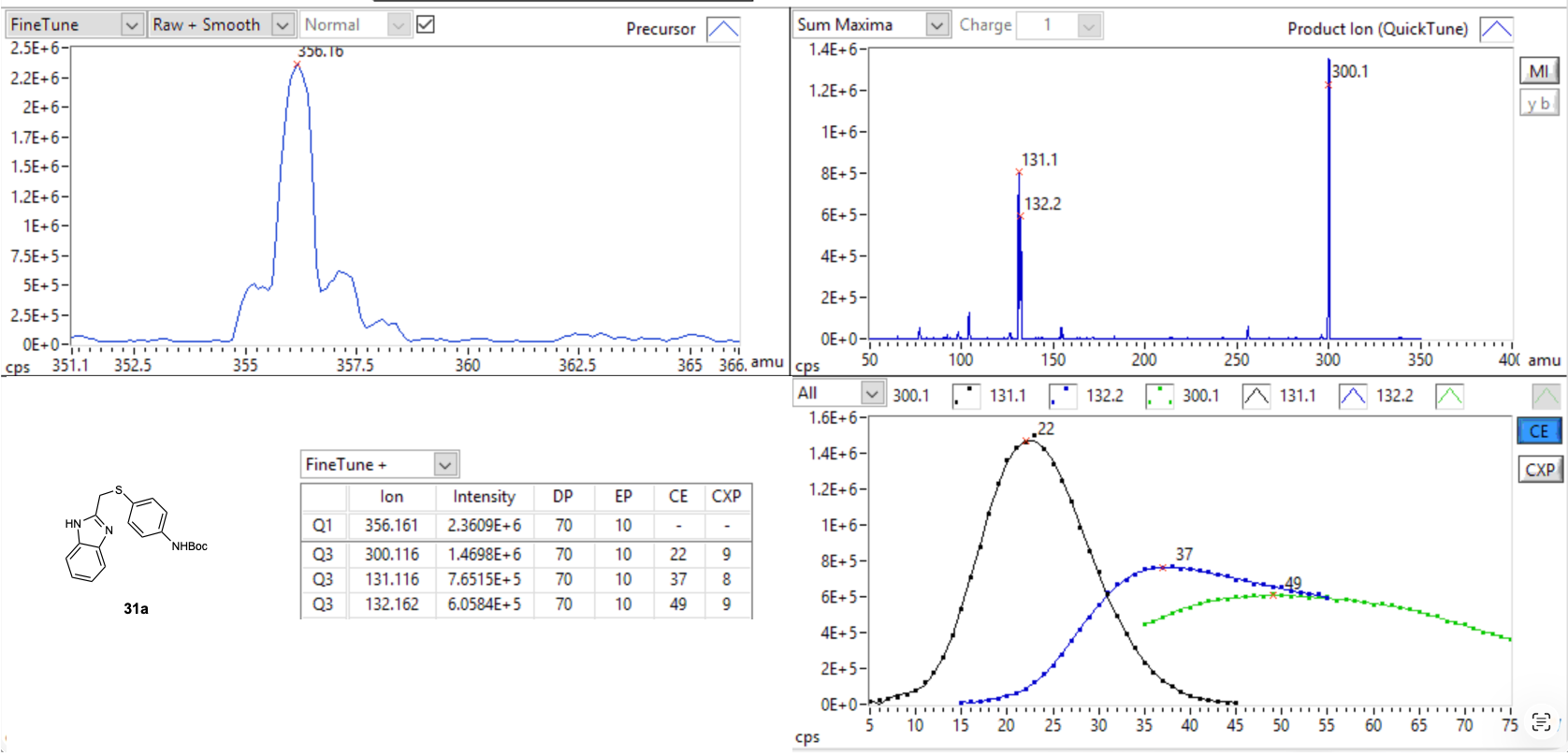
**

**
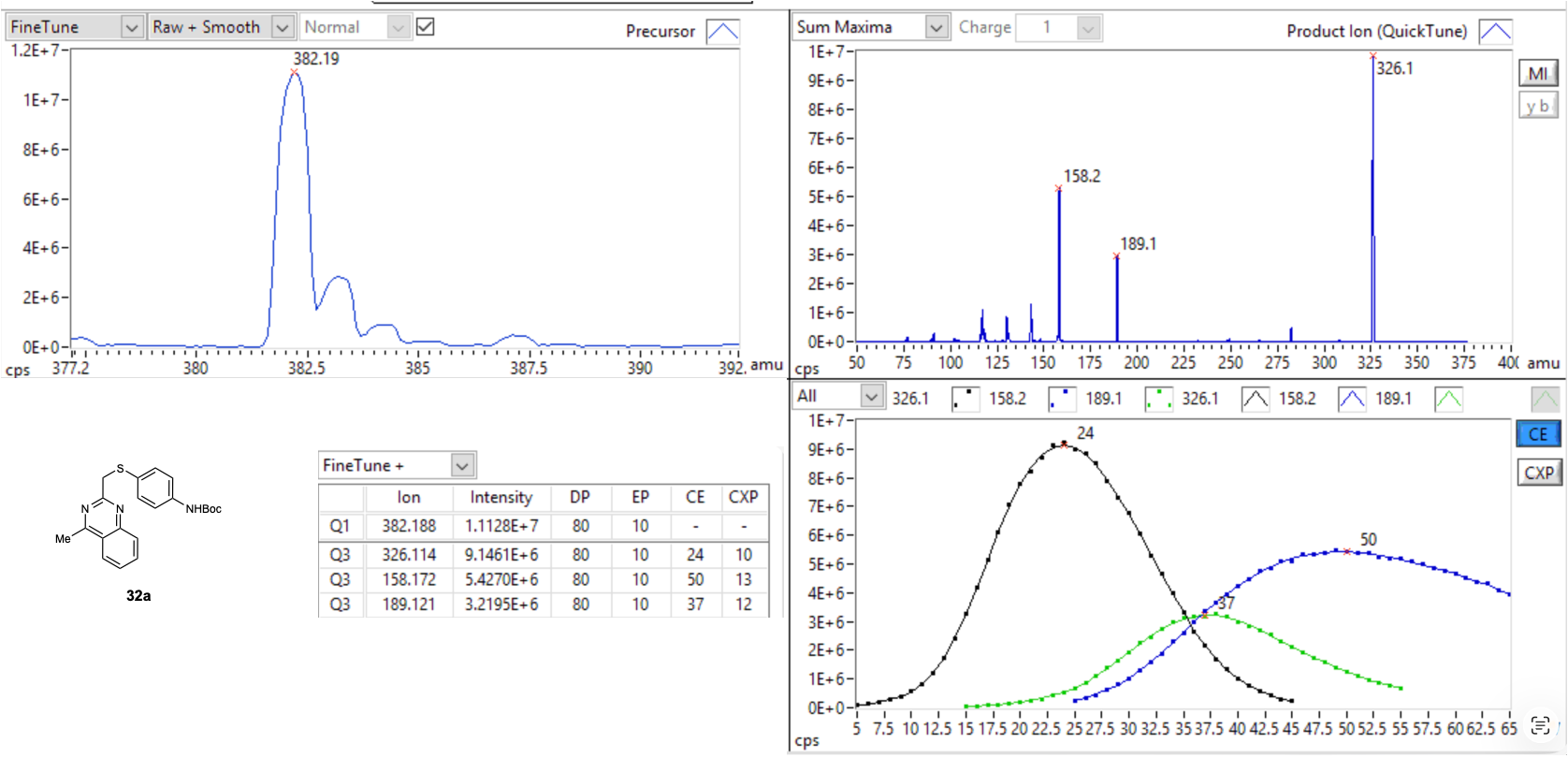
**

**
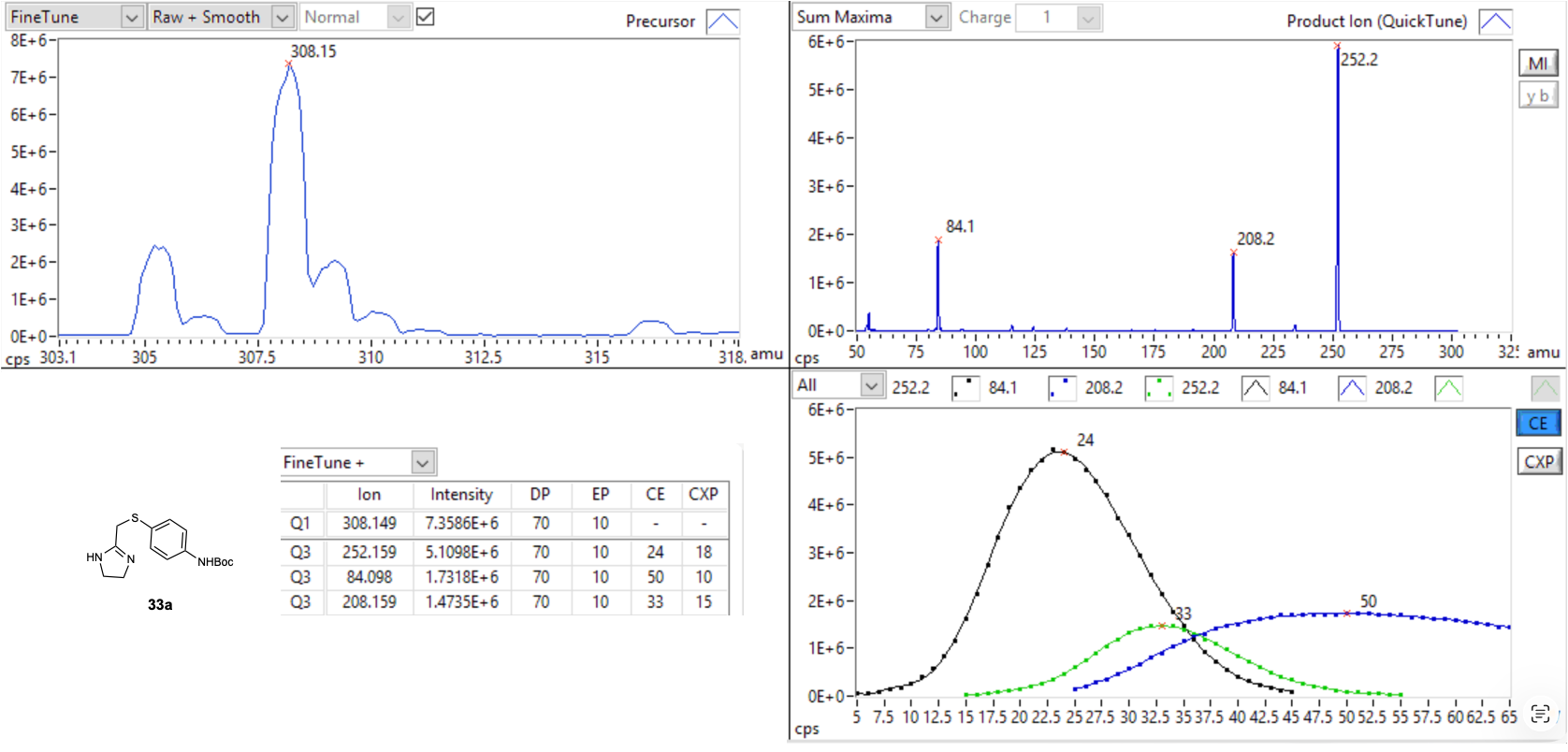
**

**
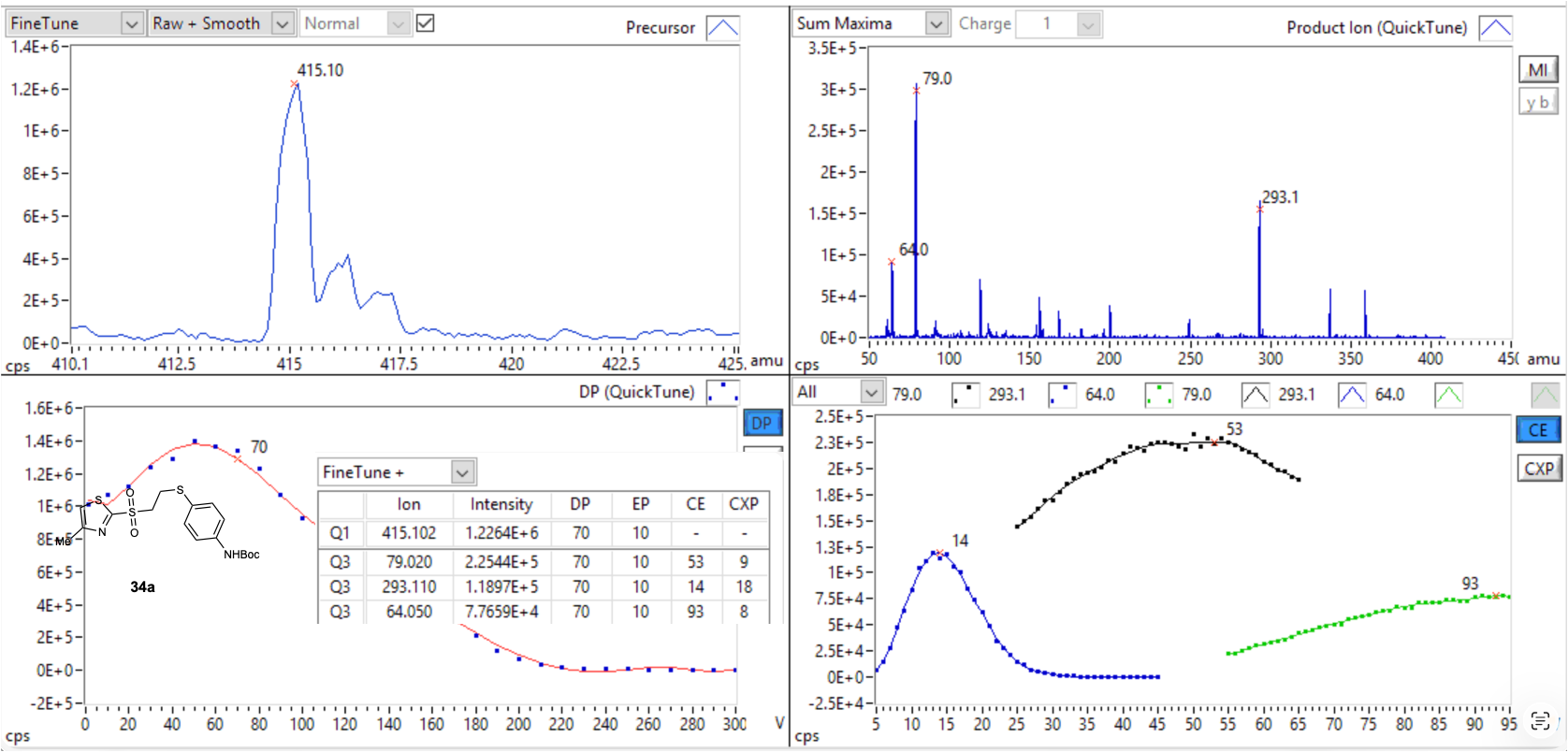
**

**
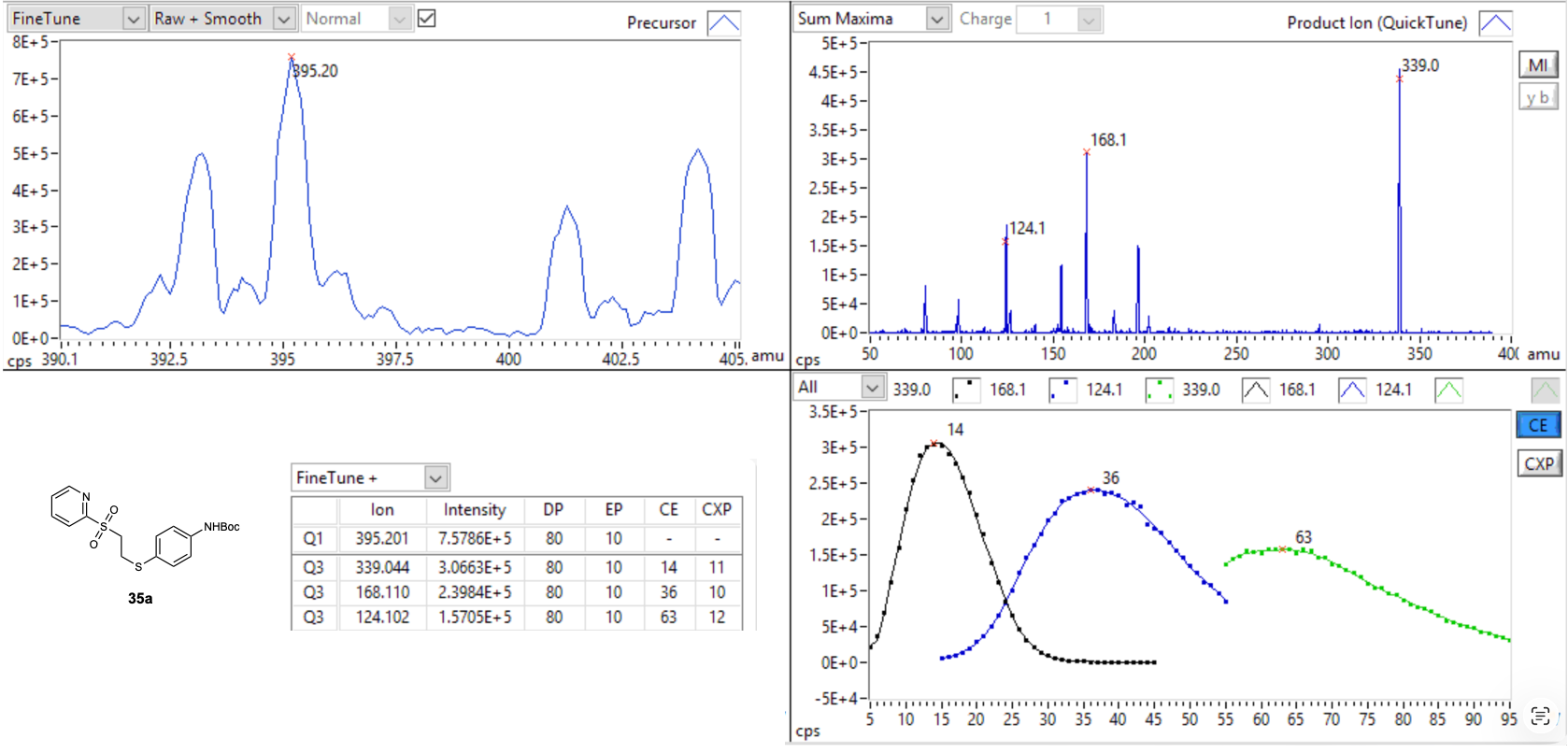
**

**
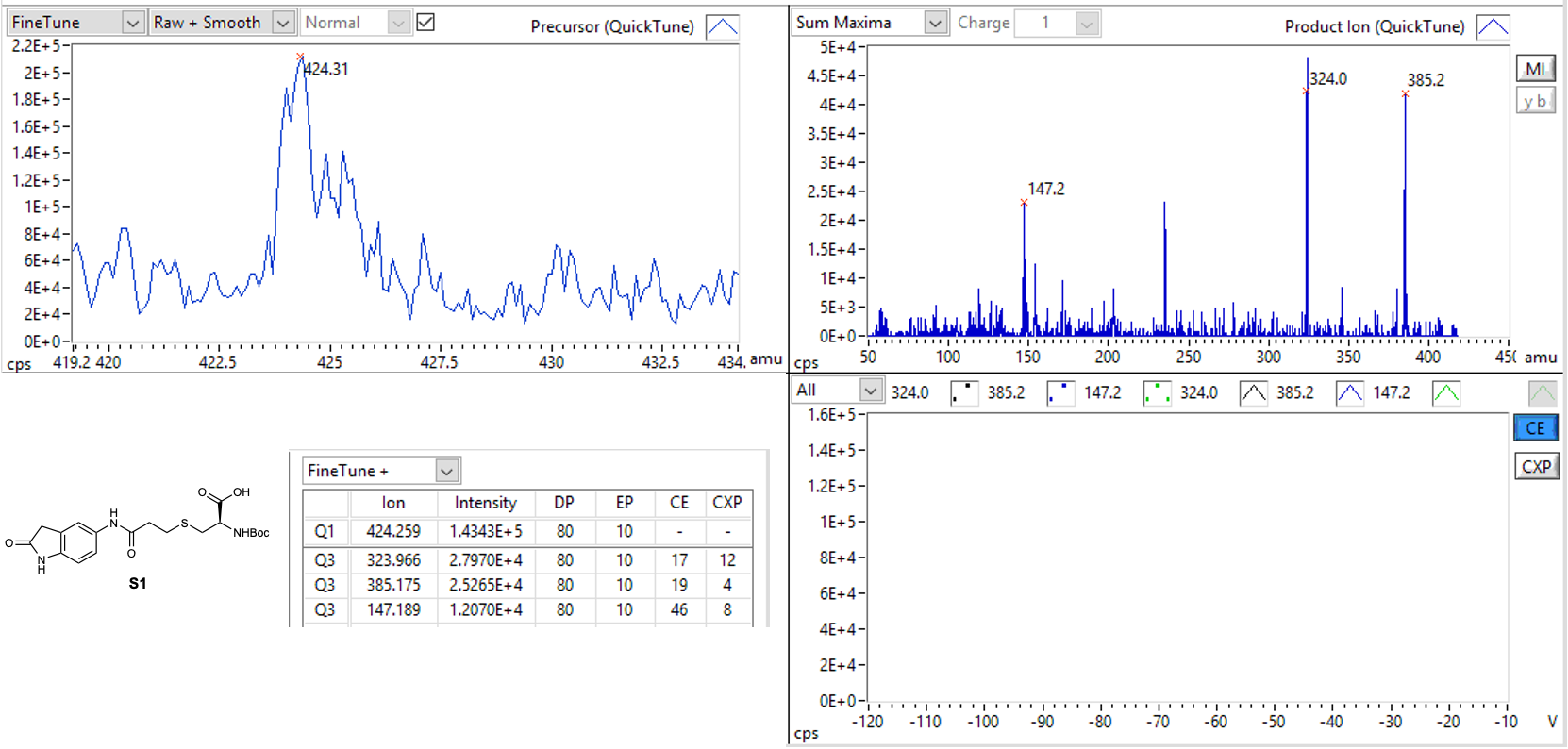
**

**
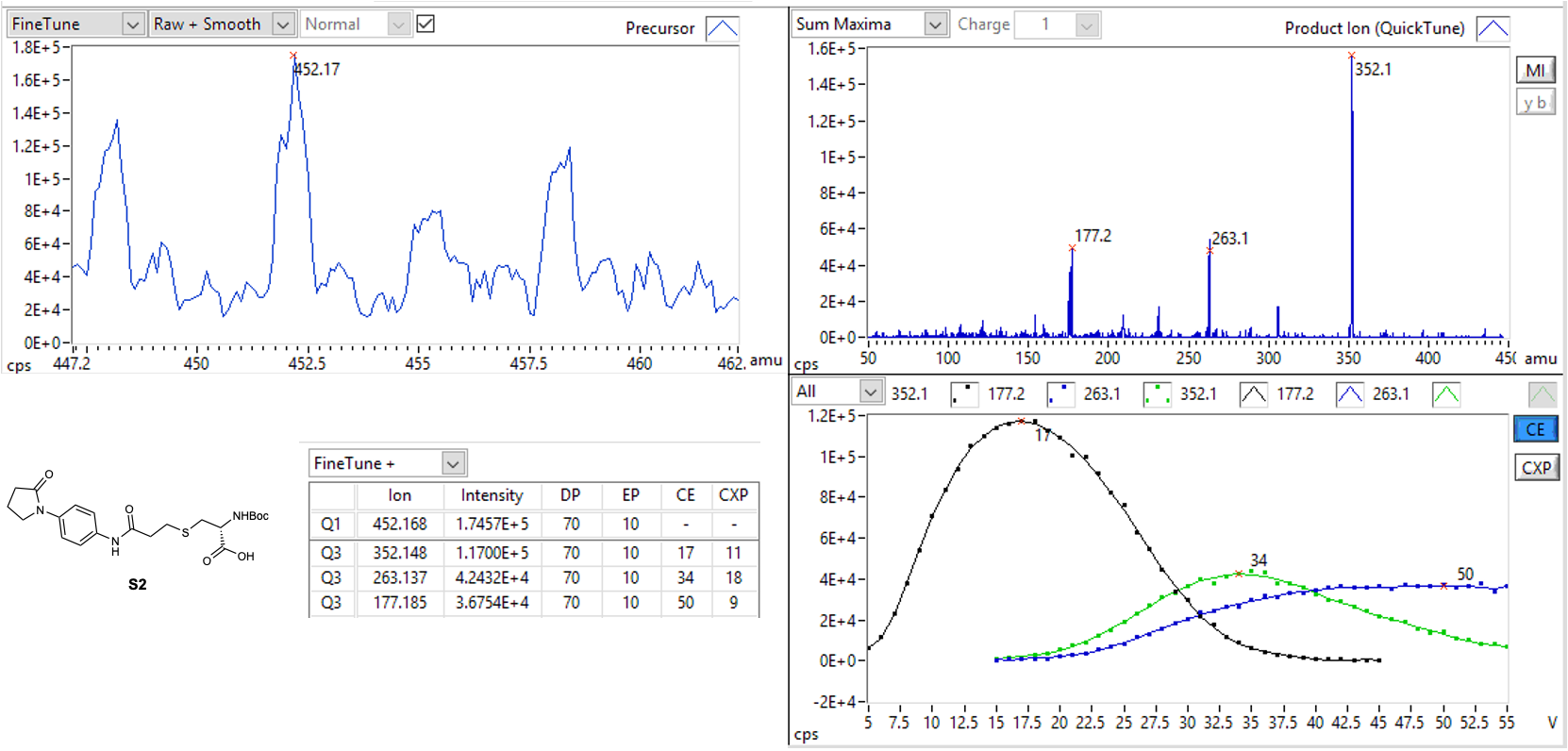
**

**
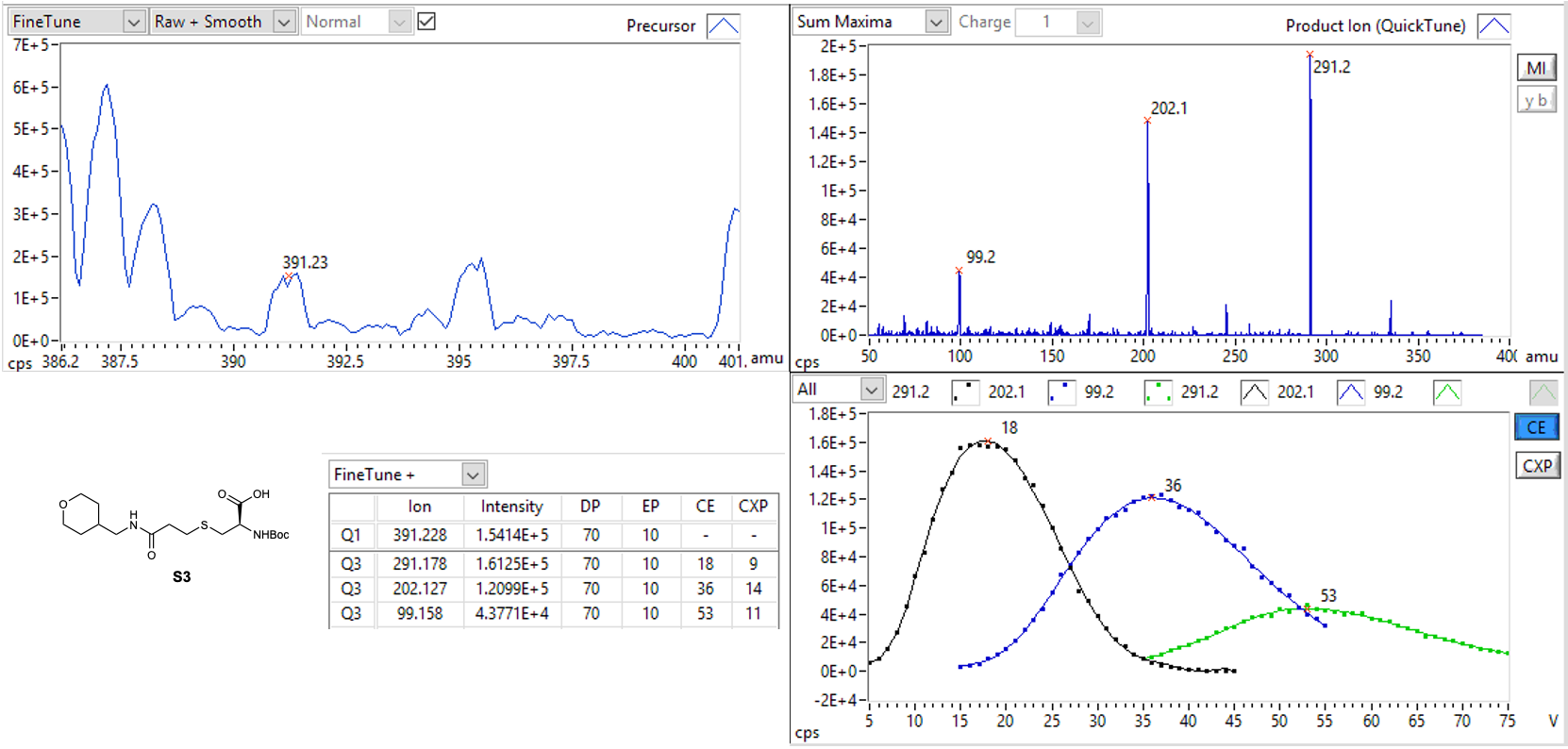
**

**
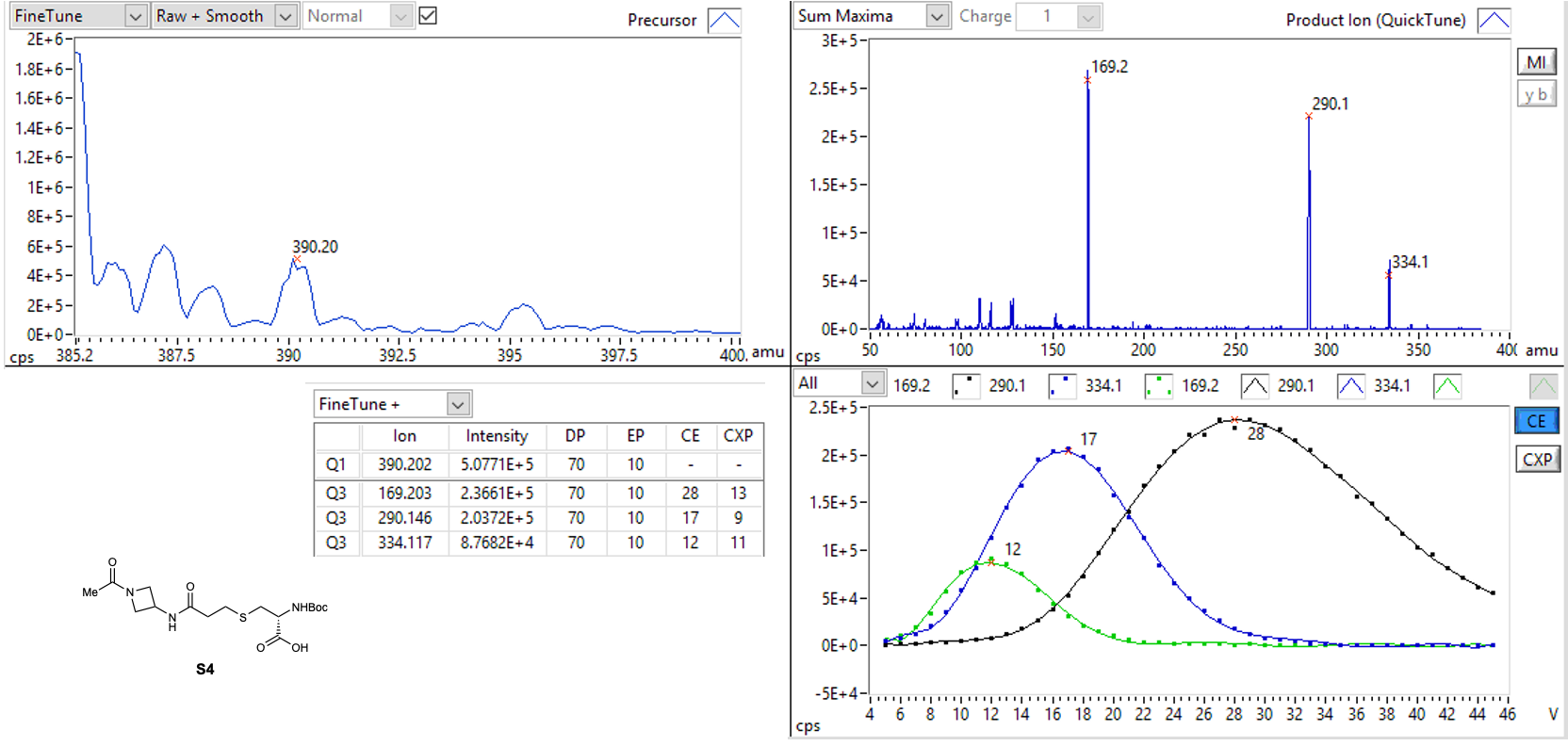
**

**
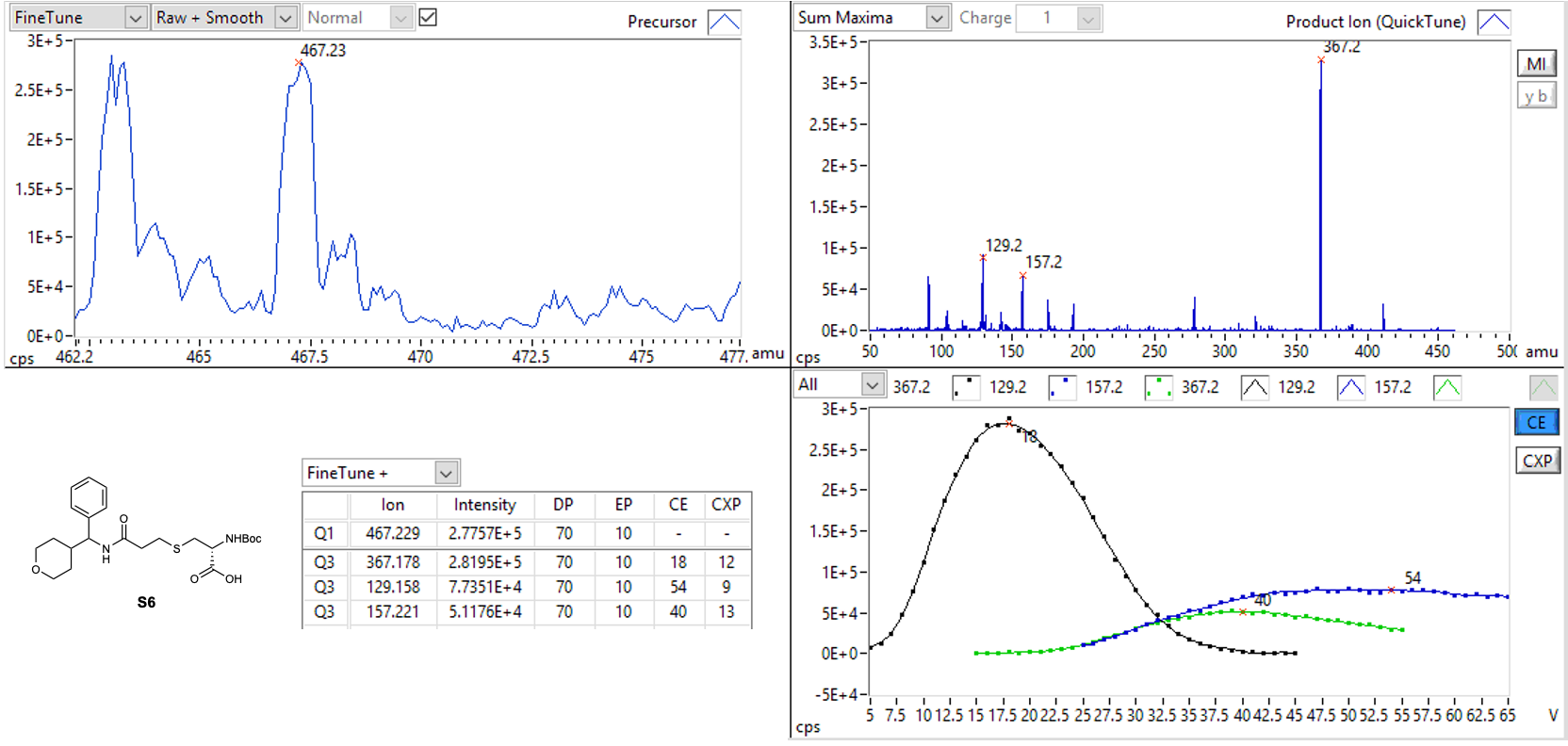
**

**
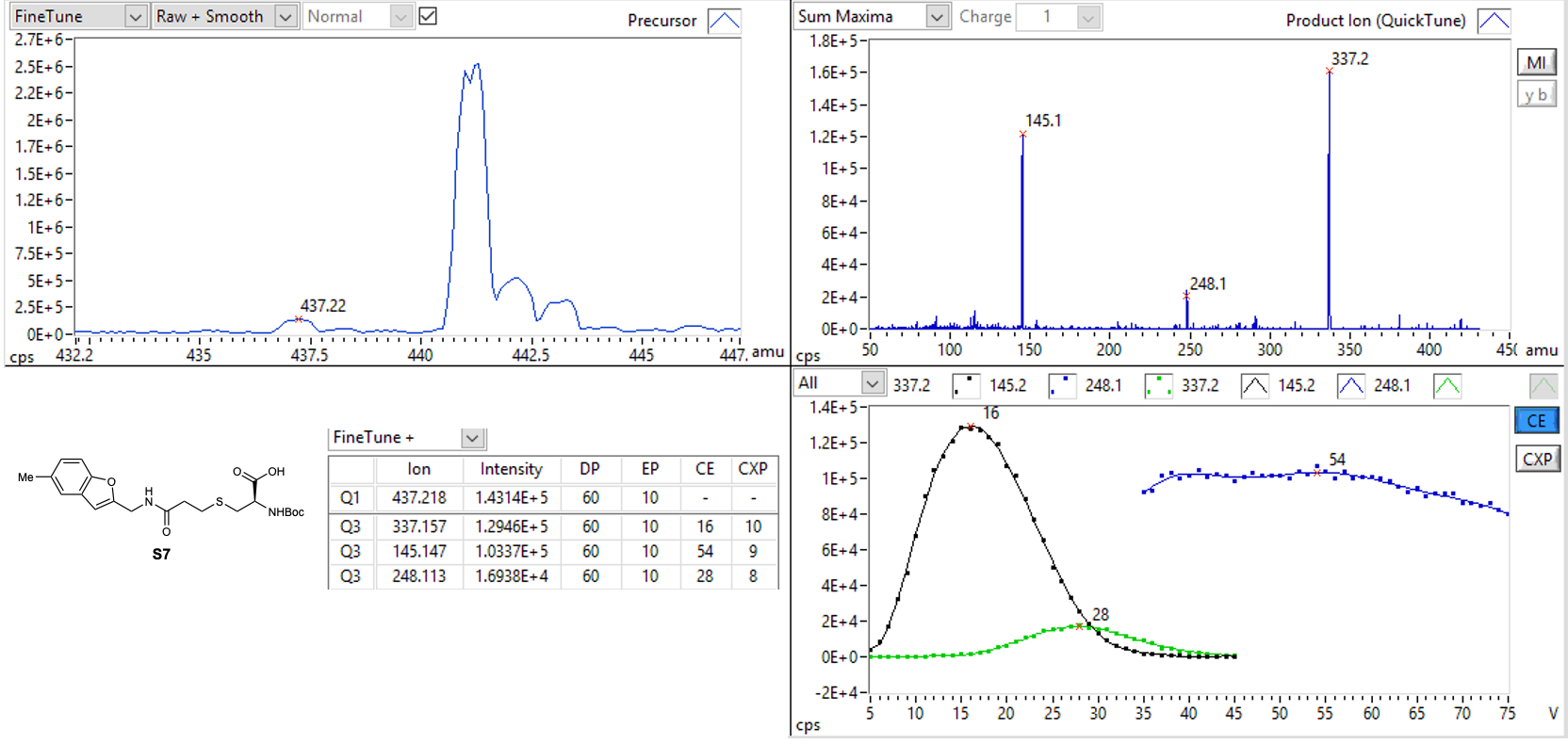
**

**
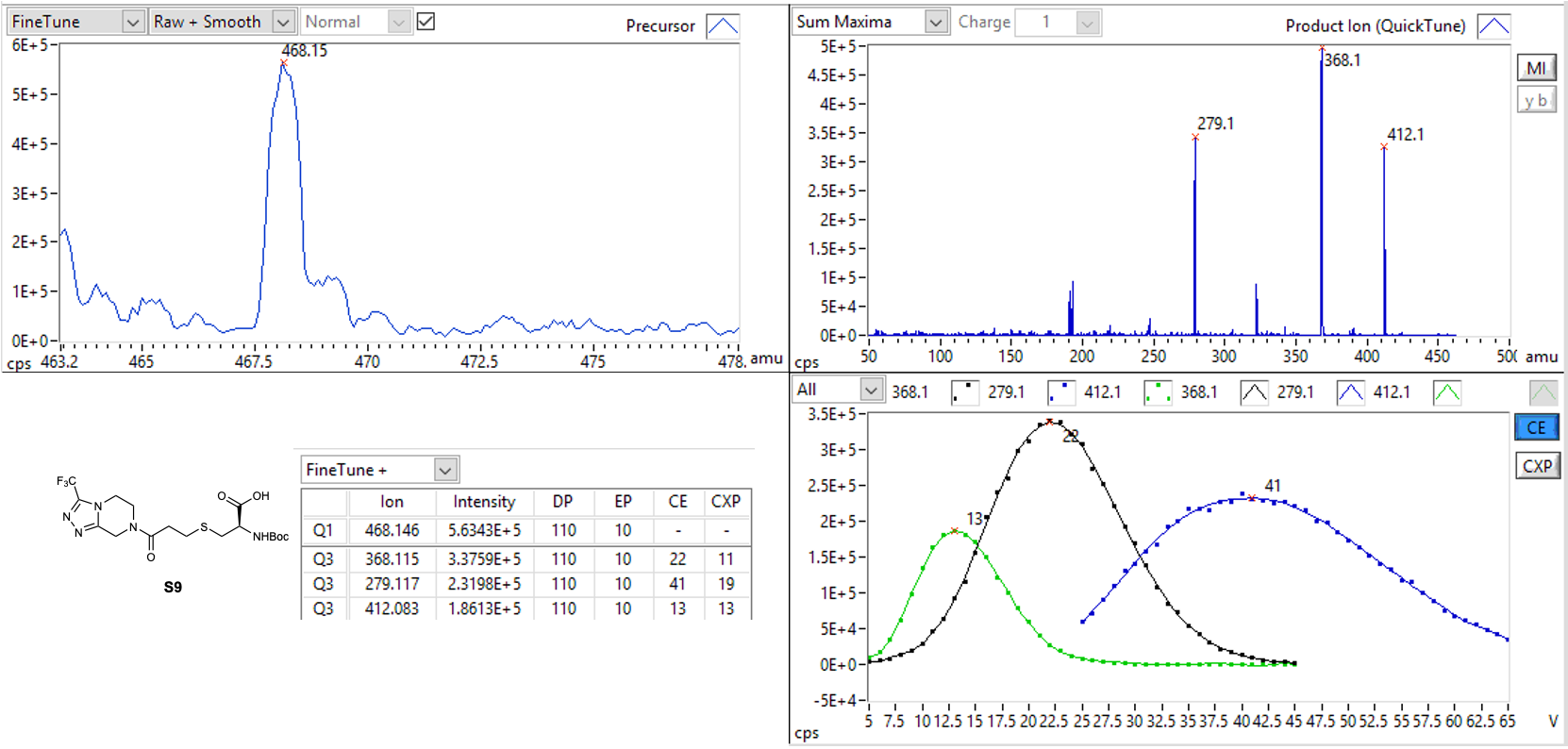
**

**
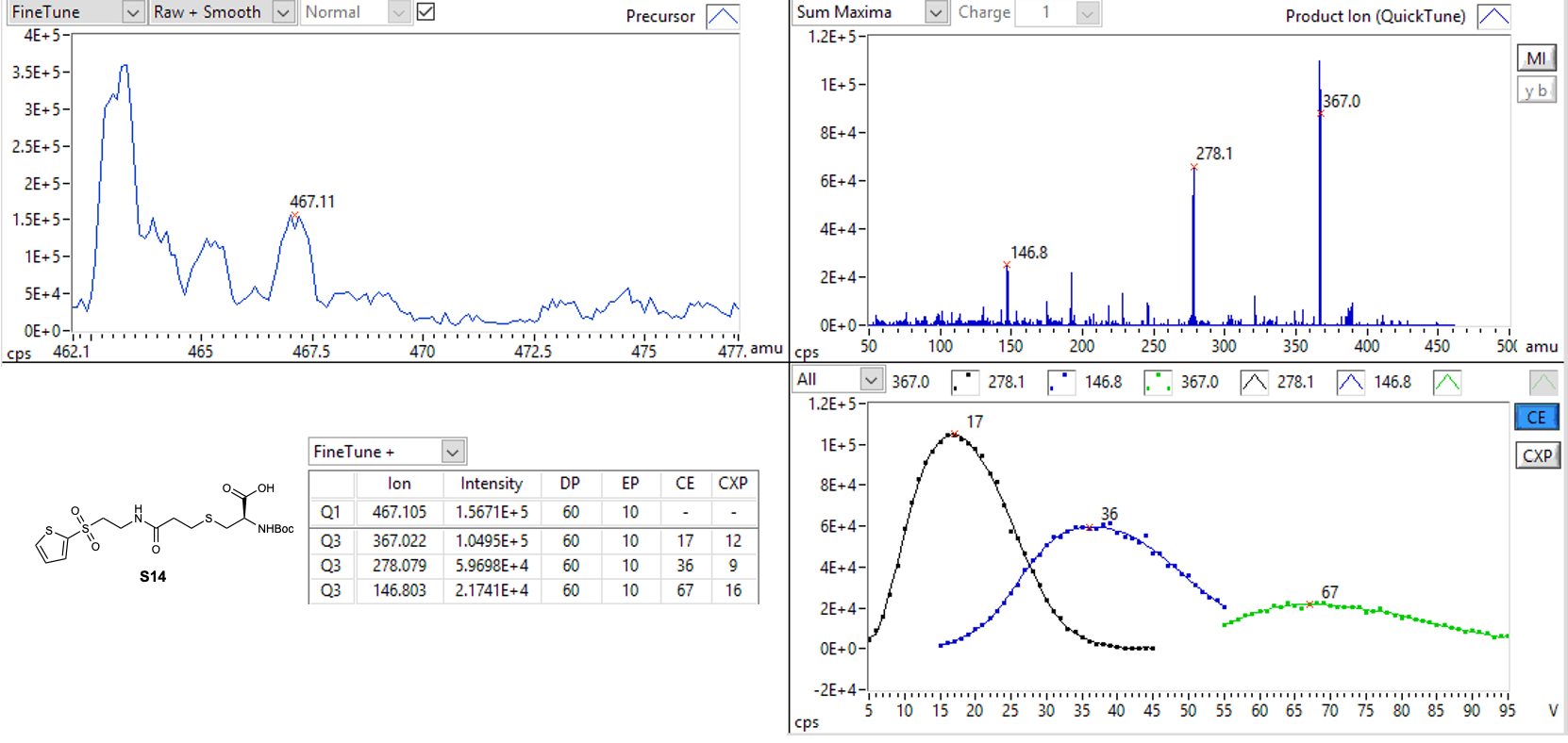
**

**
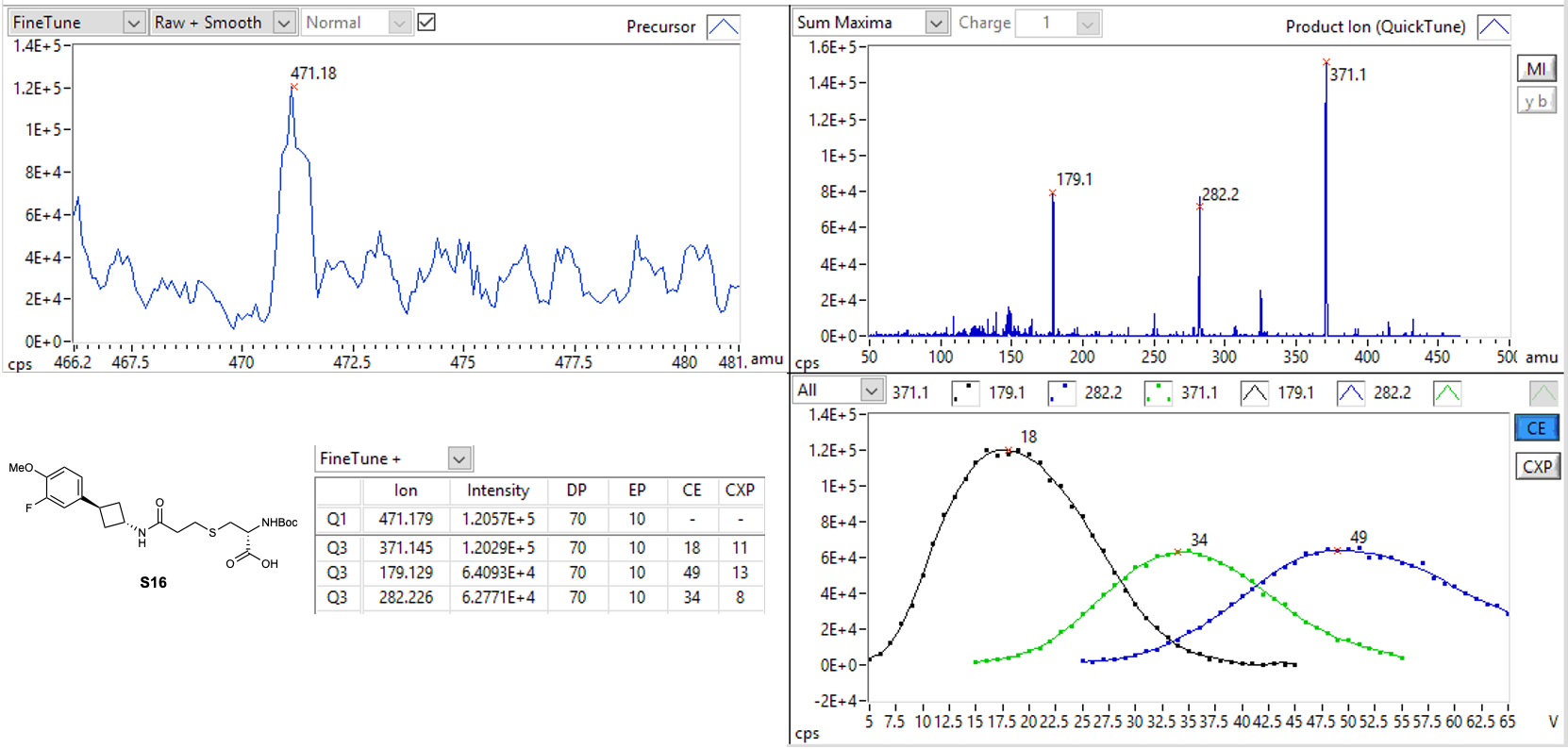
**

**
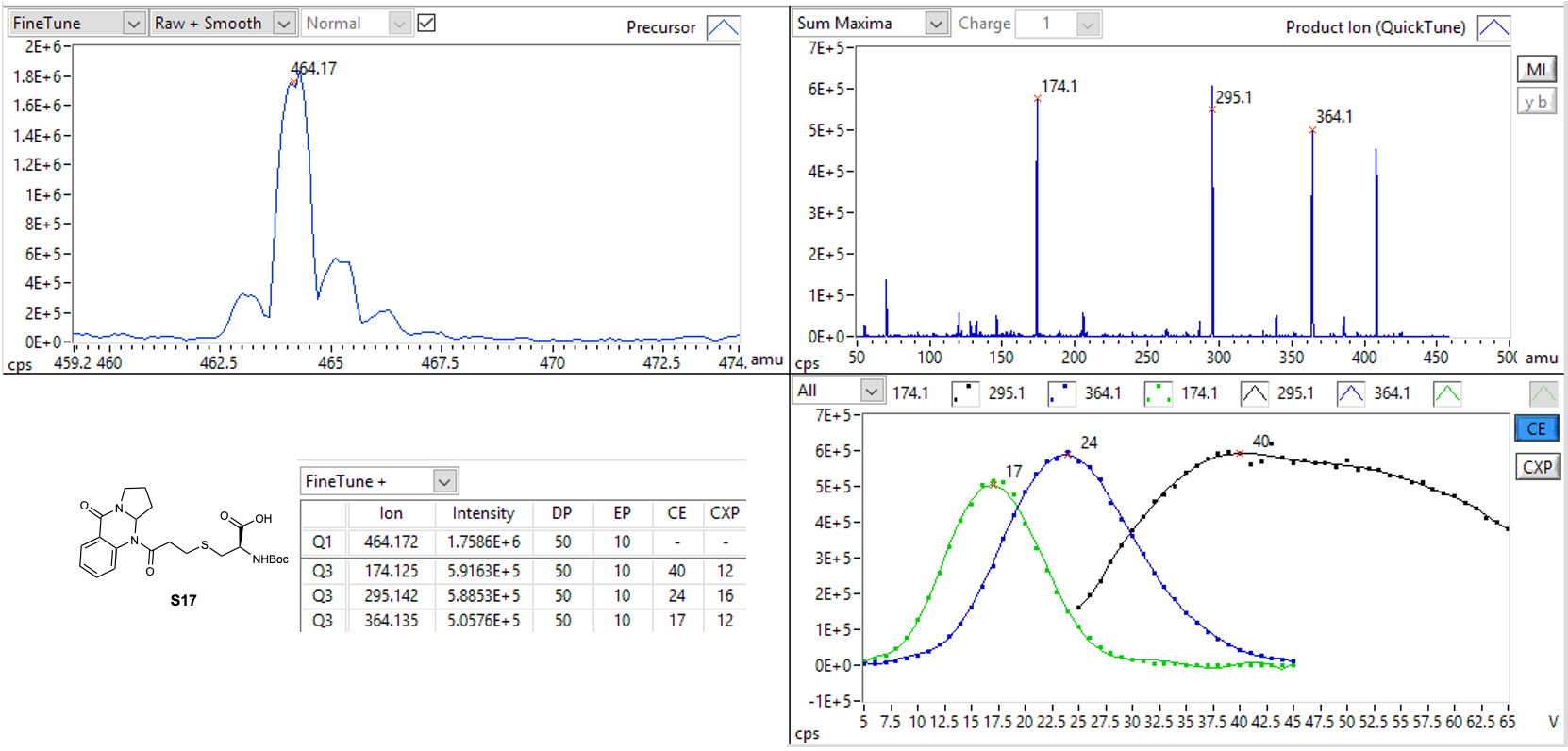
**

**
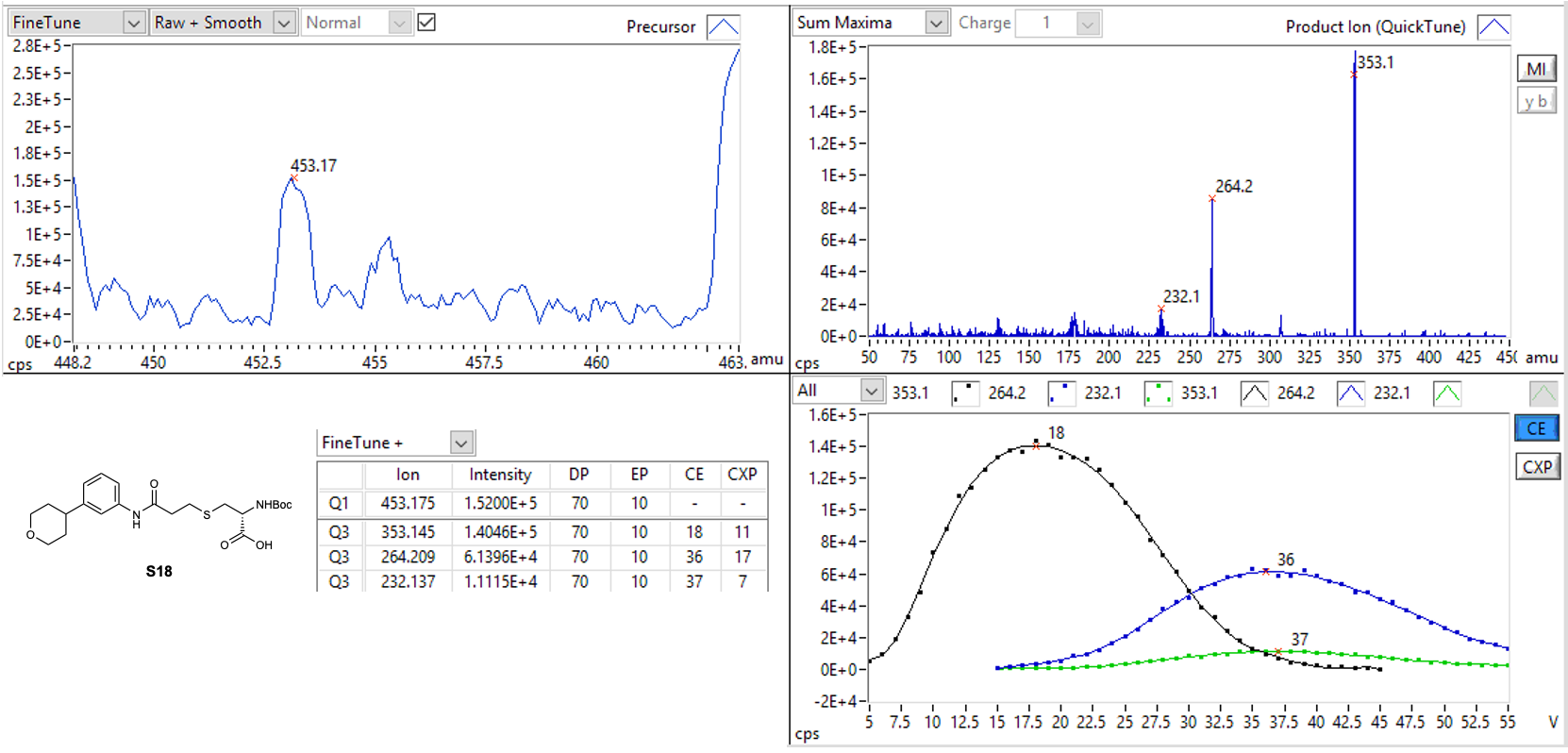
**

**
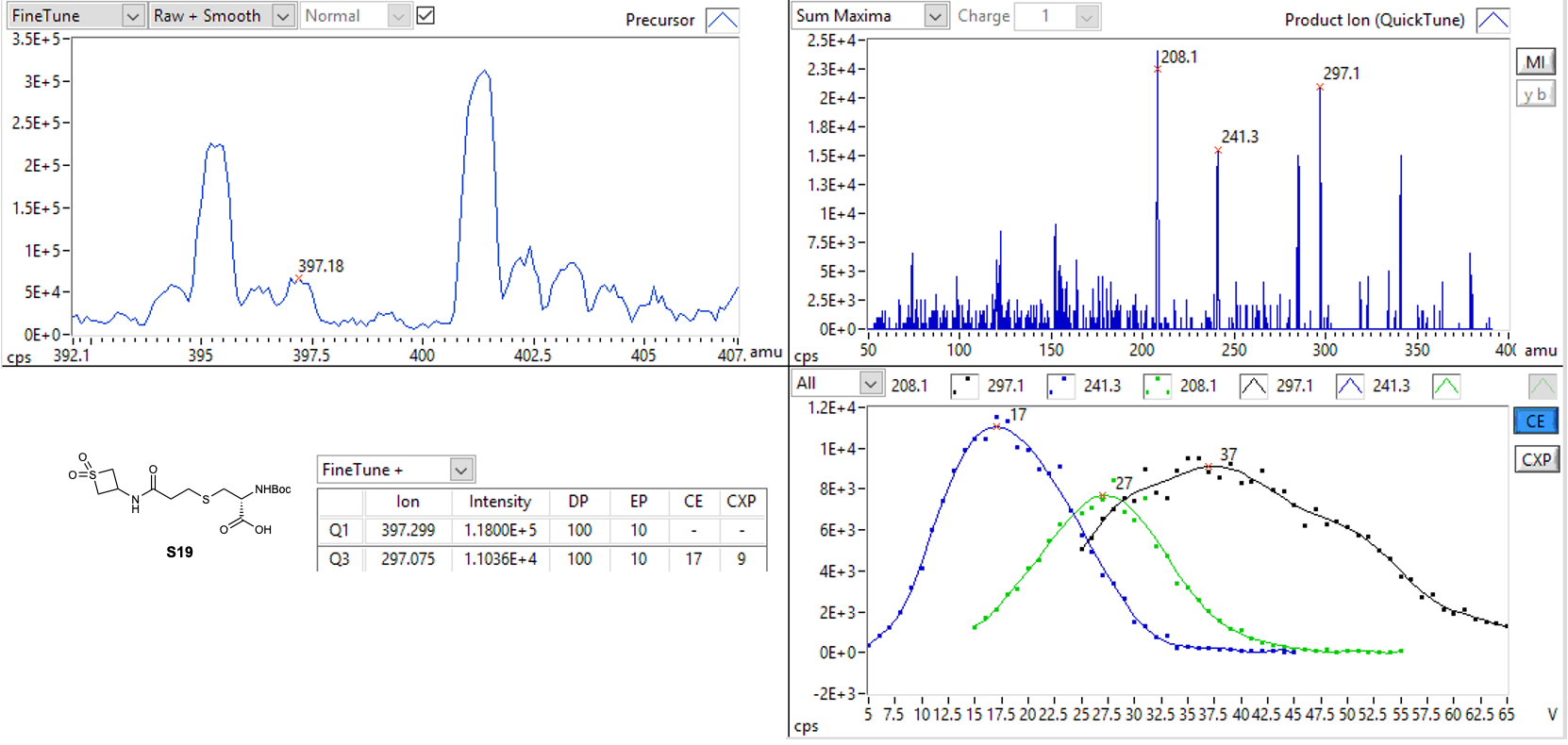
**

**
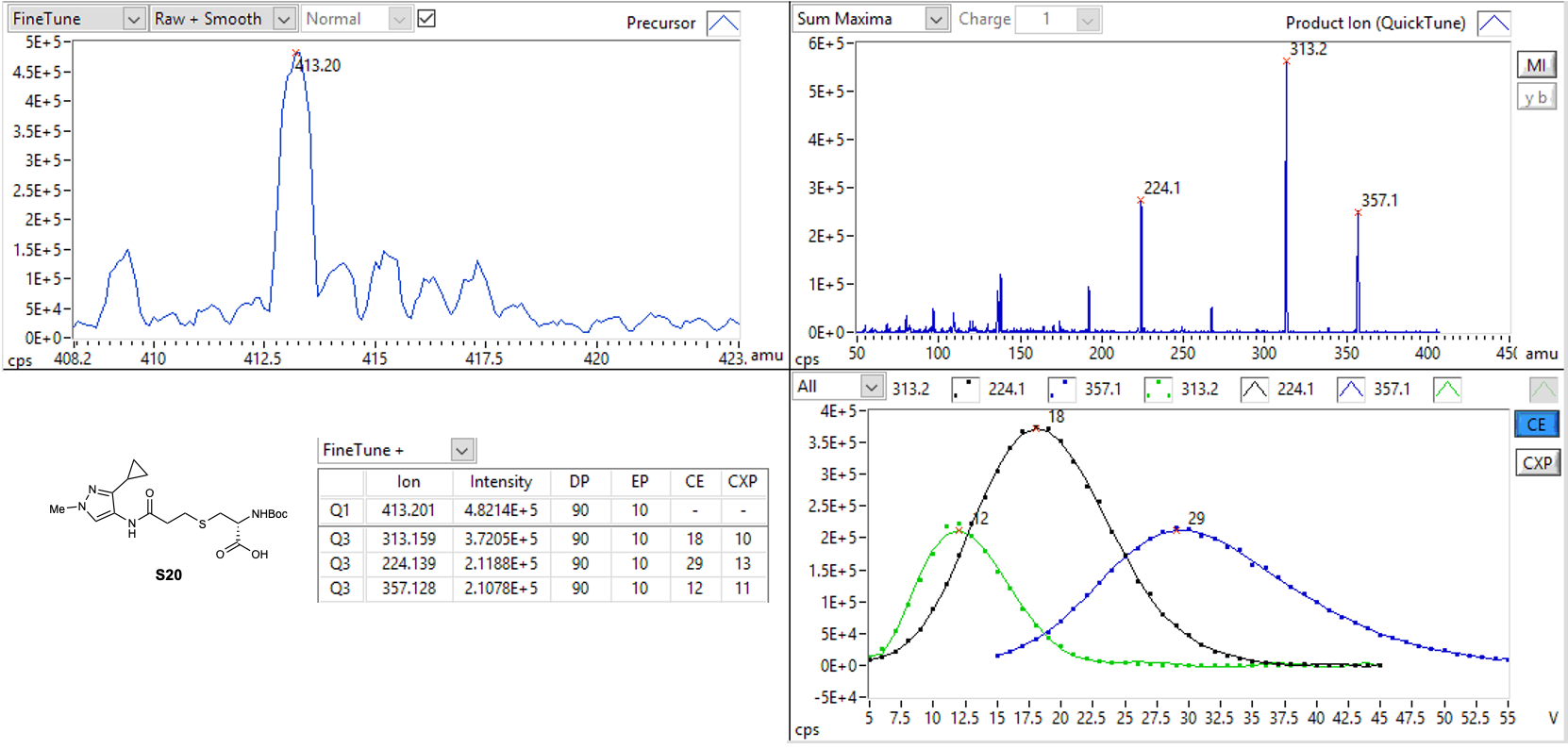
**

**
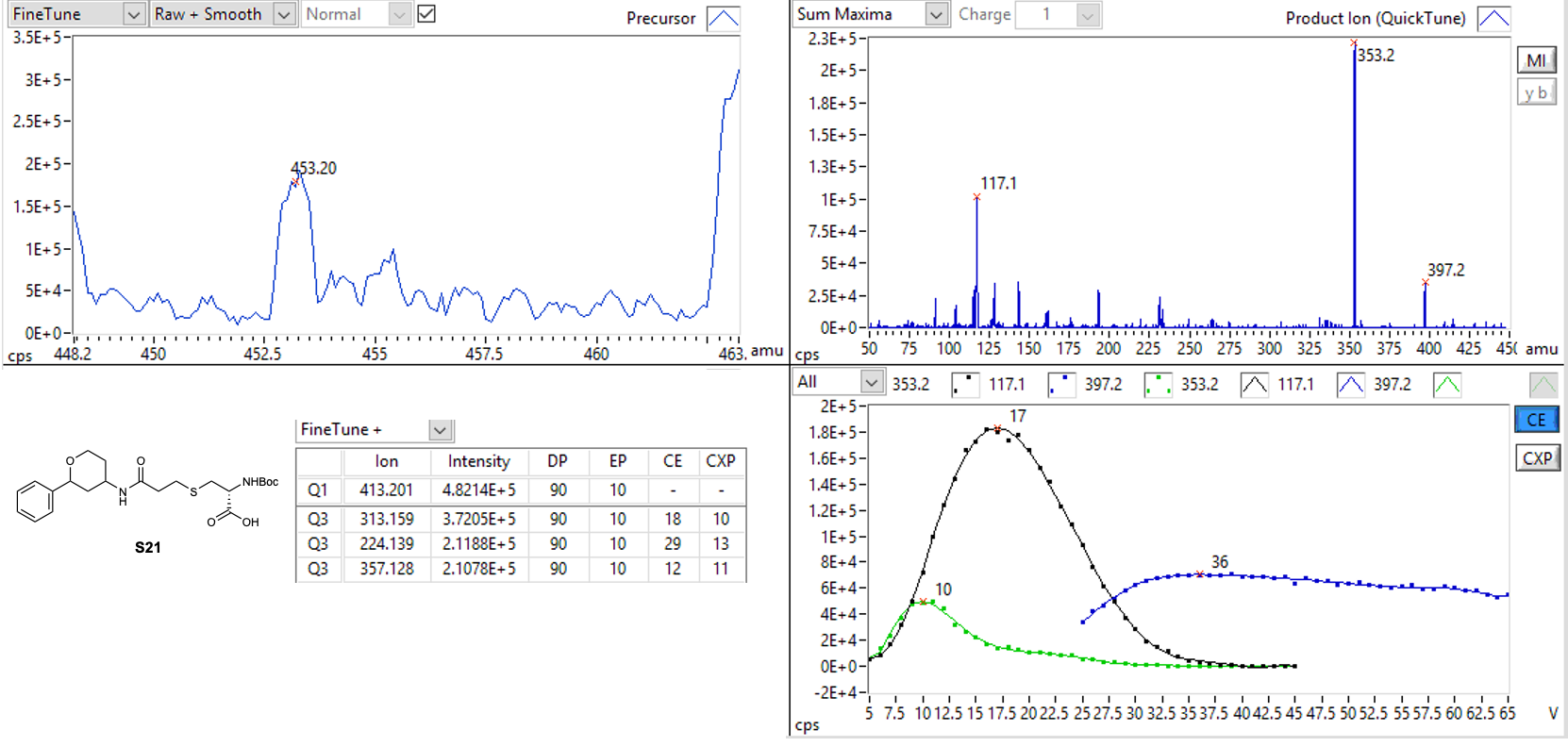
**

**
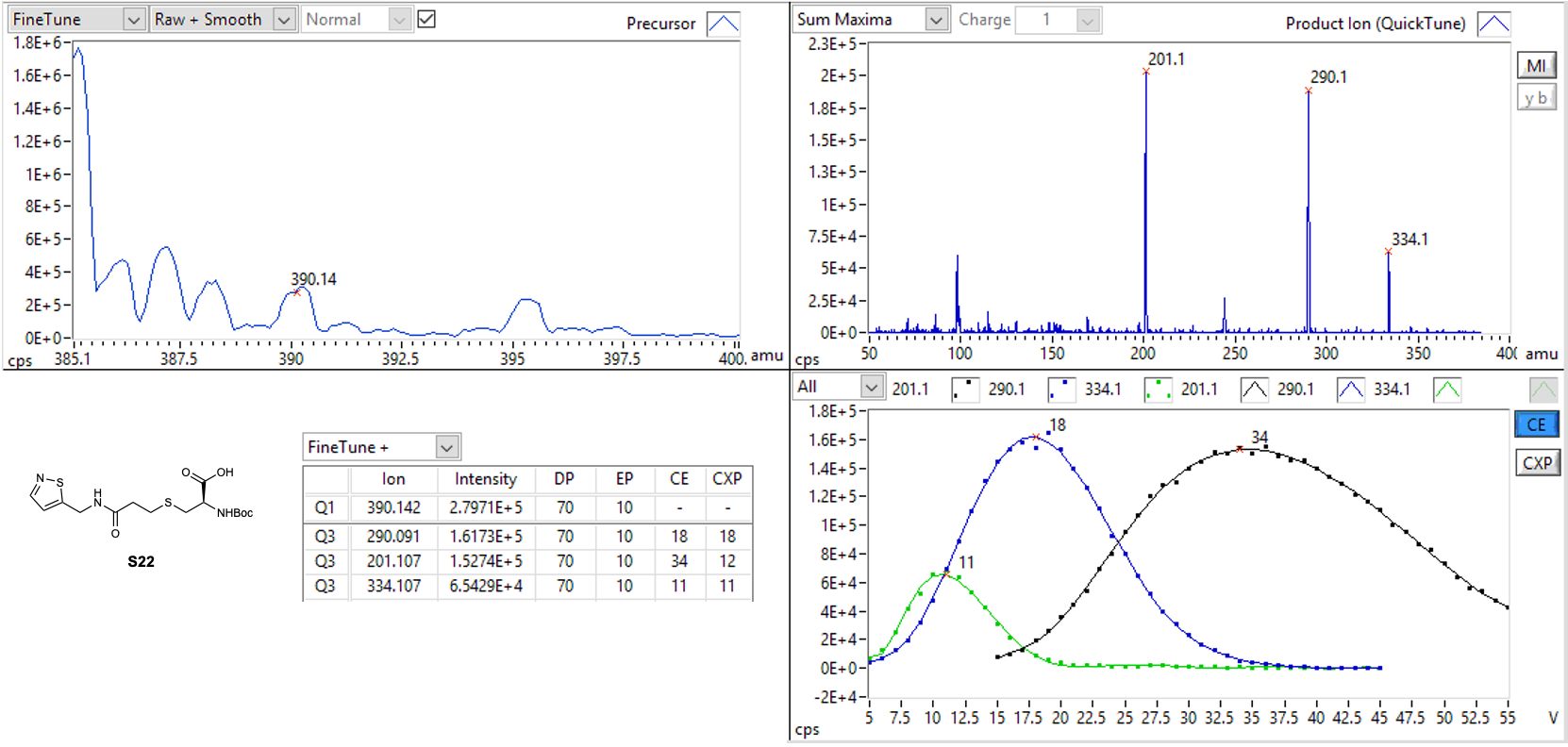
**

**
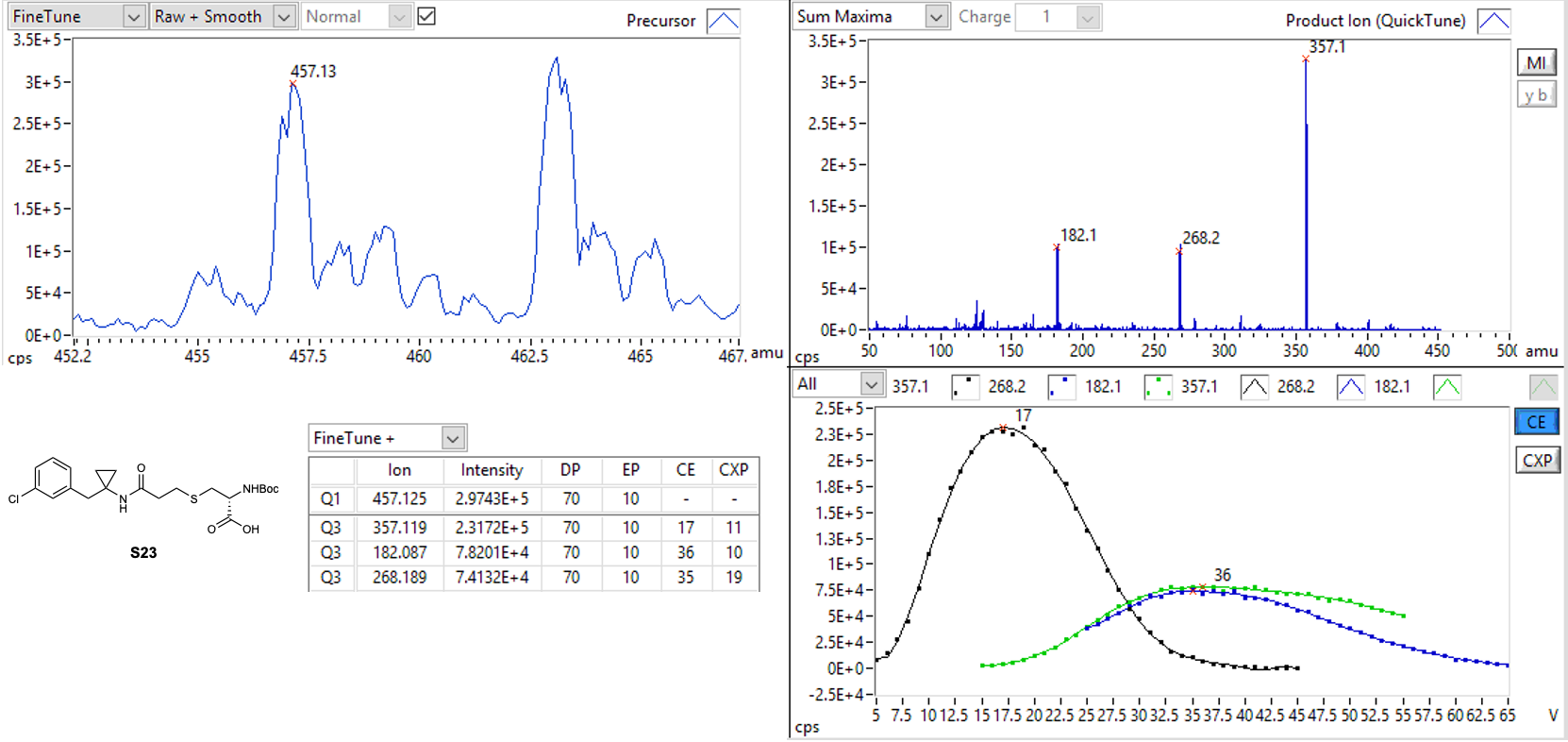
**

**
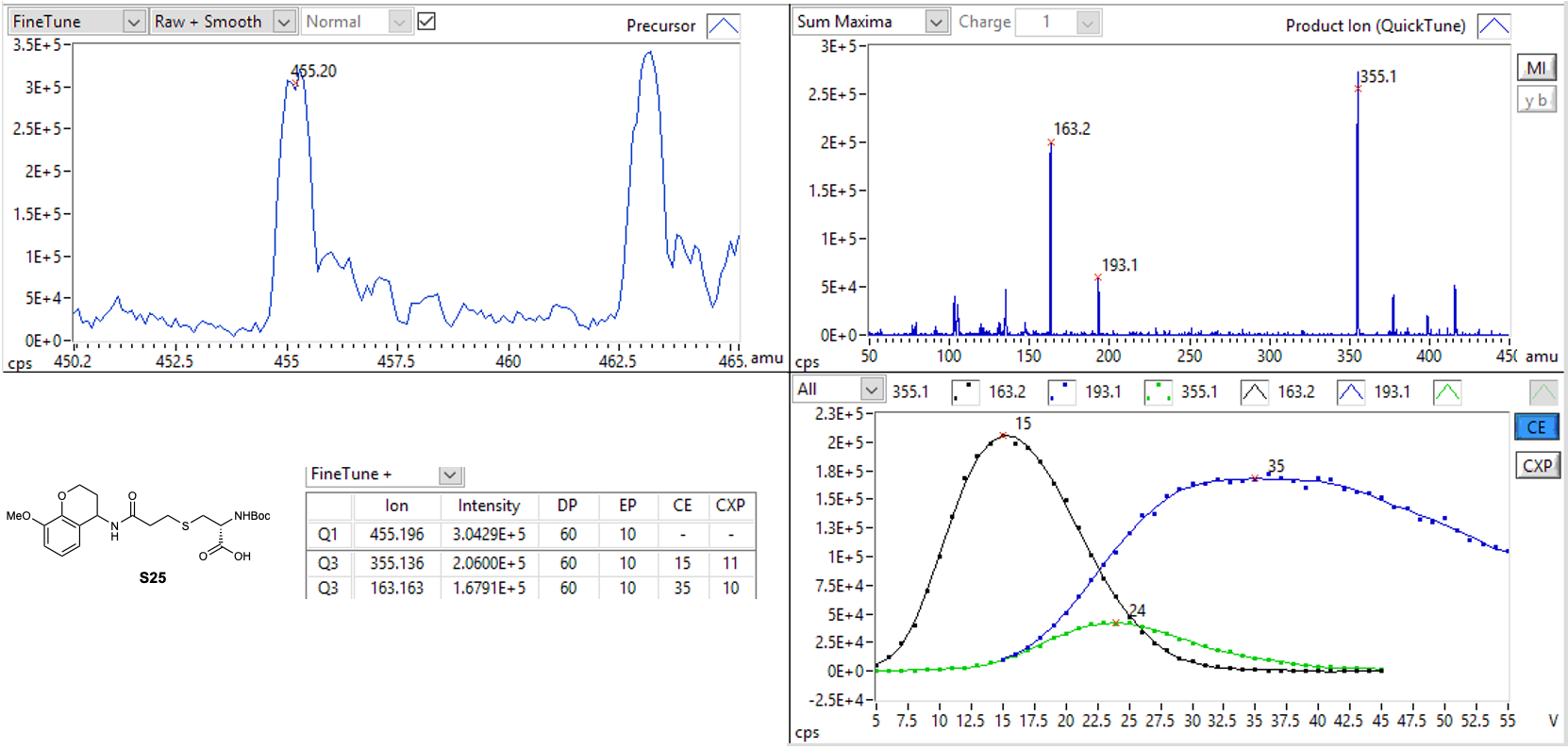
**

**
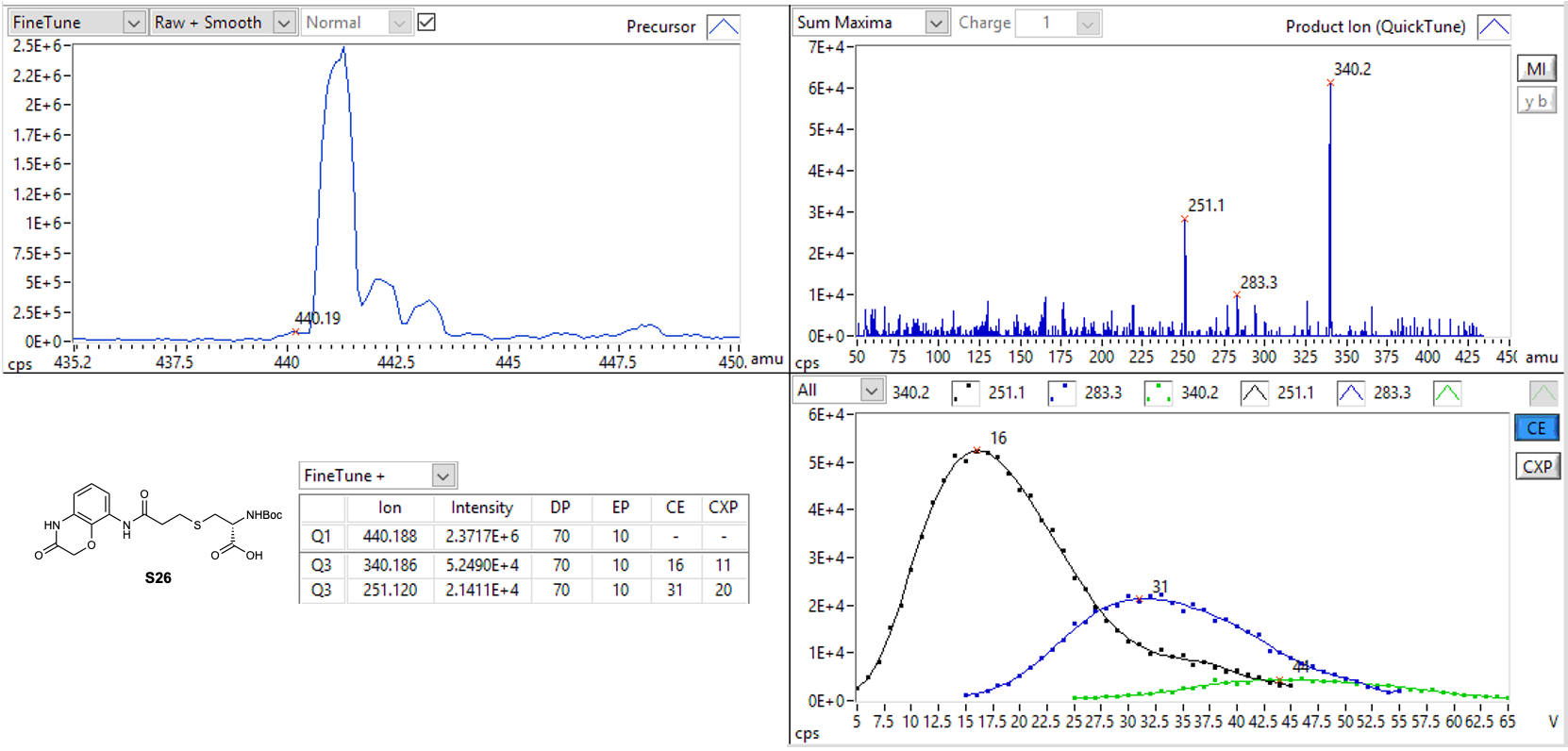
**

**
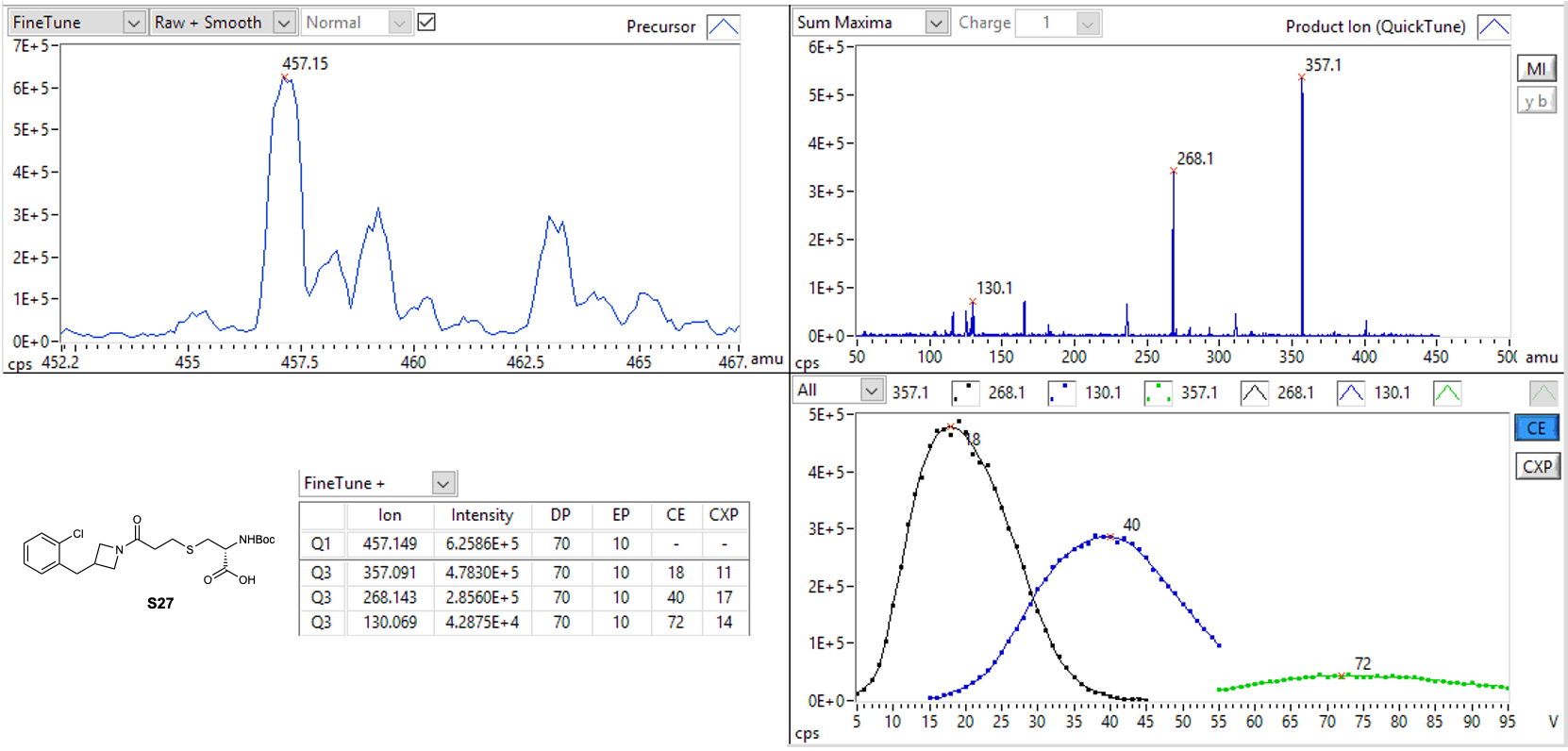
**

**
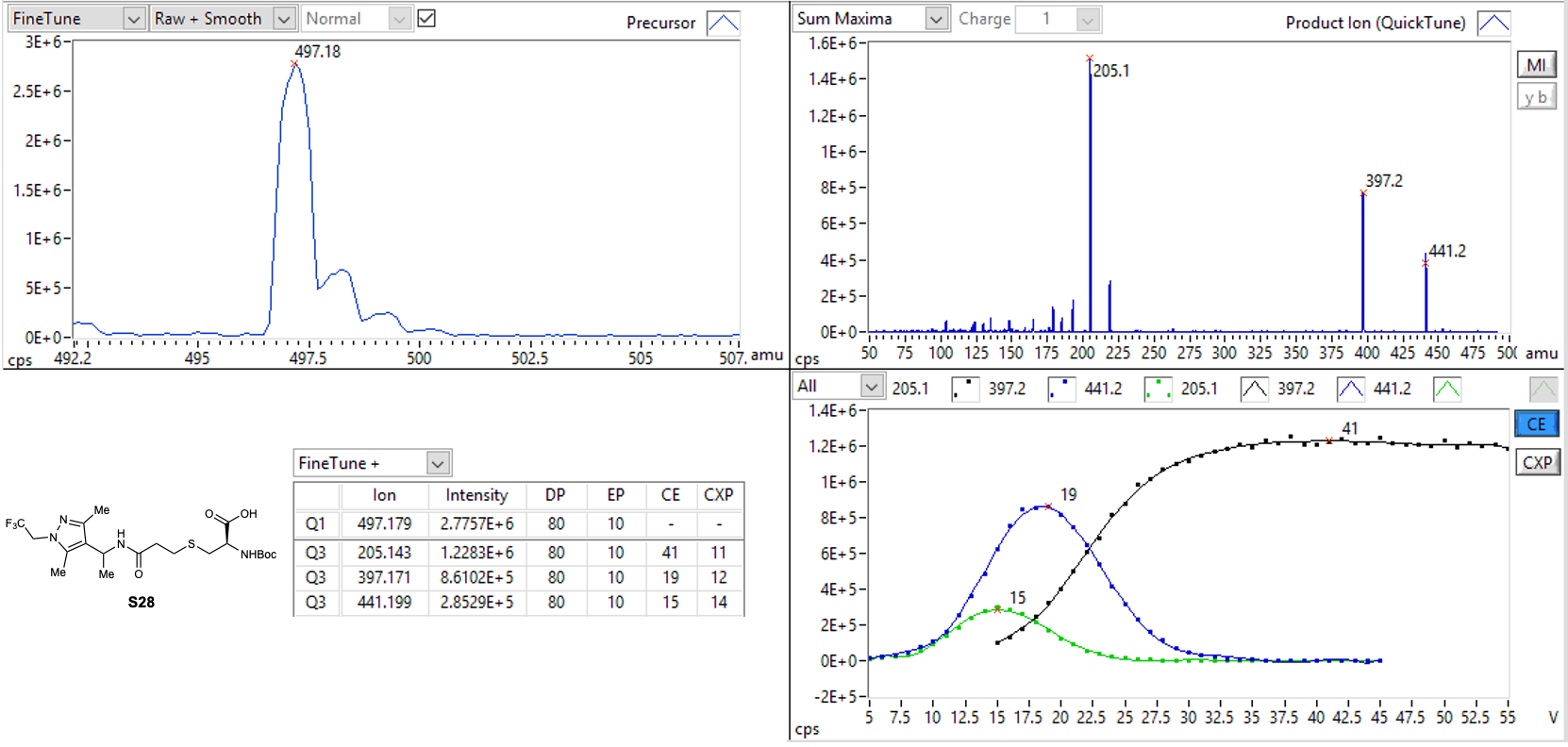
**

**
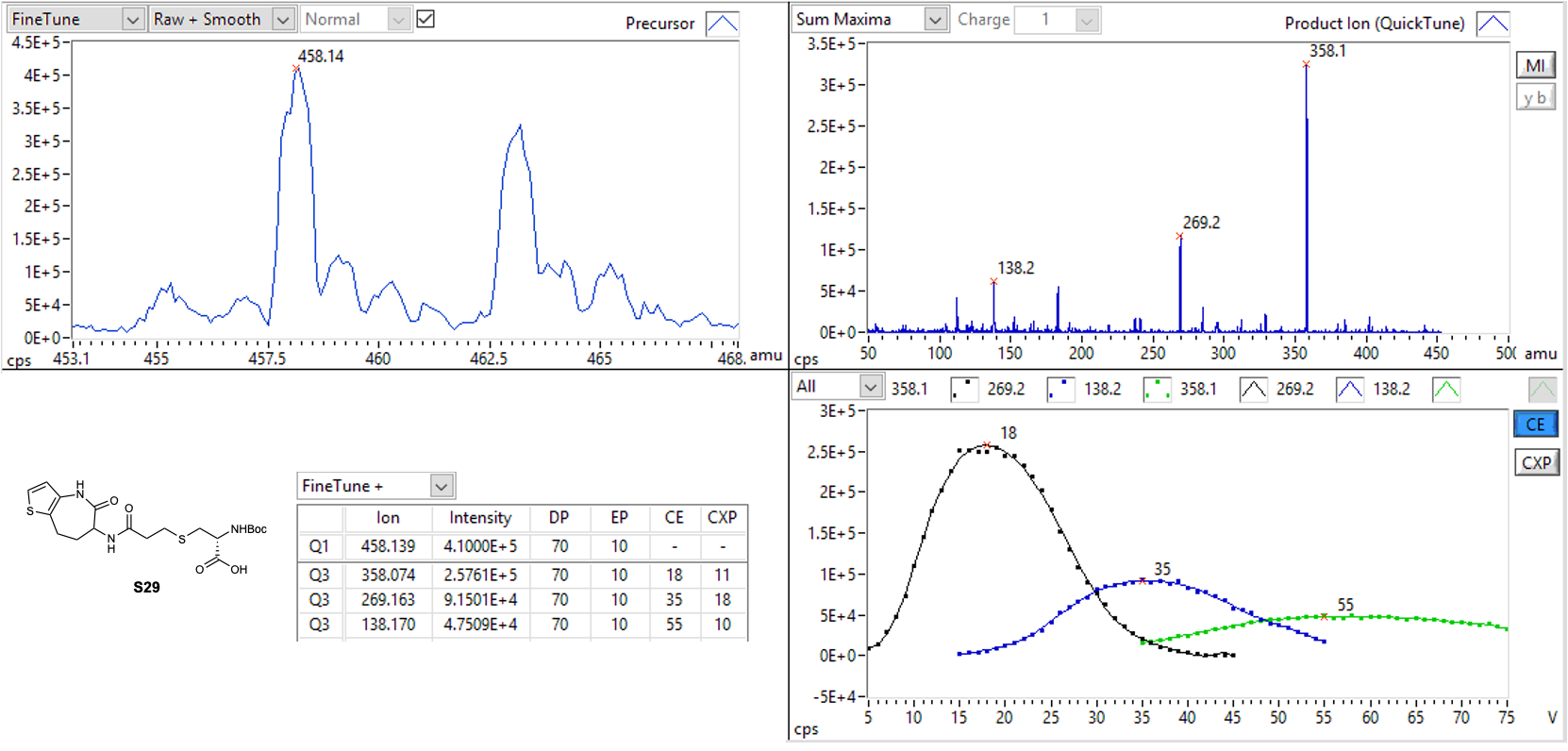
**


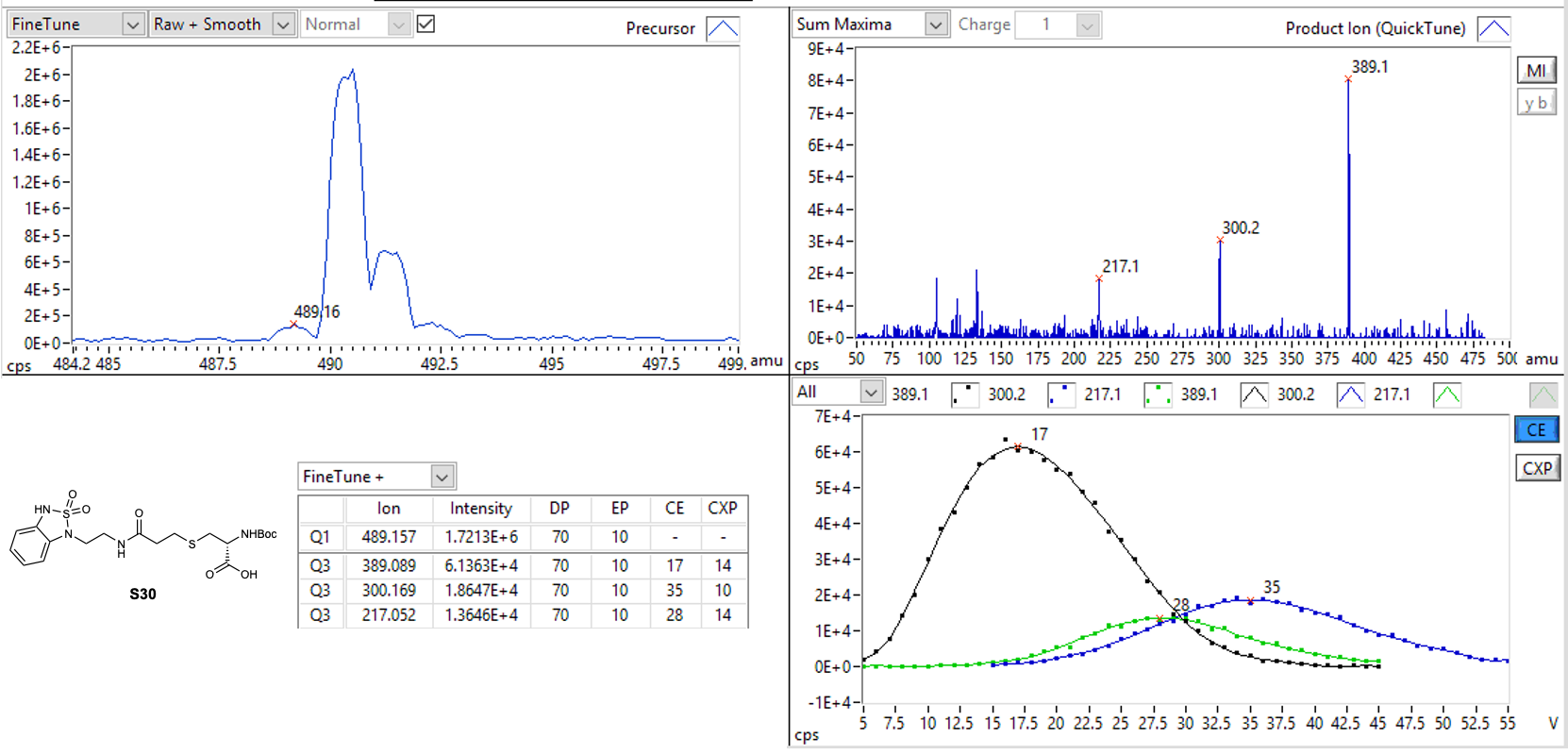


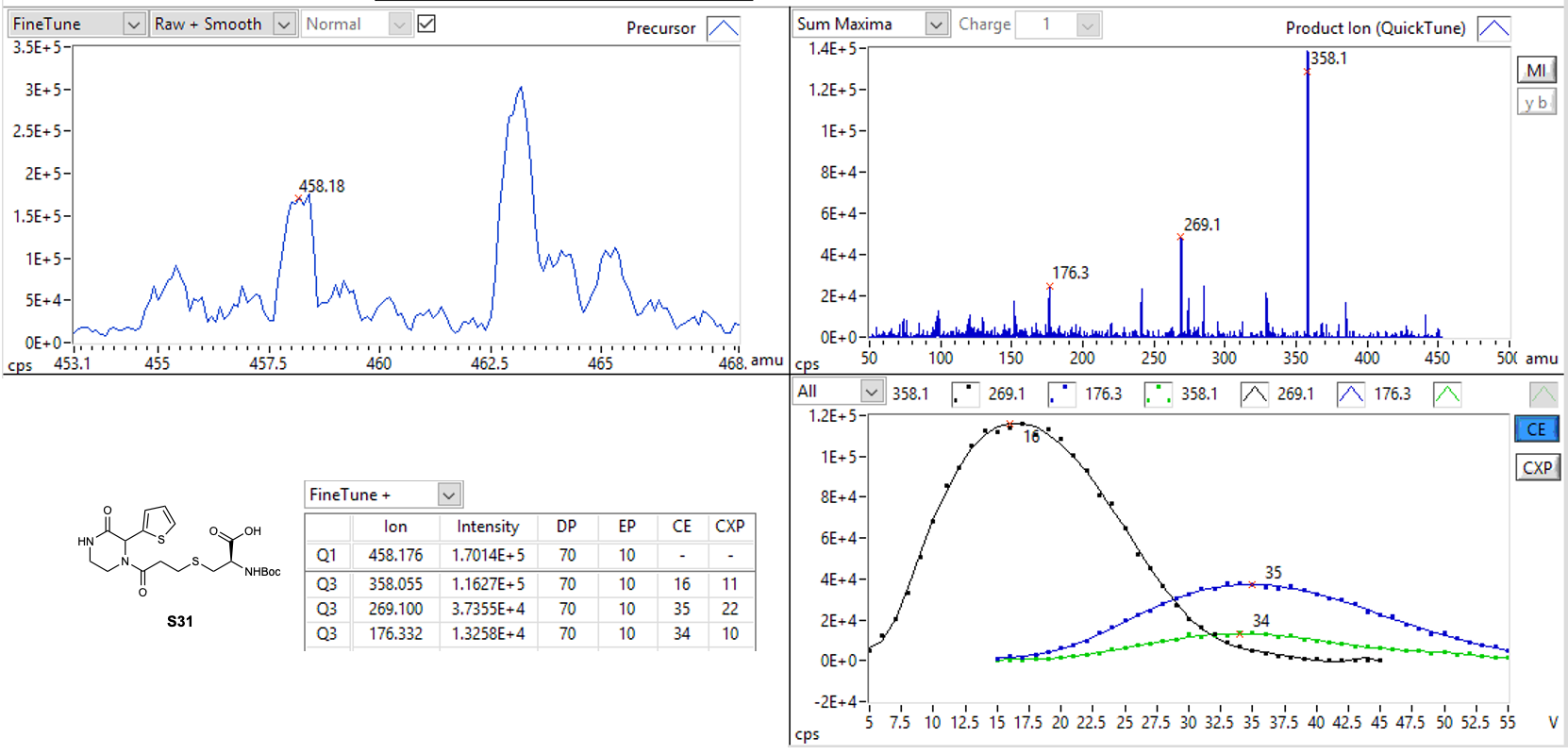


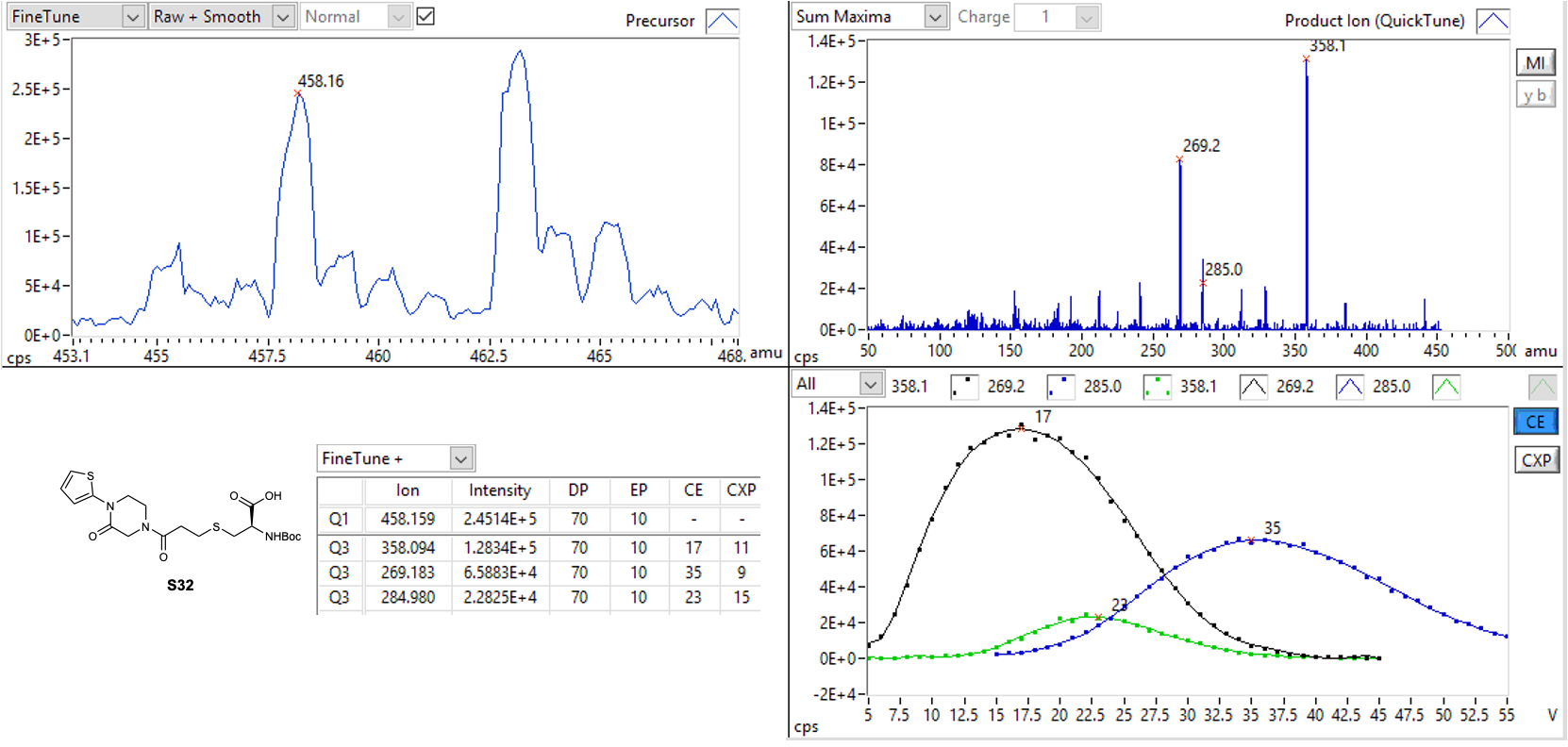


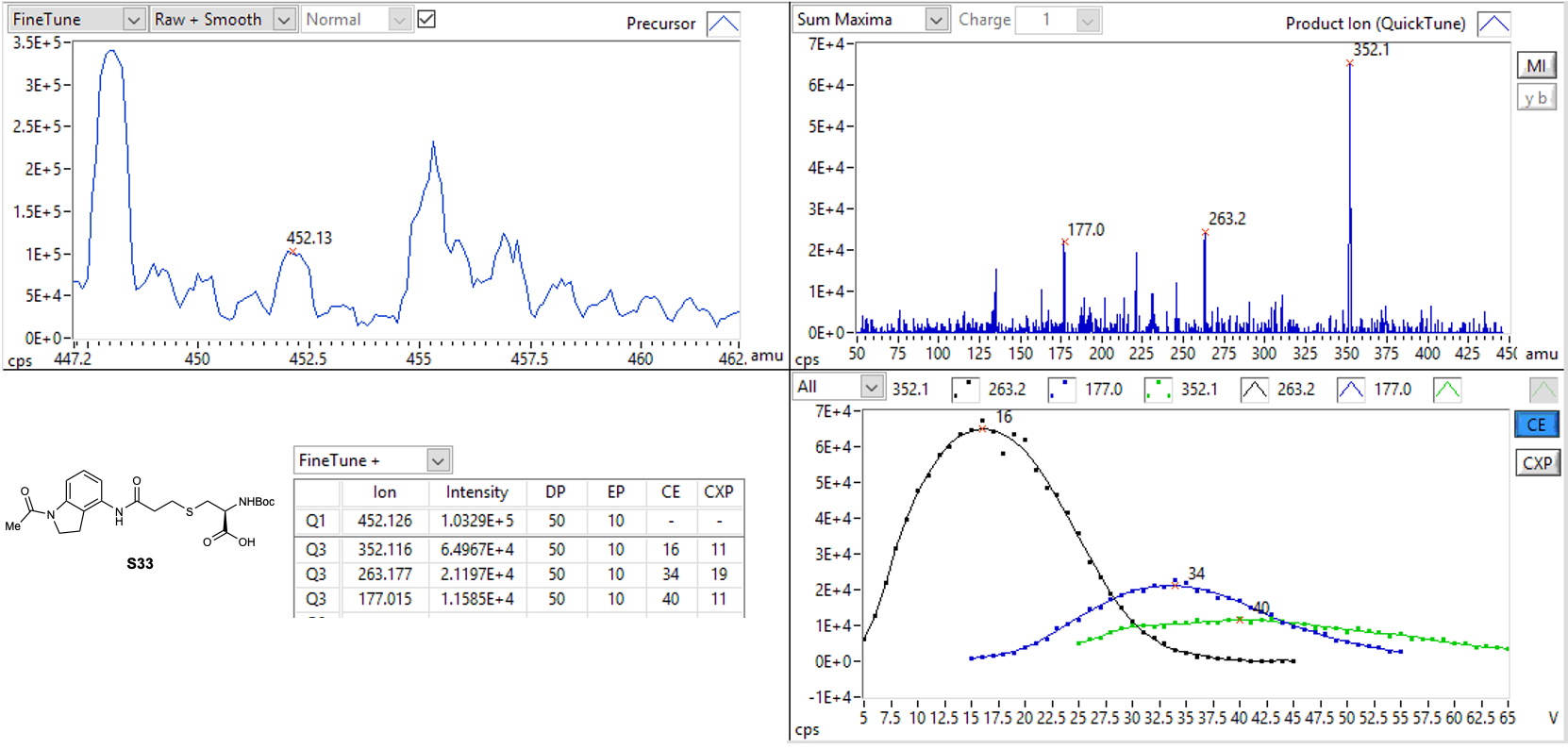


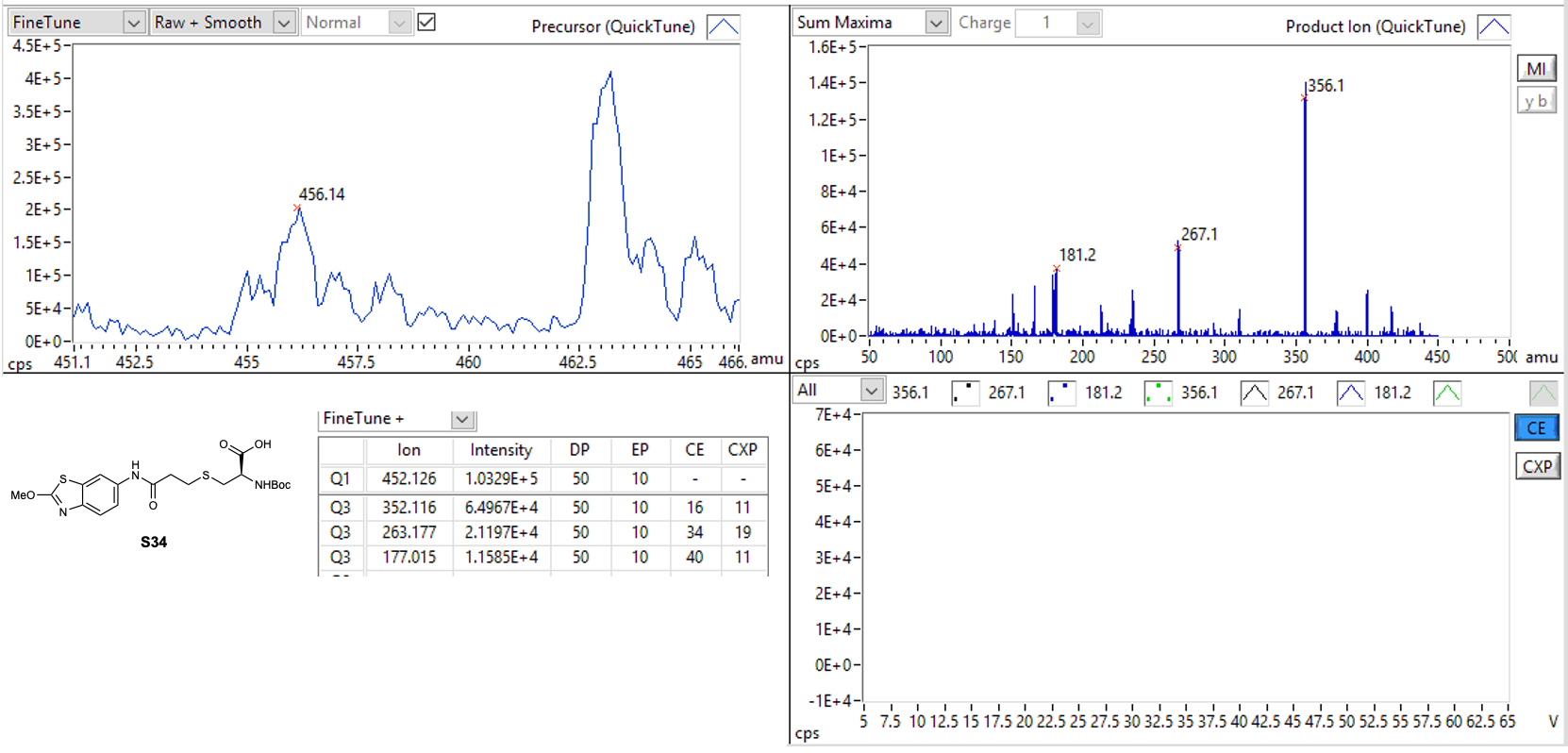


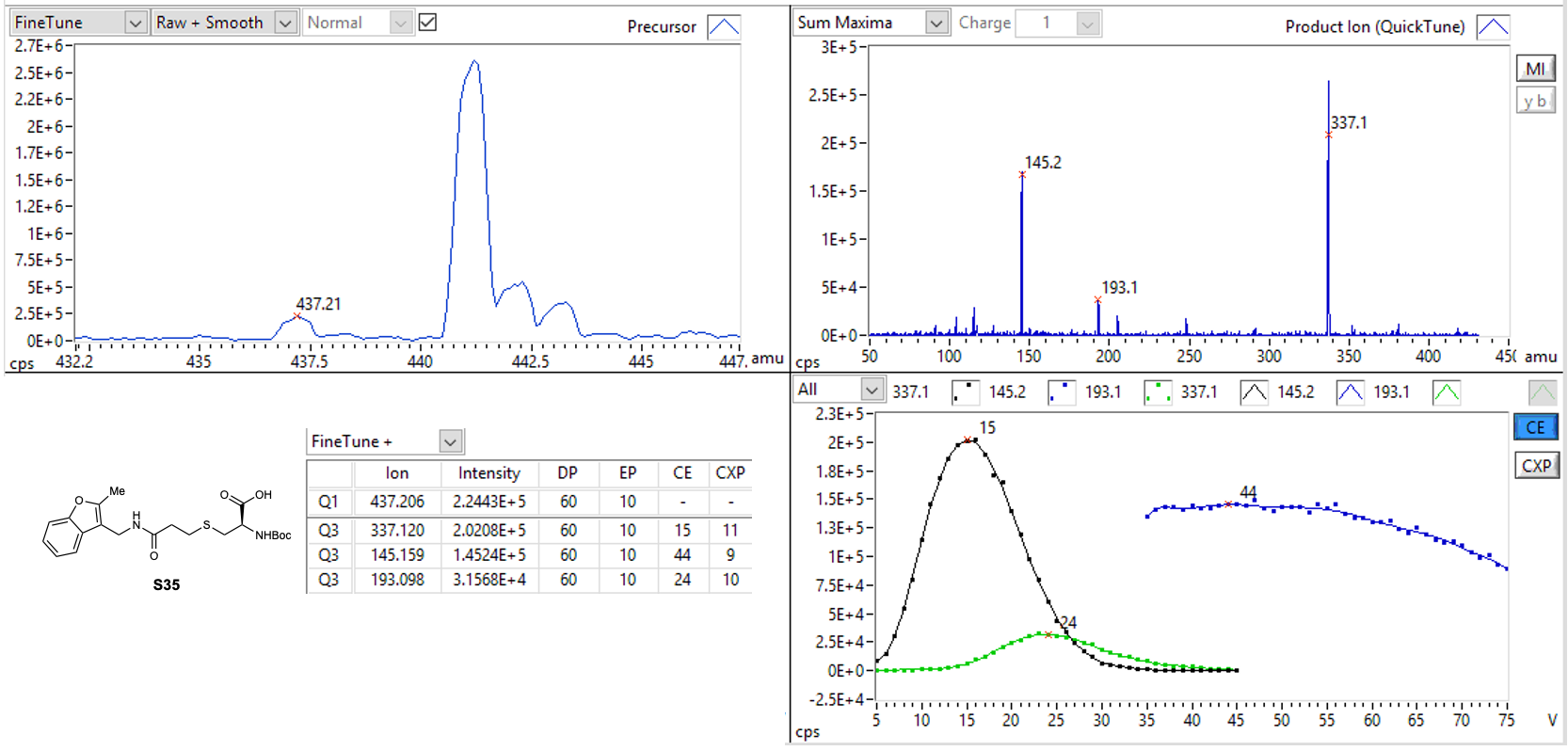


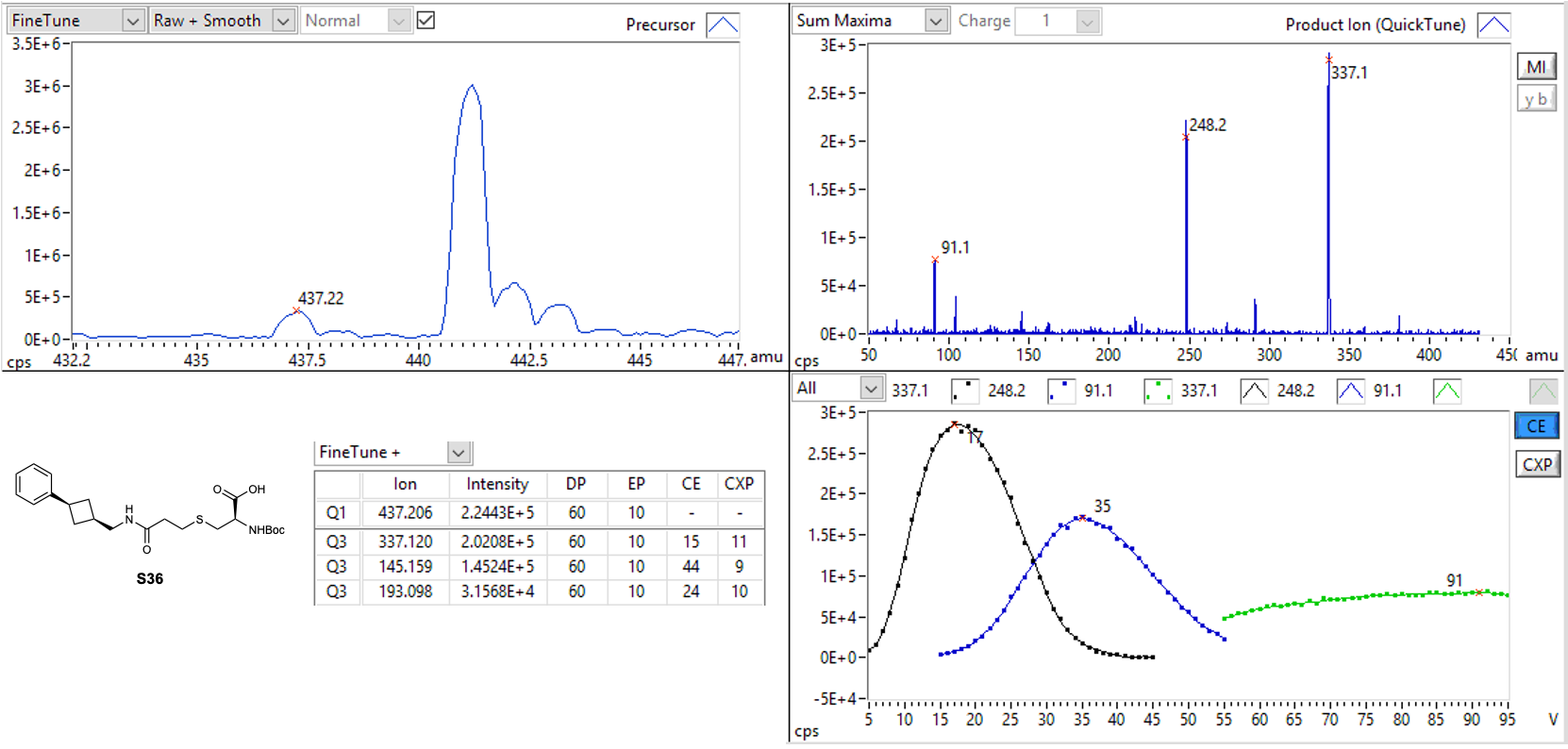


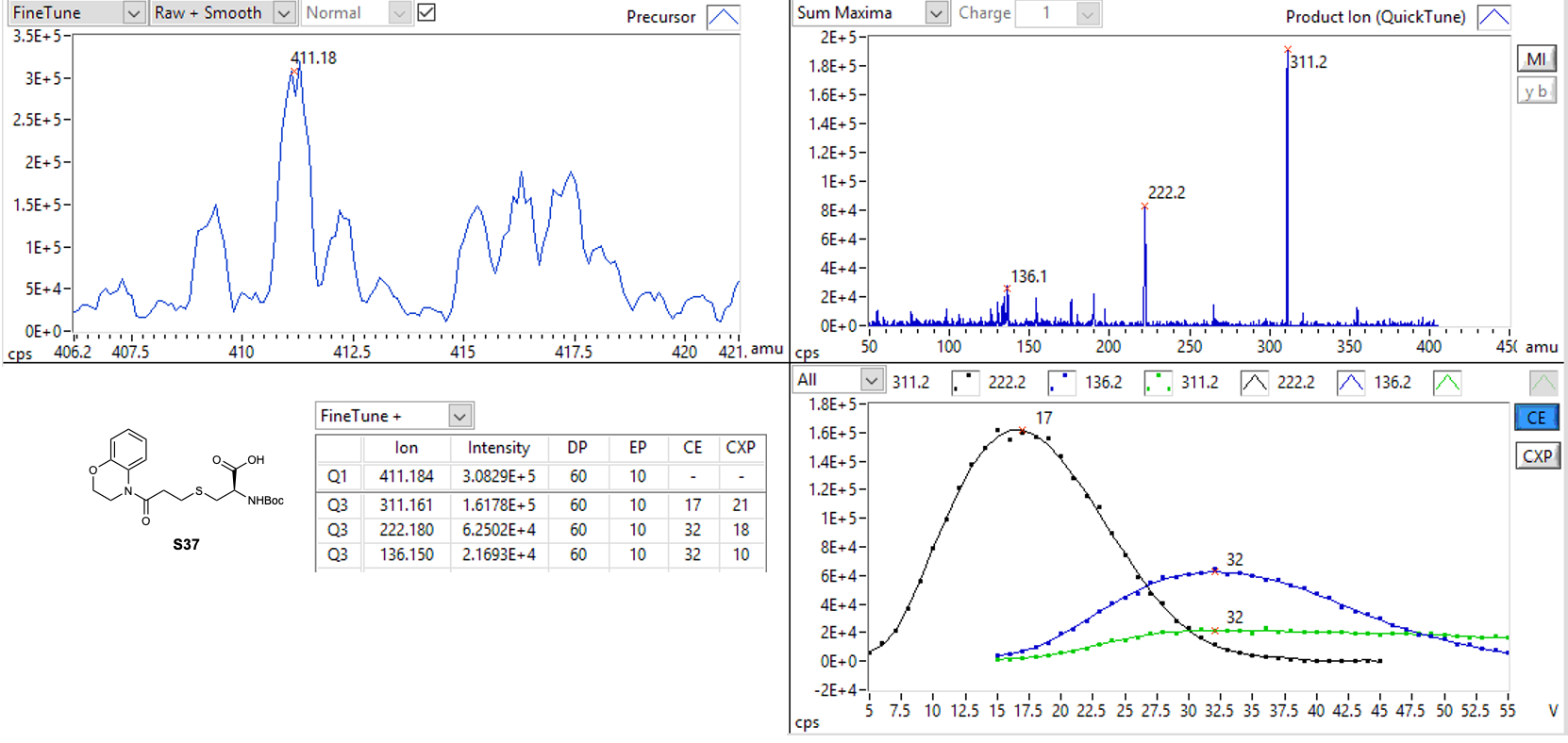


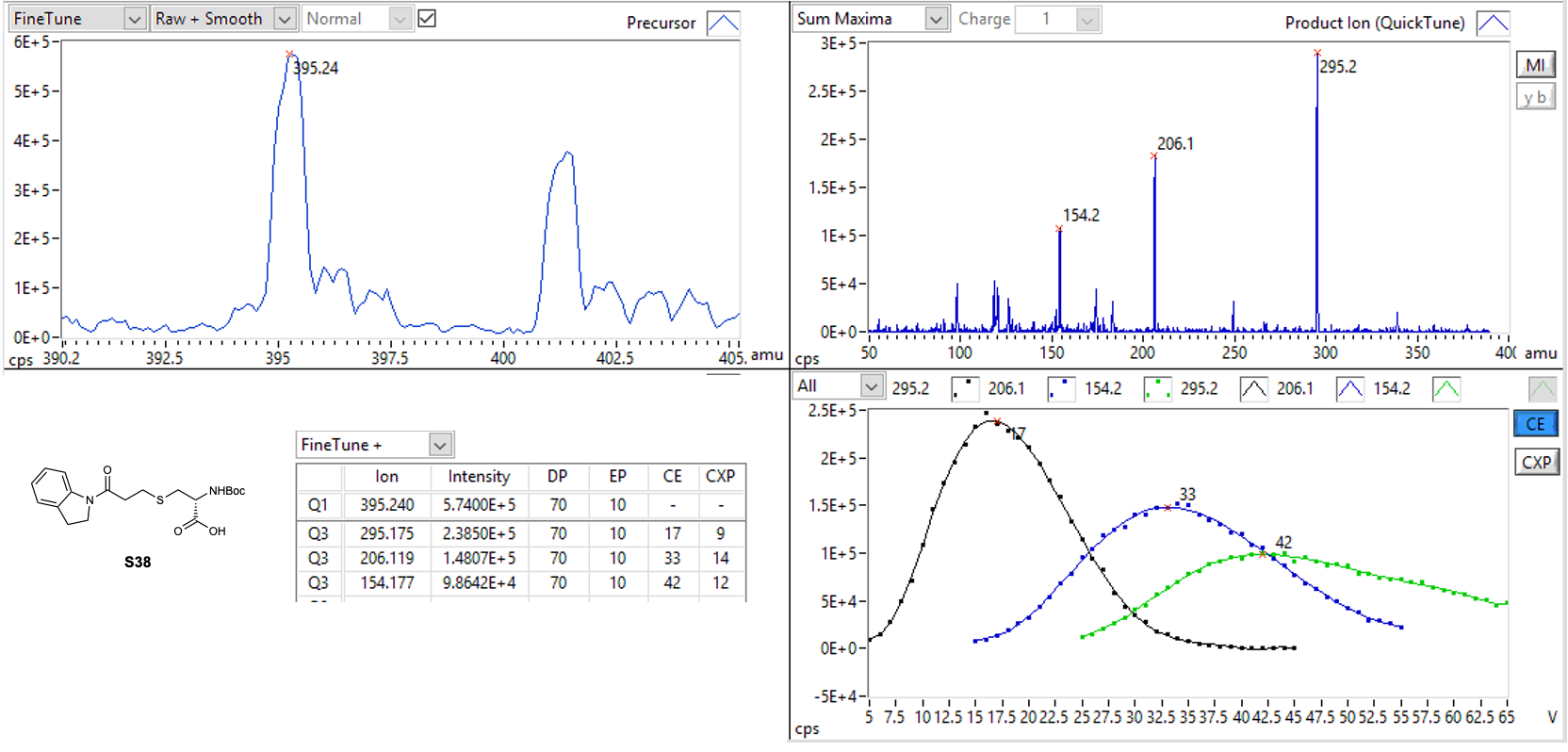


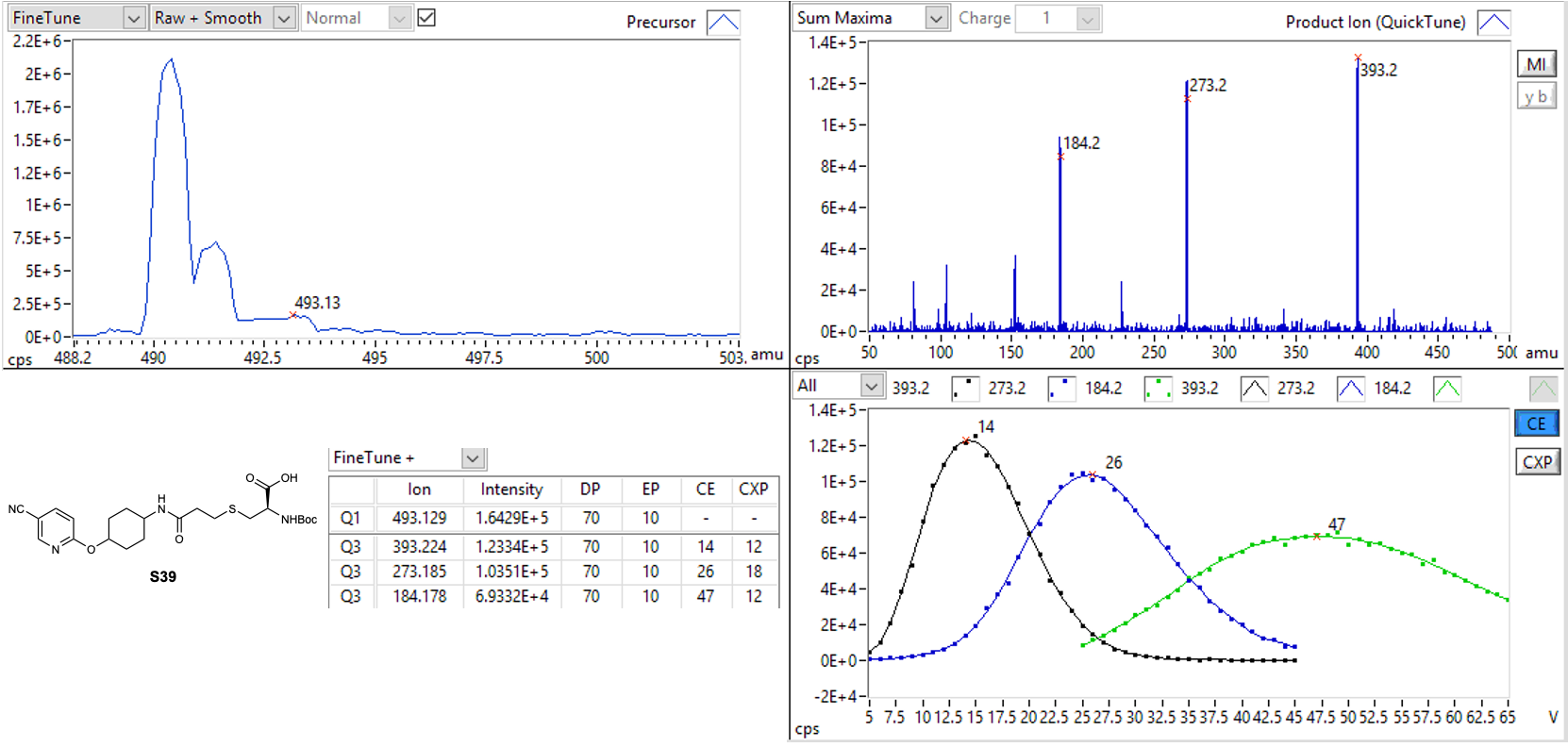


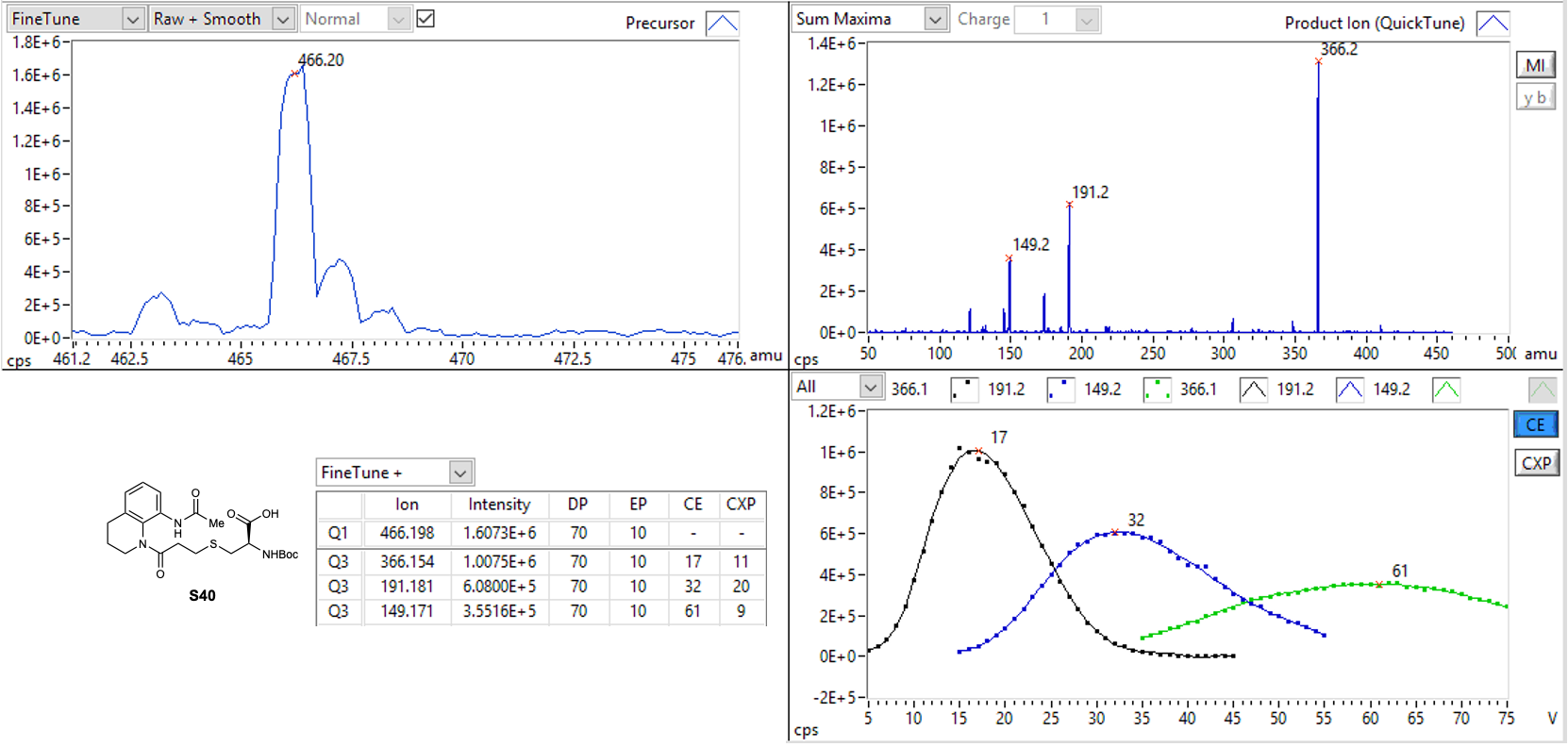


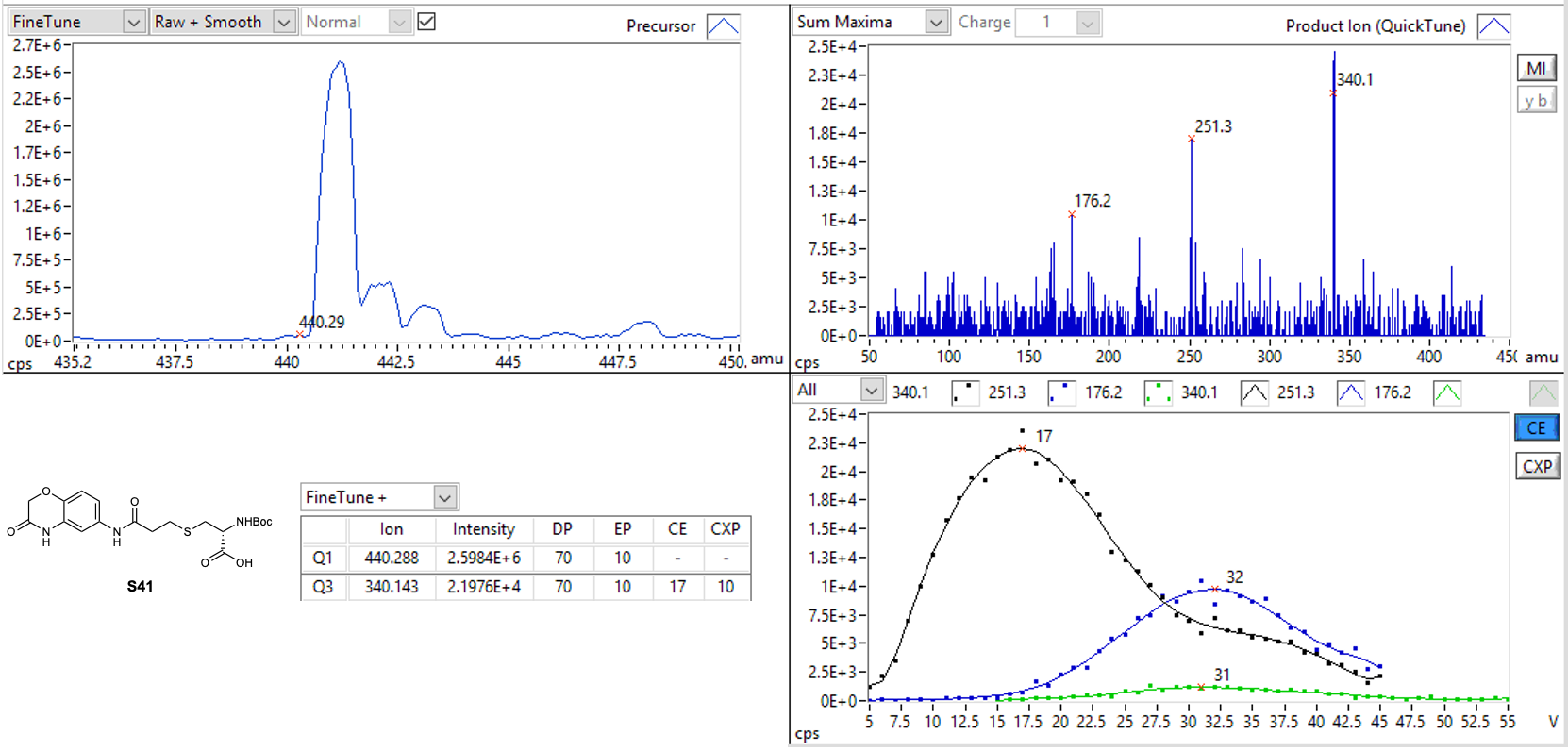


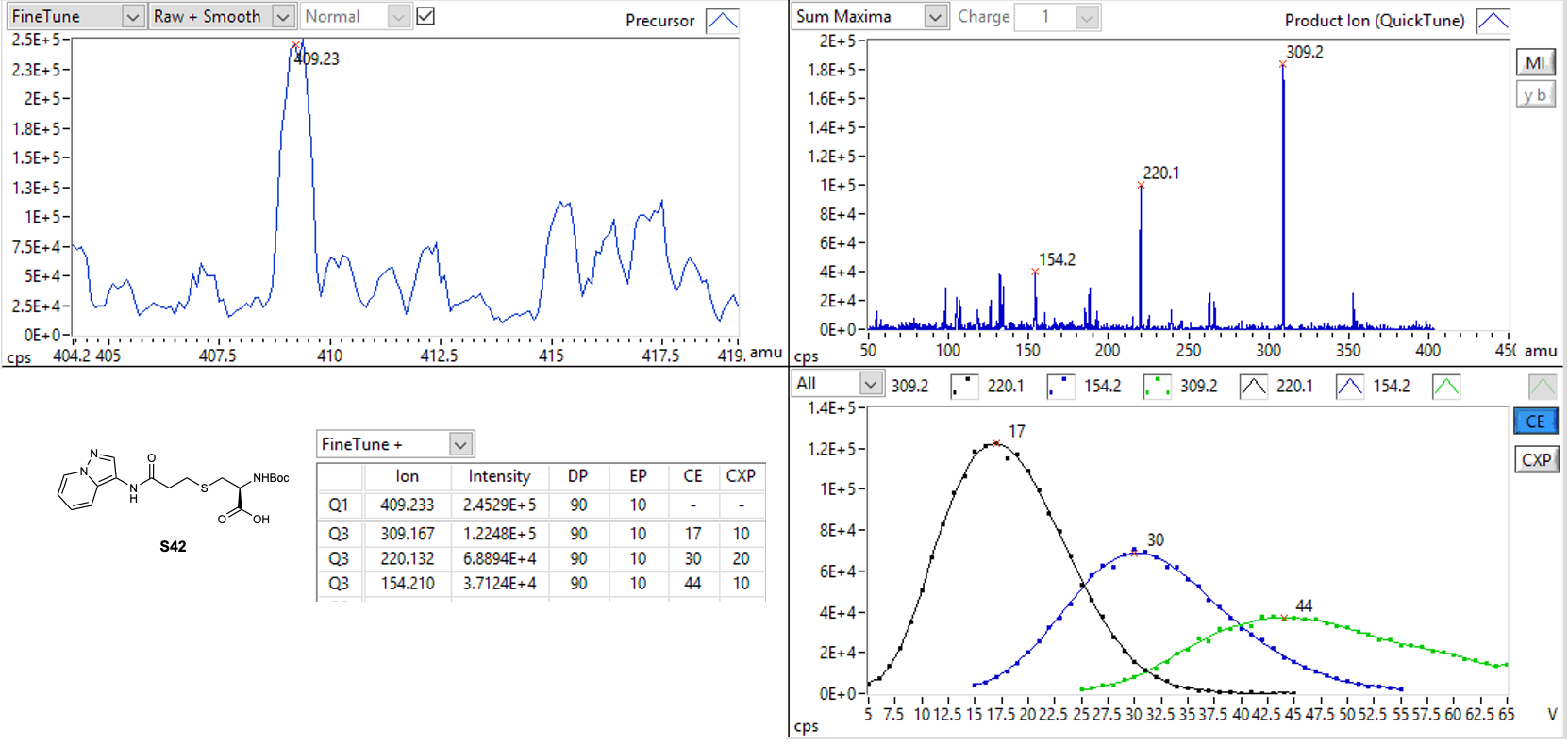


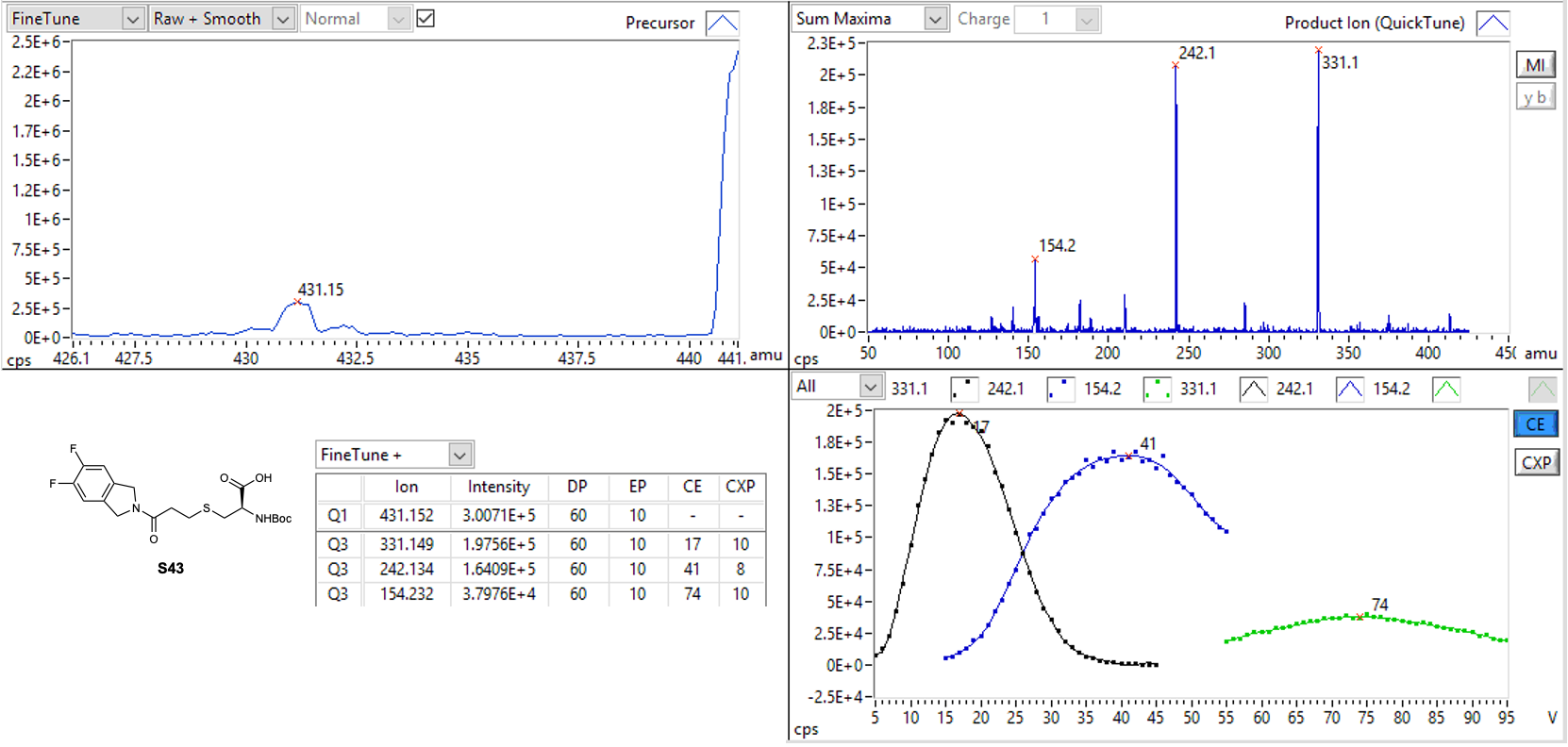


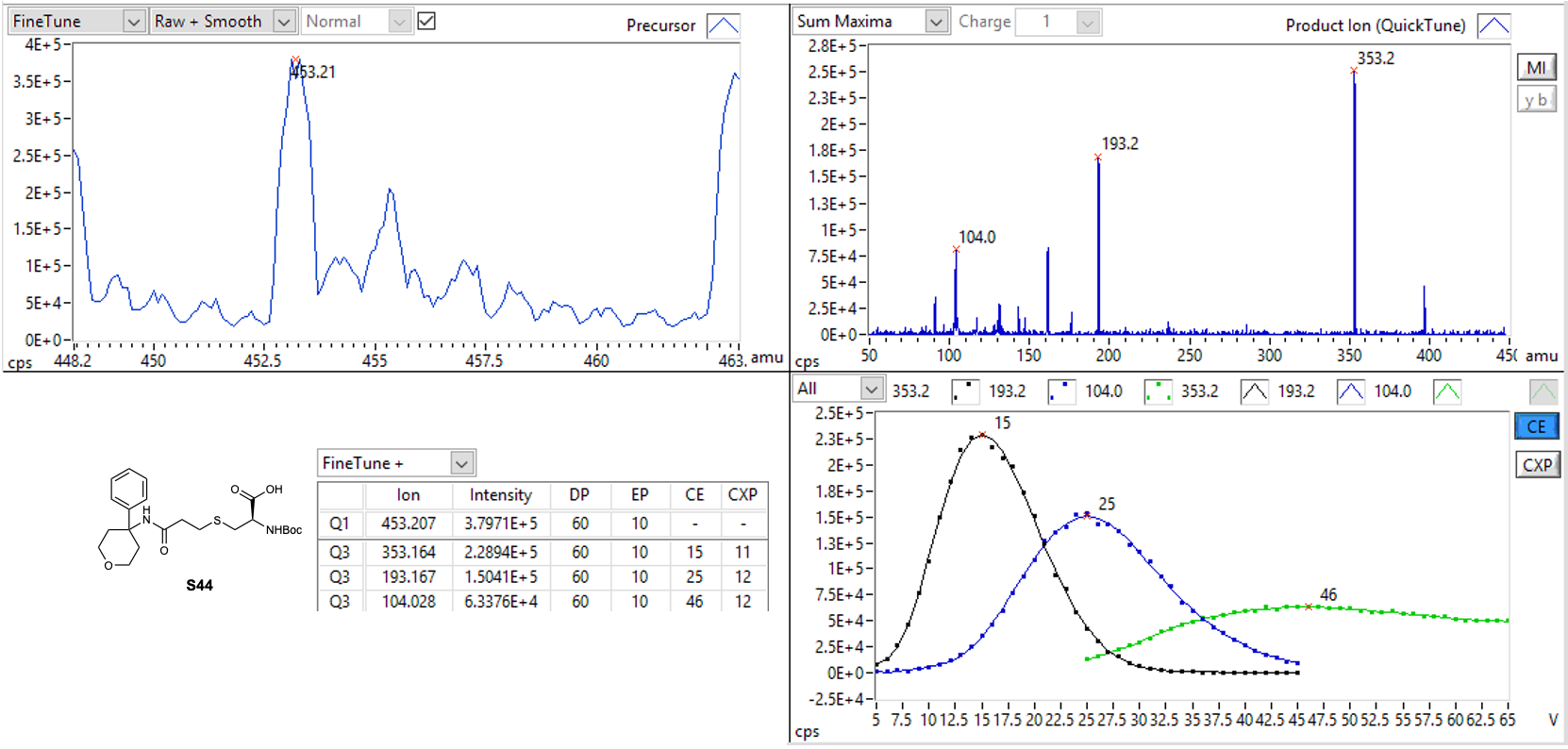


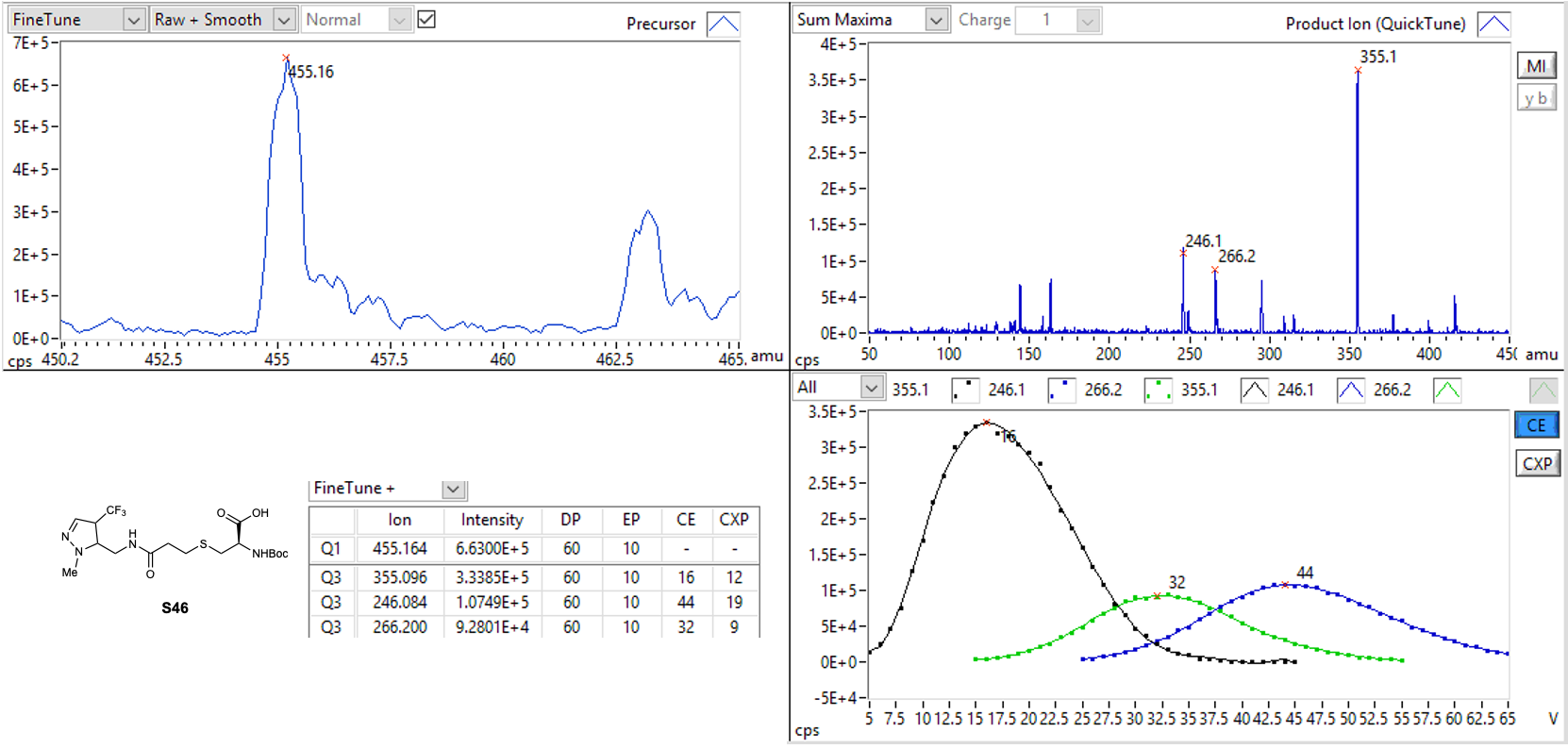


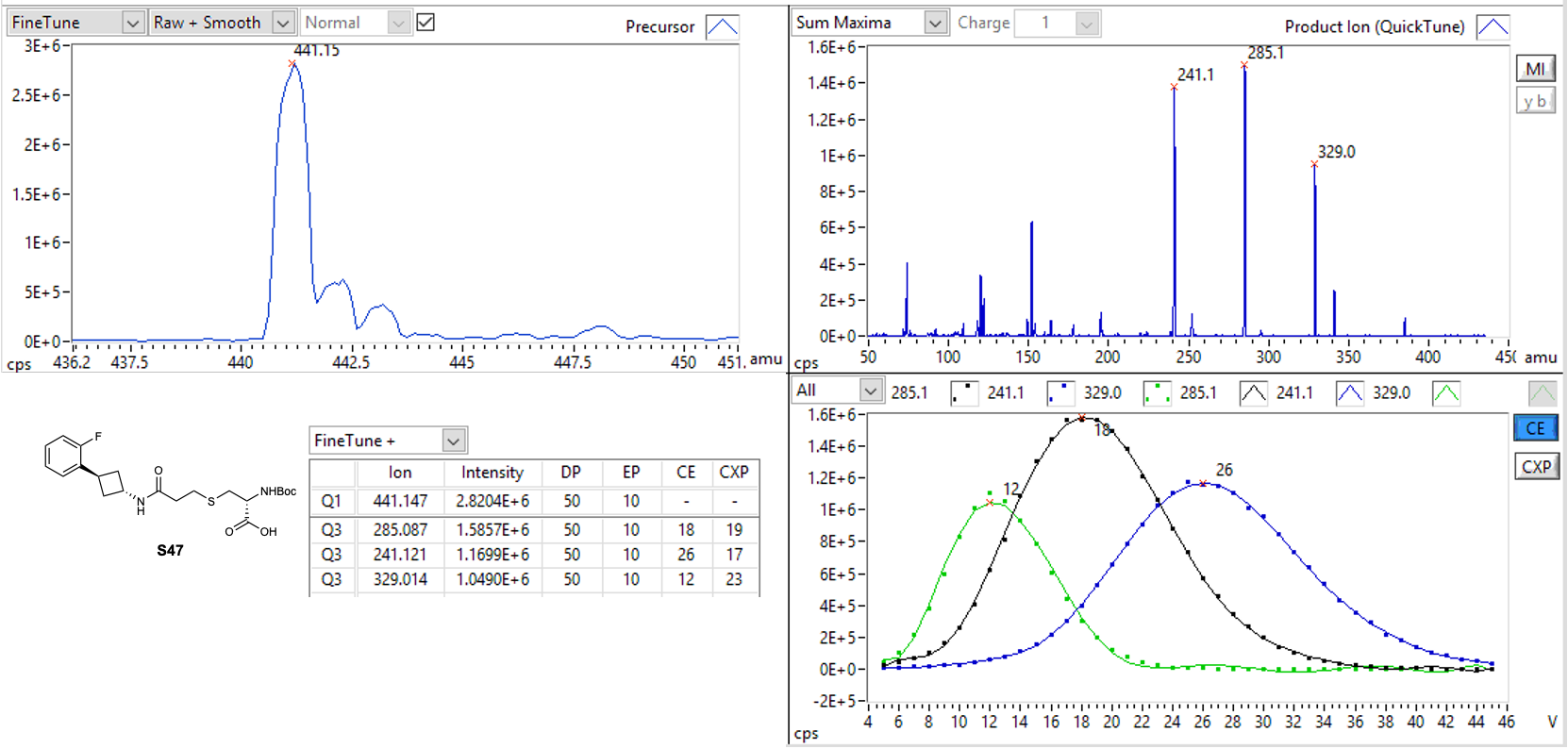


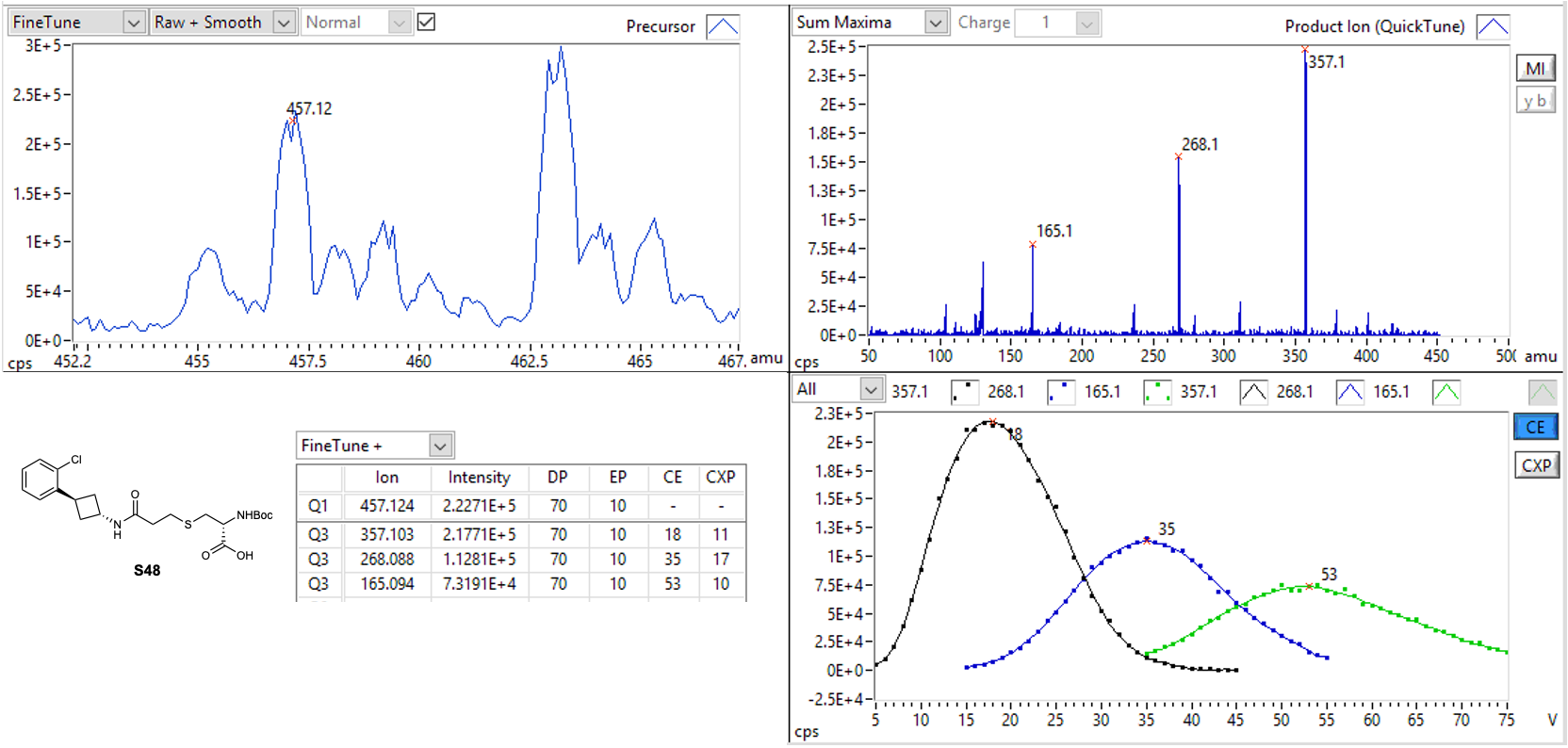


# 5. References

[33] N. Engel, T. Hoffmann, F. Behrendt, P. Liebing, C. Weber, M.l Gottschaldt, and U. S. Schubert, Cryogels based on poly (2-oxazoline)s through development of bi-and trifunctional cross-linkers incorporating end groups with adjustable stability, *Macromolecules* **57**, 2915-2927, (2024).

# 6. NMR Spectra

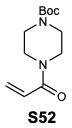

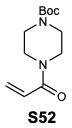

Supplement: Supplementary file 1 — The authors have cited additional references within the Supporting Information [36]. Supporting File: anie72797‐sup‐0001‐SuppMat.docx. [file ANIE-65-e8895173-s001.docx]
